# Insights into structural defect formation in individual InP/ZnSe/ZnS quantum dots under UV oxidation

**DOI:** 10.1038/s41467-024-45944-2

**Published:** 2024-02-23

**Authors:** Hayeon Baek, Sungsu Kang, Junyoung Heo, Soonmi Choi, Ran Kim, Kihyun Kim, Nari Ahn, Yeo-Geon Yoon, Taekjoon Lee, Jae Bok Chang, Kyung Sig Lee, Young-Gil Park, Jungwon Park

**Affiliations:** 1 School of Chemical and Biological Engineering, Institute of Chemical Processes, Seoul National University, Seoul, Republic of Korea; 2 Center for Nanoparticle Research, Institute for Basic Science (IBS), Seoul, Republic of Korea; 3 Samsung Display Co., Ltd., Yongin-si, Gyeonggi-do Republic of Korea; 4 Institute of Engineering Research, College of Engineering, Seoul National University, Seoul, Republic of Korea; 5 Advanced Institute of Convergence Technology, Seoul National University, Suwon, Republic of Korea

**Keywords:** Quantum dots, Characterization and analytical techniques

## Abstract

InP/ZnSe/ZnS quantum dots (QDs) stand as promising candidates for advancing QD-organic light-emitting diodes (QLED), but low emission efficiency due to their susceptibility to oxidation impedes applications. Structural defects play important roles in the emission efficiency degradation of QDs, but the formation mechanism of defects in oxidized QDs has been less investigated. Here, we investigated the impact of diverse structural defects formation on individual QDs and propagation during UV-facilitated oxidation using high-resolution (scanning) transmission electron microscopy. UV-facilitated oxidation of the QDs alters shell morphology by the formation of surface oxides, leaving ZnSe surfaces poorly passivated. Further oxidation leads to the formation of structural defects, such as dislocations, and induces strain at the oxide-QD interfaces, facilitating In diffusion from the QD core. These changes in the QD structures result in emission quenching. This study provides insight into the formation of structural defects through photo-oxidation, and their effects on emission properties of QDs.

## Abstract (web summary)

InP/ZnSe/ZnS quantum dots (QDs) are promising candidates for advanced light-emitting diodes, but low emission efficiency due to oxidation hampers applications. Here, the authors provide insight into the structural defects that form on individual QDs during UV-facilitated oxidation.

## Introduction

Indium phosphide (InP) quantum dots (QDs) represent promising next-generation light-emitting sources due to their wide color gamut, narrow emission bandwidth, and nontoxicity. However, the oxophilic properties of InP make InP-based QDs vulnerable to oxidation, which can diminish their emissions over time^1–4^. To delay oxidation and improve photostability, core/shell-structured QDs have been developed by passivating the InP core with wide-band-gap semiconductors, such as zinc selenide (ZnSe) and zinc sulfide (ZnS)^5–9^. While the ZnS shell protects the QDs from oxidation and provides effective exciton confinement, the large lattice mismatch between InP and ZnS can cause surface traps, thereby reducing photoluminescence (PL) efficiency^7,10–12^. A mid-shell ZnSe passivation layer can alleviate this issue by efficiently passivating the surface of the InP core before ZnS coating^7,10,11,13–15^. This reduces the lattice mismatch and enables near-unity PL efficiency of the InP/ZnSe/ZnS core/shell/shell QDs. However, emission quenching under ambient illumination conditions remains an inherent problem in InP/ZnSe/ZnS QDs, despite the implementation of shell passivation^16,17^.

Structural defects induce the formation of defect states in the electronic structure of QDs, resulting in the degradation of photostability^18,19^. Upon oxidation of the QDs, the formation of oxidized species can lead to structural defects in the QD lattice due to the larger unit cell of the oxidized species compared to that of the pristine QD lattice^20,21^. Emission quenching of QDs is often attributed solely to deep exciton traps generated by the formation of oxidized In species, such as InPO_x_^3,18^, as investigated using spectroscopic methods such as nuclear magnetic resonance (NMR) spectroscopy and X-ray photoelectron spectroscopy (XPS). Solid-state NMR spectroscopy of InP/ZnSe/ZnS has revealed the presence of InPO_x_^2,22^, which formed during the ZnS and ZnSe passivation processes. Additionally, XPS investigations of the InP/ZnS QDs have shown that oxidation during synthesis results in the formation of InPO_x_^3,22^. While these studies have identified the chemical states of oxidation-related species, the mechanism for the formation of structural defects and their effect on the entire QD structure are relatively less understood. This is because the measurements rely on the spectroscopic information obtained from the nanoparticle ensemble. Therefore, to obtain a clear picture of the chemical and structural origins of the photooxidation of QDs, it is necessary to observe the atomic-scale crystal structures, defects, and elemental distributions of individual QDs^23–25^.

In this study, we investigate the formation of structural defects in single InP/ZnSe/ZnS core/shell/shell QDs, which exhibits emission quenching from ultraviolet (UV)-facilitated oxidation, using atomically resolved transmission electron microscopy (TEM). Oxidation of the ZnS shell leads to the formation of ZnO, resulting in a lattice mismatch with the host QD lattice, and consequently the formation of dislocations and strain in the QD. These dislocations induce the diffusion of In atoms from the core over the QD. Additionally, ZnO is easily etched from the QD, and poor passivation of the ZnSe layer leads to the formation of dangling bonds. These findings are further confirmed by high-resolution TEM images of identical QD particles before and after photooxidation.

## Results

### Emission quenching of InP/ZnSe/ZnS QDs under UV exposure

We investigate the UV-facilitated oxidation of the colloidal solution of InP/ZnSe/ZnS QDs which emit red light at the wavelength of 627 nm (Suppl. Fig. 1). These QDs consist of an approximately 3.6-nm-diameter InP core, a 2-nm-thick ZnSe shell, and an approximately 0.2-nm-thick outermost ZnS shell. The QDs are further passivated using oleic acid ligands for dispersion in organic solvents (see Methods). We expose the QD solution to UV light with an emission wavelength of 365 nm and a power density of 610 μW cm^−2^ in the air to facilitate the emission quenching of the QDs, diluting the QD solution with toluene to approximately 5.87 × 10^−4^ mg mL^−1^ before UV exposure (see Methods). UV irradiation in air is known to accelerate the oxidation of various QDs, including InP- and Cd-based QDs, and is frequently used to investigate the emission stability of colloidal QD solutions^9,16,26–28^.

**Fig. 1:**
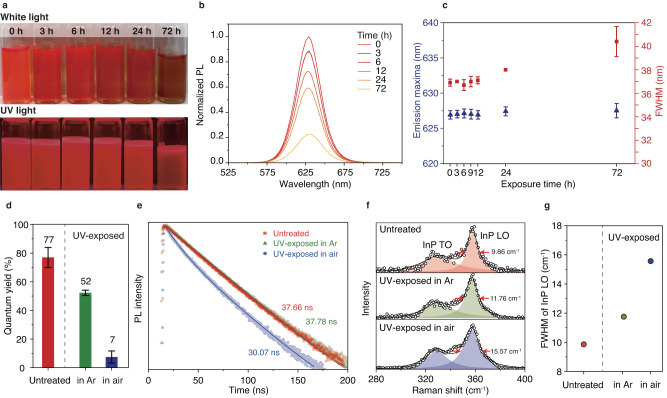
Photobleaching of an InP/ZnSe/ZnS QD solution under UV illumination. **a** Images of the QD solutions before UV exposure and after UV treatment under ambient conditions. Images taken under ambient illumination (White light) and UV illumination (UV light). **b** PL emission spectra of the QD solutions after UV exposure over different times. **c** Changes in the emission peak maxima (blue triangle) and FWHM of the PL emission spectra (red square) in **b**. PL emission spectra is obtained by 10 separated measurements for each UV exposure circumstances, and the error bars indicate the standard deviations. **d** PLQY of untreated QD solution (red column), QD solution exposed to UV in air (blue column), and QD exposed to UV in Ar atmosphere (green column). Gray dotted line separates untreated and UV-exposed QD solutions. The PLQY values are the mean of the 10 separated measurements, and the error bars indicate the standard deviations. **e** PL lifetime changes after UV exposure in Ar and in air. **f** Raman spectra of InP/ZnSe/ZnS QDs after UV exposure in Ar and in air. **g** FWHM of the InP LO peaks in **f**. Each circle represents the FWHM of InP LO, measured in untreated (red circle), QD solution exposed to UV in air (blue circle) and QD solution exposed to UV in Ar (green circle). Gray dotted line separates untreated and UV-exposed QD solutions.

The emission properties of the InP/ZnSe/ZnS QD solution degrade upon UV irradiation in the air. Photographs of the red QD solutions exposed to UV light for periods ranging from 0 to 72 h are shown in Fig. 1a. The QD solution before UV-facilitated oxidation exhibits clear red emission. As the UV exposure time increases, the QD solution gradually loses its original red color and eventually becomes brownish after 72 h of UV exposure (white light, Fig. 1a). Additionally, the emission intensity of the QD solution, inspected using UV excitation, decreases gradually with increasing UV exposure time, and the QD solution becomes almost non-emissive at 72 h (UV light, Fig. 1a). In accordance with these visual inspections, the maximum intensity of the PL emission, measured at 627 nm with an excitation wavelength of 500 nm (Methods), decreases gradually and reaches approximately 20% of its initial value after 72 h of UV exposure (Fig. 1b, c). The measured PL quantum yield (PLQY) of the QD solution shows an approximately 70% reduction after 72 h of the UV exposure, from 77 ± 3% to 7 ± 3%, as shown in Fig. 1d (see Methods). These results indicate that UV exposure in air reduces the radiative emission in the InP/ZnSe/ZnS QD solution^17^.

The reduced radiative emission from the InP/ZnSe/ZnS QDs upon UV exposure in air is caused by oxidation of the QDs. We conduct control experiments in which the QD solutions are exposed to UV light in an Ar atmosphere (see Methods). UV exposure in an Ar atmosphere yields a mild reduction in PLQY of the QD solution after 72 h of exposure, from 77 ± 3% to 52 ± 1% (green bar, Fig. 1d), which is much smaller than the change in the PLQY caused by UV exposure in air. These results correspond with those of previous reports in which the oxidation of various QDs leads to deteriorated emission properties. The long-term oxidation of CdSe and CdS QDs generates a surface oxidized layer that acts as an electronic trap^16,27^. Furthermore, the Cd^2+^ and SeO_2_ on the surface of CdSe QDs dissociate into the solvent, leaving dangling bonds that can form electronic traps^16,27,29^. The presence of these electronic traps, which generates alternative emission pathways, is reflected in the emission properties of the QDs^20,30^.

InP/ZnSe/ZnS QDs oxidized by UV irradiation exhibit emissions mediated by trap states. While the maximum PL emission wavelength, *λ*_max,em_, of the QDs remains similar around 627 nm during the oxidation from 0 to 72 h (blue triangle, Fig. 1c), the full width at half maximum (FWHM) of the emission peak increases from 37 to 41 ± 1 nm, as shown as red square in Fig. 1c. Such broadening of the emission peak is consistent with the previous observations where impurities in the lattice of the ZnSe shell induce emission broadening of InP/ZnSe core/shell QDs^20^. The inclusion of impurities, such as indium species, within the ZnSe shell is known to induce the formation of shallow hole traps, resulting in a broad emission from the QD^30–32^. To further confirm the generation of trap states in the oxidized QDs, we measure the lifetimes of the charge carriers by time-resolved PL spectroscopy (see Methods) and fit the PL decay curves by a tri-exponential function (Suppl. Figure 2 and Suppl. Table 1). The mean PL lifetime of the UV-exposed QDs decreases from 37.66 to 30.07 ns after 72 h of UV exposure in air, as shown in Fig. 1e. However, the QDs exposed to UV in an Ar atmosphere exhibit mostly identical PL lifetimes after 72 h, changing from 37.66 to 37.87 ns (green symbols, Fig. 1e). The PL lifetime can decrease when there is an additional emission process following the charge transfer between the neutral core and the charged surface trap^10,21,33–35^. The oxidation of QDs generates surface dangling bonds and structural disorders associated with lattice defects, forming surface traps^3,20,36^.

### Defect formation in photo-oxidized QDs

Raman spectroscopy provides the degree of lattice disorder based on the reduced symmetry after the formation of structural defects such as interstitials, vacancies, and substitutions^20,37–39^. We use Raman spectroscopy to clarify the lattice dynamics and emission quenching of InP/ZnSe/ZnS QDs after UV-facilitated oxidation (Methods). The InP optical resonance peaks of the Raman spectra of the QDs with different treatments (untreated, UV-exposed in air, and UV-exposed in an Ar atmosphere) are inspected, as shown in Fig. 1f. The broadening of the InP optical resonance peaks indicates the degree of disorder in the InP-based QDs^20^. The transverse optical (TO) resonance and longitudinal optical (LO) resonance peaks of InP are located at approximately 327 and 355 cm^−1^, respectively, which are in accordance with reported values^20,38–40^. The FWHM of the LO peak of the UV-exposed QDs increases from 9.86 to 15.57 cm^−1^ after ambient UV exposure. This change is considerably more significant than that from UV exposure in an Ar atmosphere, from 9.86 to 11.76 cm^−1^ (Fig. 1g). The increase in the FWHM of the LO peak indicates the formation of structural disorder at the InP core and ZnSe mid-shell interface^20^. We also employ X-ray diffraction (XRD) to investigate the formation of structural defects. The evolution of small peaks near 2*θ* = 46.75° and 55.86°, which correspond to (0$\bar{1}$2) and ($\bar{1}\bar{1}$0) lattice reflections of ZnO, is observed after the UV-facilitated oxidation of the InP/ZnSe/ZnS QDs (Suppl. Figure 3). The peaks are barely detectable, implying that the proportion of defects from oxidation is small and probably are localized within the QD domains.

### Observation of structure defect formation in single QDs

**Fig. 2:**
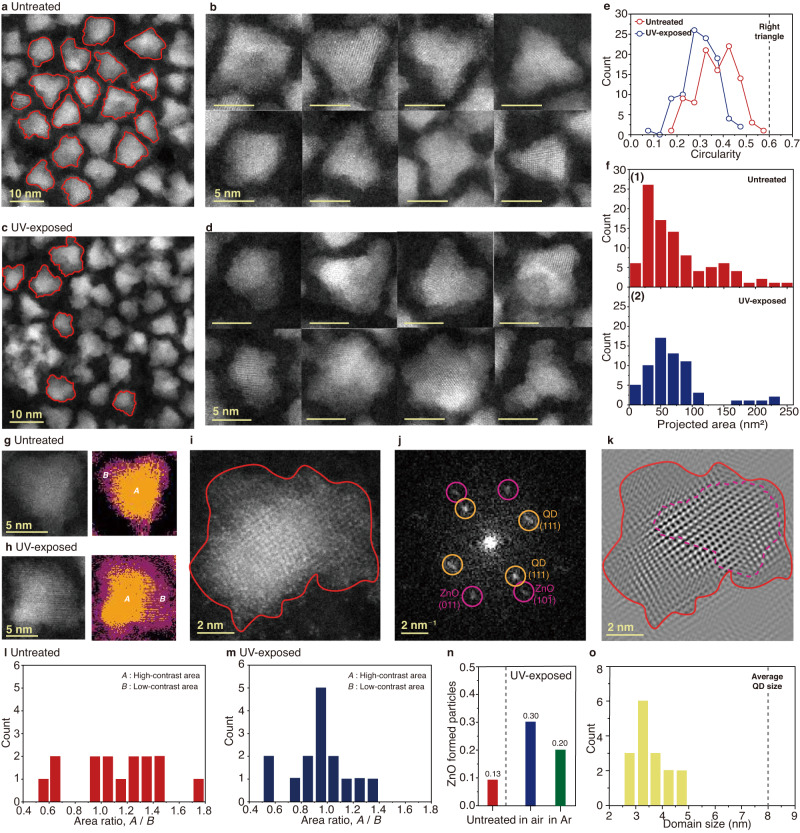
Crystal structure change of QDs by oxidation. **a** HAADF-STEM image of the QDs before the UV-facilitated oxidation. Contours of the QDs used for the calculation of circularity (red line). **b** Enlarged images of representative QDs before the UV-facilitated oxidation. **c** HAADF-STEM image of the QDs after the UV-facilitated oxidation for 72 h. Contours of the QDs used for the calculation of circularity (red line). **d** Enlarged images of representative QDs after the UV-facilitated oxidation. **e** Circularity distributions of QDs before (red symbols) and after (blue symbols) the UV-facilitated oxidation. The dashed line indicates circularity of a right triangle, 0.61. **f** Distributions of 2D projected area of QDs before **(1)** and after **(2)** the UV-facilitated oxidation. **g**, **h** HAADF-STEM images (left) and corresponding binarized images (right) showing high-contrast and low-contrast regions in QDs before **g** and after the UV-facilitated oxidation **h**. High-contrast and low-contrast regions are denoted with *A* (orange color) and *B* (purple color), respectively. **i**–**k** HAADF-STEM image of an oxidized QD taken at a higher magnification **i**, corresponding FFT **j**, and inverse FFT **k** generated from the marked peaks in **j**. (red lines) Contours of the QD. (purple line) Detected ZnO subdomain. **l**, **m** Areal ratio of high-contrast regions to low-control region of the QDs before **l** and after **m** the UV-facilitated oxidation. **n** Ratio of oxidized particles in untreated QDs (red bar), QDs exposed to UV in air (blue column), and QD exposed to UV in Ar atmosphere (green bar). The gray dashed line separates untreated and UV-exposed QDs. **o** Domain size distribution of ZnO subdomains. The gray dashed line indicates the average size of the QDs.

We conduct further analysis of the individual QDs, based on atomically resolved scanning TEM (STEM), energy-dispersive X-ray spectroscopy (EDS), and electron energy loss spectroscopy (EELS) to investigate the identity and location of defects and their effects on the overall crystal structures of the individual QDs (see Methods). HAADF-STEM images of the InP/ZnSe/ZnS QDs before and after 72 h of UV exposure are presented in Fig. 2. The untreated QDs mostly exhibit shapes similar to triangles, the projections of a tetrahedral morphology (Fig. 2a, b), and consist of mostly single-crystalline domains, as shown in high-resolution high-angle annular dark-field (HAADF) STEM images (Fig. 2b and Suppl. Fig. 4). The UV-exposed QDs mostly exhibit more irregular shapes with rough surface and large size variations compared to the untreated QDs (Fig. 2c, d and Suppl. Fig. 4). The difference in the particle morphology and surface structures is more clearly indicated from the 2D projected areas and circularities of the QDs, measured by identifying their contours marked with the red lines in Fig. 2a, c (see Methods). In the UV-exposed QDs, the circularities measured for approximately 100 QD particles have smaller values (Fig. 2e, blue symbols) compared to those of the untreated QDs (Fig. 2e, red symbols) and a reference value for a right triangle (Fig. 2e, dashed line), due to the rough surface characteristic of the UV-exposed QDs (Suppl. Figs. 4 and 5).

QDs also exhibit the changes in their sizes as well as the morphological changes as a result of the oxidation. The 2D projected areas of the untreated QDs ranges from 0 to 100 nm^2^ and the area distribution becomes broader by having populations around 250 nm^2^ after the UV exposure in the air, as shown in Fig. 2f. The size increase of the UV-exposed QDs is mainly due to the increased domains with low image contrast in the HAADF-STEM images (Fig. 2g, h). Image contrast in HAADF-STEM images is correlated with the mean atomic number of a material, allowing the identification of materials with different atomic numbers from the image. We perform binarization of HAADF-STEM images of untreated and UV-exposed QDs to identify high- and low-contrast regions, as marked with *A* and *B*, respectively, in the right panels of Fig. 2g, h and Suppl. Fig. 6. Binarized images show that the low-contrast area increases in the UV-exposed QDs (Fig. 2h, purple regions), compared to the high-contrast area (Fig. 2h, orange regions), indicating the formation of low-atomic-number materials on the surface of the QDs by oxidation. Indeed, crystalline phase analysis in fast Fourier transforms (FFT) reveals that ZnO domains are observed in low-contrast regions of those QDs (Fig. 2i–k). In the FFT of the HAADF-STEM images of the oxidized QD, the peaks correspond to wurtzite ZnO (Fig. 2j, purple circles) and ZnSe or ZnS (Fig. 2j, orange circles) can be identified, and the inverse FFT generated from those peaks reveals a ZnO domain in the QD (Fig. 2k). The surface of QDs is indicated as red line, and the ZnO domain is located at the right side of the QD with a dashed purple line in the representative QD shown in Fig. 2k. The formation of ZnO domains on the oxidized QDs is also found in multiple QDs as shown in the areal ratio of high-contrast regions to low-contrast regions (Fig. 2l–m). Furthermore, multiple images are inspected by the crystalline phase analysis to identify ZnO domains in QDs (Suppl. Fig. 7 and Methods), and the population of the QDs with ZnO on their surfaces increases after the UV exposure in air (Fig. 2n). The size distribution of the ZnO subdomains is shown in Fig. 2o. On the other hand, the QDs exposed to UV in an Ar atmosphere exhibit little morphological changes compared to the untreated QDs, as shown in Suppl. Figs. 5 and 8. Small amount of ZnO domains are identified in these QDs exposed to UV in Ar (Fig. 2n, green bar), possibly formed by a trace amount of oxygen in the solvents and is attributed to the decrease in the PLQY (Fig. 1d).

**Fig. 3:**
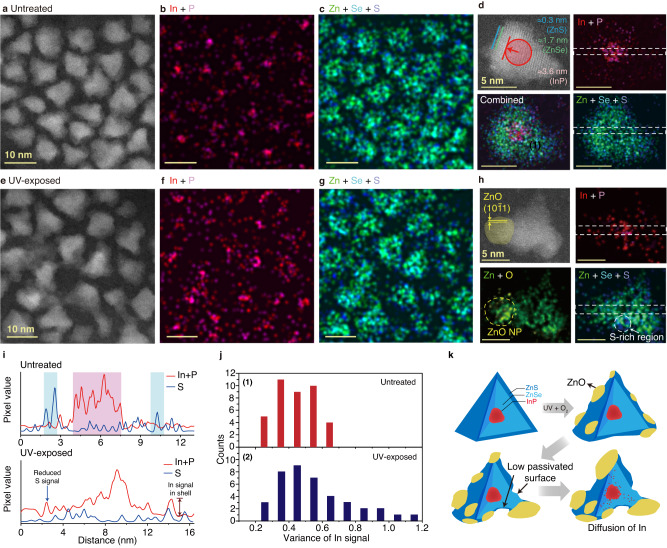
Chemical structure change of QDs by oxidation. **a** HAADF-STEM image of the QDs before oxidation**. b**, **c** Corresponding EDS elemental maps for In (red) and P (purple) **b** and for Zn (green), Se (cyan), and S (blue) **c**. **d** EDS analysis of single QD before oxidation taken at a higher magnification. HAADF-STEM image (upper left panel), EDS elemental map (upper right panel) for In and P, EDS elemental map for Zn, Se, and S (lower right panel), EDS elemental map showing all the elements (lower left panel). **e** HAADF-STEM image of the QDs after oxidation. **f**, **g** Corresponding EDS elemental maps for In (red) and P (purple) **f** and for Zn (green), Se (cyan), and S (blue) **g**. **h** EDS analysis of single QD after oxidation taken at a higher magnification. HAADF-STEM image (upper left panel), EDS elemental map for In and P (upper right panel), EDS elemental map for Zn, Se, and S (lower right panel), EDS elemental map showing all the elements (lower left panel). **i** Line profiles extracted from the EDS map for In and P along the line marked in **d**, **h**. **j** Variance of In signals in the EDS elemental maps, measured for QD before **(1)** and after **(2)** the UV-facilitated oxidation. **k** Schematic illustration for the proposed degradation mechanism of InP/ZnSe/ZnS QDs by the UV-facilitated oxidation. UV-facilitated oxidation induces oxidation of ZnS shell, forming ZnO domain on the QD surface. The ZnO is partially etched away, induces poor passivation of the ZnSe surface. The ZnO domains induces lattice defects within the QD, which serves as a path for the diffusion of In.

Another interesting change to note is the elemental distribution changes in the QDs after UV exposure in air. EDS elemental maps show that the untreated QDs exhibit a well-defined InP/ZnSe/ZnS core/shell/shell structure (Fig. 3a–d). The InP core is spherical with a diameter of approximately 3.6 nm and is located at the center of the QD. The core is covered with a 1.7-nm-thick ZnSe shell, which is fully passivated by a 0.3-nm-thick ZnS shell (Fig. 3d). In the EDS maps of the QDs exposed to UV in air, In and P signals are severely delocalized from the QD center (Fig. 3e, f). The EDS elemental maps indicate that the interface between the InP core and ZnSe shell in the oxidized QD is not as clear as that in the untreated QD (Fig. 3g, h), also shown in the line profiles extracted from the EDS elemental maps (Fig. 3i). These results are confirmed by comparing the variance of the In signals over the EDS maps of single QDs for 50 untreated and oxidized QDs (Fig. 3j and Methods). On the other hand, the In remains in the core region after UV exposure in an Ar atmosphere, suggesting that the QD oxidation is strongly associated with the diffusion of In species (Suppl. Fig. 9). The observed In diffusion possibly induces partial alloying at the core-shell interface which is known to act as a charge carrier trap^31,41^. Furthermore, the distribution of S atoms in the EDS maps of the oxidized QD is largely inhomogeneous, owing to the exposure of S-rich and S-poor regions at the QD surface (Fig. 3h, i, Suppl. Figs. 10 and 11), because of the oxidation of the outermost ZnS shell.

The oxidized QDs are composed of multiple domains, as indicated in the HAADF-STEM images (Fig. 2), which lead to lattice strain at domain interfaces. This is better visualized in the inverse FFT in Suppl. Fig. 12a,b and the strain map obtained by geometric phase analysis (GPA) in Suppl. Fig. 12c. The lattice strain is induced by the large lattice parameter difference between ZnS and ZnO (2.85 Å for ZnO and 3.12 Å for ZnS), which is sufficient enough for the strain formation in the interface^19,42,43^. The strain in the multiple domains can facilitate diffusion of elements^44,45^. Therefore, it is possible that diffusion of In can occur, affected by the strain in oxidized QDs.

The annular dark-field (ADF) STEM image and overlaid EELS elemental maps of the UV-exposed QD further reveal the presence of In diffused along the dislocations within the oxidized QD. The ADF-STEM image shows a dislocation in a UV-exposed QD, which is marked with a yellow dashed line and an arrow with a light blue color (Suppl. Fig. 13a). The formation of dislocations is also confirmed by the splitting of the crystalline peaks in the corresponding FFT, marked with the green and pink circles in the yellow box (Suppl. Fig. 13b). The ADF images of the entire QD (blue spot) are combined with the EELS map of In (yellow spot) to observe the distribution of In atoms in the QD (Suppl. Fig. 13c). The In, that diffused from the core of the QD, is detected near the dislocation line. Interestingly, dislocations are observed near the QD surface where O is detected (Suppl. Fig. 13d), indicating that the formation of dislocations within the QD is initiated by surface oxidation. Dislocations often serve as a path for the diffusion of elements in crystals^46–48^, supporting that the diffusion of In can be affected by the presence of dislocations. Summarizing our observations, the oxidation-induced degradation process of InP/ZnSe/ZnS QDs can be described as follows (Fig. 3k): (i) oxidation of a InP/ZnSe/ZnS QD induces the formation of ZnO domains on the QD surface. ii) The formation of surface ZnO domains leads to partial etching of ZnS layers to form poorly passivated ZnSe layers. (iii) The ZnO domains also deform the lattice structure of the QD and form dislocation within the QD, which serves as a path for the diffusion of In species.

It is well known that structural defects and emission efficiency of QDs are closely related^2,16,18,20^, but the types of such structural defects are less reported. Our STEM observations show the formation of ZnO domains, S-poor QD surfaces with dangling bonds, dislocations, and partially alloyed core-shell interface in the oxidized QDs. These structural features identified in the oxidized QD can contribute to charge carrier traps and deteriorated emission efficiency of the oxidized QDs. For example, alloying of InP core and ZnSe shell is known to generate hole traps near the valence band edge^31^; dangling bonds of ZnSe^49^ at the S-poor QD surfaces and at the interfaces with ZnO also act as surface trap sites^19,36,50–52^; point defects, strain and dislocations are well-known sources for charge carrier traps which deteriorate the emission efficiency of QDs^8,19,20,43–45,50^.

### Structural changes of identical QDs after photo-oxidation

**Fig. 4:**
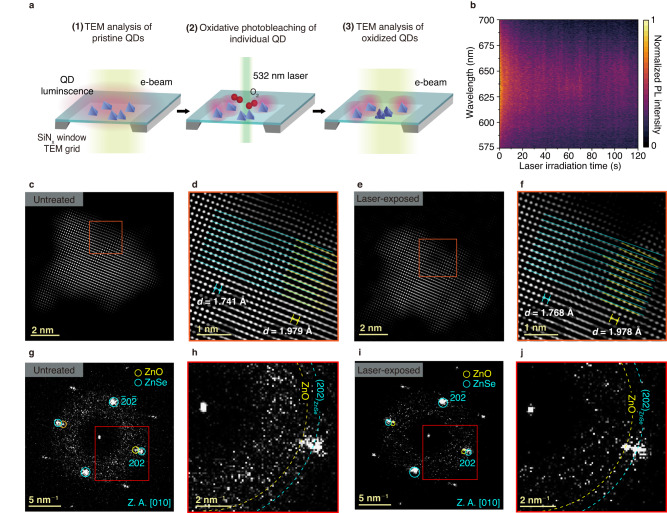
ILTEM of a QD correlated with the PL measurement. **a** Schematic illustration showing the ILTEM experiment process; **(1)** TEM images of initial QDs are taken with low electron dose to minimize electron-beam induced damage. **(2)** Laser-exposure on the QDs induces photo-oxidation, and the tracking of the PL spectra is simultaneously obtained. **(3)** TEM images of oxidized QDs are obtained after the laser-induced oxidation. E-beam (lime column), laser (green column) and QD luminescence (red circles) are indicated in **a. b** In situ PL measurement of QD. **c**, **d** Inverse FFT of a untreated QD **c** and magnified view **d** of the boxed region in **c**. **e**, **f** Inverse FFT of the same QD after laser exposure **e** and magnified view **f** of the boxed region in **e**. **g**, **h** FFT **g** used to obtain **c**, and magnified view **h** of the boxed region in **g**. **i**, **j** FFT **i** used to obtain **e**, and magnified view **j** of the boxed region in **i**. Blue and yellow arc corresponds to the lattice spacing of ZnSe and oxidized part, respectively.

The identical-location TEM (ILTEM) technique is used to further understand the evolution of structural defects in the QD after UV-facilitated oxidation. ILTEM can be used to investigate the structures of the same QDs before and after monitoring the PL efficiency (Fig. 4a). The untreated QD solution is drop-cast onto a TEM grid with a 3-nm-thick Si window and subsequently imaged using high-resolution TEM to investigate the initial QD structure. Low-dose imaging with an electron dose of 500 e^−^ Å^−2^ is performed to minimize electron-beam-induced damage to the samples. After TEM imaging, the QDs on the Si TEM grid are exposed to laser in the air for tracking PL spectra during laser-induced photo-oxidation, as shown in Fig. 4b. Emission quenching of the QDs is observed during laser exposure. The duration of laser irradiation of the QDs is extended until the normalized PL intensity maximum matches that of the UV irradiation cases, equivalent to an approximately 20% reduction in the intensity maximum (Suppl. Fig. 14a). Again, it is confirmed that the PL intensity does not decrease noticeably when the laser is irradiated in an enclosed cell filled with Ar (Suppl. Fig. 14b, c)^53^, similar to the PLQY and PL lifetime measurements of the UV-exposed QDs shown in Fig. 1. After laser irradiation, the same regions are searched and imaged using high-resolution TEM to directly compare the structural changes caused by laser-facilitated oxidation.

The ILTEM images show that the lattice structures of the same InP/ZnSe/ZnS QD become distorted after laser-facilitated oxidation. Before laser exposure, the structure of the QD is mostly single-crystalline and exhibits a clean surface, as shown in the inverse FFT image in Fig. 4c. A magnified view of the QD lattice clearly indicates that the lattice exhibits a cubic zinc blende structure with marginal lattice distortion (Fig. 4d). After laser exposure, the surface of the QD becomes rough (Fig. 4e) owing to oxidation during laser exposure in air. The orange box in Fig. 4e, the same part of the QD shown in Fig. 4c, exhibits significant distortion in the QD lattice (Fig. 4f). The angle in the distorted region is 2.46°, as shown in Fig. 4f. The high-resolution (HR) TEM images of Fig. 4c, e are shown in Suppl. Fig. 15. The FFT patterns of the QD are inspected to identify the regions with different lattice spacing. Minor satellite peaks are identified near the ZnSe peaks in the FFT patterns before laser exposure (Fig. 4g). By measuring the lattice spacing, the satellite peaks are identified as those of ZnO. In the magnified view, the FFT peaks of the ZnO phase are negligible and mostly overlapped with the (202)_ZnSe_ peaks before laser exposure (Fig. 4h). After laser exposure, the FFT peaks of ZnO becomes stronger and are distinctly separated from the (202)_ZnSe_ peaks (Fig. 4i). This can be observed more clearly in the magnified FFT patterns in Fig. 4j. The minor ZnO signature observed in the QDs before laser irradiation is possibly due to atmospheric oxidation during storage and handling of the QD sample (Suppl. Fig. 16). The ILTEM observations clearly show that irradiation with light expedites the oxidation of the QDs. The results from the ILTEM analysis and emission quenching during the laser exposure correspond with the (S)TEM observations of the InP/ZnSe/ZnS QDs oxidized by UV exposure (Fig. 2). These observations indicate that the formation of oxide in the QD that results in lattice deformation can occur ubiquitously in oxygen-containing environments, and such local structural changes affect the optical properties of the QDs.

In summary, structural defect formation and the subsequent propagation of structural changes in InP/ZnSe/ZnS QDs are investigated using high-resolution STEM and ILTEM observations. The oxidized species formed on the QD surface induce the formation of dislocations and strain, which promote the diffusion of In. The oxides on the QD surface further induces poor passivation by ZnSe. The structural defects and species diffused over the QD are responsible for the emission quenching of the QDs. This study provides subparticle-level mechanisms that explain the optical properties and stability of colloidal InP/ZnSe/ZnS QDs, which are widely used in the display industry.

## Methods

### Materials

Tris(trimethylsilyl)phosphine (TMS_3_P, 95%) and trioctylphosphine (TOP, 97%) were purchased from Strem. Indium acetate (In(OAc)_3_, 99.99%), zinc acetate (Zn(OAc)_2_, 99.99%), palmitic acid (PA, 99%), oleic acid (OA, 90%), sulfur (S, 99.99%), selenium (Se, 99.99%), 1-octadecene (ODE, 90%), and trioctylamine (TOA, 98%), toluene (anhydrous, 99.8%), hexane (anhydrous, 95%) were purchased from Sigma-Aldrich.

### Synthesis process

InP/ZnSe/ZnS QD solution was synthesized by Samsung Display Ltd and the procedure for the synthesis of the InP/ZnSe/ZnS QD is reported elsewhere^11,54–56^. Briefly, InP core was synthesized using In(OAc)_3_ and TMS_3_P as In and P precursor, and palmitic acid as ligands. The In(OAc)_3_ and palmitic acid were dissolved in octadecene and then evacuated using a rotary pump. Under the flow of N_2_ gas, the solution was heated to the temperature of 280 °C, at which the TMS_3_P dissolved in TOP was quickly injected to the solution. The InP core crude solution was cleaned for the further shell passivation. For shell passivation, Zn oleate, Zn(OAc)_2_, and oleic acid dissolved in TOA were degassed, and then heated to 280 °C under a N_2_ flow. Then, the temperature was dropped to 180 °C and the InP core solution was injected quickly. Afterward, the mixture was heated again to 340 °C and Zn oleate and Se/TOP were added to the mixture for ZnSe passivation. Finally, Zn oleate and the S/TOP were added to the mixture for further ZnS passivation. The red QD solution was stored inside a glove box (O_2_ < 0.5 ppm) to avoid any oxygen contact.

### UV irradiation process

The red QD solution was diluted from a red QD stock solution (30 wt% or 1.76 × 10^−1^ mg mL^−1^) with toluene to 5.87 × 10^−4^ mg mL^−1^ for UV irradiation experiments. We first diluted stock solution into 5.87 × 10^−3^ mg mL^−1^ and then re-diluted with toluene to 5.87 × 10^−4^ mg mL^−1^. The QD stock solution is highly concentrated, therefore large number of QDs are observable even after the dilution within field of views of (S)TEM used in this study (Suppl. Fig. 17). The red QD solution was diluted with toluene to 5.87 × 10^−4^ mg mL^−1^ for UV irradiation experiments. 3 mL of the diluted QD solution was placed in the 5 mL vial, and then, the UV light (365 nm) was irradiated with a portable UV lamp (VL-6.LC, Vilber) for 3, 6, 12, 24, 72 h. The power of the UV lamp was 6 W and the power density was 610 μW cm^−2^ at 15 cm. The distance between the UV lamp and the vial was approximately 5 cm.

### UV-vis absorption and PL measurements

The red QD solution was diluted with toluene to 5.87 × 10^−4^ mg mL^−1^ and placed in a 1.5 mL quartz cuvette. Absorption spectra were measured by UV-vis spectrometers from 400 nm to 850 nm (Optizen pop, KLAB). Emission spectra were obtained using a fluorospectrometer (Fluoromax plus, Horiba Scientific) with a 150 W Xe lamp. The excitation wavelength for PL emission measurements was 500 nm. The PL emission is obtained for 10 independent QD solution samples for each time of UV exposure, and mean values are indicated with error bars which represent standard deviations (Fig. 1c).

### PLQY measurements and time-resolved photoluminescence decay

The red QD solution was diluted with toluene to 5.87 × 10^−4^ mg mL^−1^ and placed in a 4.0 mL quartz cuvette. The absolute PLQY of diluted QD samples were obtained by Horiba QuantaPhi-2 under the 500 nm excitation. The PLQY values are the mean of the 10 independent measurements, and the error bars indicate the standard deviations (Fig. 1d). A photoluminescence decay was measured for the QD solution diluted in hexane (5.87 × 10^−4^ mg mL^−1^) by time correlated single photon counting (TCSPC) system (DeltaTime, Horiba). The obtained lifetime curves were fitted with triexponential functions using a software (Data station, Horiba Scientific) to obtain mean lifetime of charge carriers. PL decay curves shown in Fig. 1e in the main text were fitted with tri-exponential function, see Eq. (1):

1 $$y={y}_{0}+{A}_{1}{e}^{-t/{\tau }_{1}}+{A}_{2}{e}^{-t/{\tau }_{2}}+{A}_{3}{e}^{-t/{\tau }_{3}}$$

The fitted results are presented in Suppl. Figure 2 and the obtained pre-exponentials, *A*, and lifetimes, *τ*, are presented in Suppl. Table 1. The mean value of the three lifetimes obtained by the fitting of each sample was presented in the Fig. 1e. For comparison, we also fitted the PL decay curves with bi-exponential function, see Eq. (2):

2 $$y={y}_{0}+{A}_{1}{e}^{-t/{\tau }_{1}}+{A}_{2}{e}^{-t/{\tau }_{2}}$$

The fitted results and parameters are also presented in Suppl. Fig. 2 and Suppl. Table 1.

### Raman spectroscopy

The Raman spectra were obtained by using aberration-corrected spectrometer operated with a 532 nm laser (HEDA, NOST) to characterize the optical resonances of InP. For measurements of the InP/ZnSe/ZnS film at the Ar environment, a lab-made inert cell was fabricated using a glass slide and a cover slip in a glove box. First, 50 µL of the diluted QD solution (concentration of 5.87 × 10^−3^ mg mL^−1^, diluted in anhydrous toluene) is drop-casted on a cover slip for 10 times. Next, we applied layers of carbon tapes (NEM tape, Nisshin) on a glass slide to make well-type cell with four edges and empty inside. The height of the well is about 0.16 mm. Then, the QD-deposited side of the cover slip is mounted on the carbon tape well, and the fabricated cell is compressed softly to ensure complete adherence.

### Powder XRD

Powder XRD patterns are obtained with Cu Kα radiation (*λ* = 1.5406 Å) in a reflection geometry on a Rigaku SmartLab X-ray diffractometer operating at 40 kW and 30 mA. The samples are prepared by dropping a diluted QD solution (5.87 × 10^−3^ mg mL^−1^ in toluene) on a Si wafer with the size of approximately 1 × 1 cm^2^. The 50 µL droplet of the diluted QD solution was drop-casted 10 times on the Si wafer and dried in an ambient condition. For the power XRD measurement of a pristine QD film, the specimen was prepared in a glove box to avoid air contact before the XRD experiments.

### TEM and STEM characterization

For the TEM observations, we irradiated UV light to the QD solution diluted in toluene (5.87 × 10^−5^ mg mL^−1^) for 72 h in the ambient condition. The high-resolution TEM images of the QDs were obtained using a JEM-ARM200F (JEOL) microscope equipped with a spherical aberration corrector in the objective lens (image corrector), a cold field emission gun and a K3 IS Base detector (Gatan, U.S.A.). To avoid electron-beam induced sample damages, the dose rate of 500 e^−^ Å^−2 ^s^−2^ was used for the imaging. The HAADF-STEM images, EDS maps, and EELS maps of QD were obtained using a JEM-ARM200F (JEOL) microscope equipped with a spherical aberration corrector in the condenser lens (probe corrector) and a cold field emission gun. The microscopes were operated at 200 kV and installed at the National Center for Inter-university Research Facilities (NCIRF) at Seoul National University.

### Calculation of circularity

Circularity can be used to describe the surface roughness of the irregularly shaped nanoparticles^57^. To investigate the surface roughness of the QDs before and after the UV-facilitated oxidation, we calculated the circularity of the QDs using the HAADF-STEM images. First, the images were binarized. Then, the contours of the QDs were identified from the binarized images, which enabled the calculation of the perimeters and 2D projected area of the QDs. The circularity, *C*, is calculated using Eq. (3).

3 $$C=\frac{A}{{D}^{2}}$$

where *A* indicates the 2D projected area of a particle and *D* indicates the perimeter of the same particle. In HAADF-STEM images of the QDs, aggregated and vertically overlapped QD particles, presumably formed during TEM sample preparation processes, were often found. We excluded those QD particles from the circularity calculation for accurate measurement, and only the isolated QD particles (marked in red contours in Fig. 2a, c) were used for the calculation. 96, 90, and 97 particles were measured for the pristine sample (without a treatment), sample exposed to UV in the air, and sample exposed to UV in an Ar atmosphere, respectively.

### Calculation of In signal variance

The spatial distribution of In species was calculated from the EDS elemental maps for In by defining “variance of EDS signal”. The variance of In signal in the EDS map is an average of the signal intensities weighted by the distance of each signal from the center of mass of the In signal in a 2D EDS map, followed by a normalization. It has a value of 0 if the In signal is detected only at a single pixel in a 2D EDS elemental map, and has a value of 1 if the In signal is uniformly distributed over a 2D map. To calculate the signal variance of In species over a single QD, we first cropped a 2D EDS elemental map for In to include a single QD and then, calculated the locations (*x*_center_, *y*_center_) of center of mass of the In signals in the cropped image. The distance-weighted signal average is calculated by multiplying a matrix describing the distance from a pixel at (*x*_i_, *y*_j_) to the center of mass by the 2D EDS elemental map for In:

4 $$S=\frac{1}{N}\mathop{\sum}\limits_{i,j}\sqrt{{({x}_{i}-{x}_{{{{{{\rm{center}}}}}}})}^{2}+{({y}_{j}-{y}_{{{{{{\rm{center}}}}}}})}^{2}}\cdot I$$

where *I* indicates a 2D EDS elemental map for In, and *N* indicates the total number of pixels in the map. Finally, *S* is divided by the *S*_ref_, which is calculated for a virtual EDS map composed of uniform signals in the same way.

### Detection of ZnO subdomains in the QDs

The identification of ZnO subdomains within individual QDs was achieved through the HAADF-STEM images by using a custom MATLAB script. Initially, a small segment of the image (typically located at upper-left corner at first) was selected. FFT was then performed on this segment (Suppl. Fig. 7a). The size of the segment and HAADF-STEM images were typically 128 × 128 pixels and 512 × 512 pixels, respectively. The ‘matrix’ is constructed to record information about the ZnO subdomains, with the same dimensions as the STEM image. Initially, all values in the ‘matrix’ were zero. From the FFT of a segment, the presence of ZnO was identified (Suppl. Fig. 7b) by detecting a peak corresponding to major lattice spacings of wurtzite ZnO, such as 2.51 Å, 2.65 Å, and 2.85 Å. If crystalline peaks of ZnO were detected in the FFT, a value of “1” was added to the ‘matrix’. This procedure was repeated by sweeping through the entire STEM image, moving 1 pixel from left to right. When the segment reached the edge of the image, it moves to the next row (1 pixel below). The ZnO detection procedure ended when the segment reached the last part of the image, and the ‘matrix’ is visualized as a heat map. The representative result is shown in Suppl. Fig. 7c. Once the rough location of ZnO is identified, an inverse FFT is performed at the position by crystalline ZnO peaks in the FFT pattern to map the regions with ZnO lattices (Suppl. Fig. 7d).

### Reporting summary

Further information on research design is available in the Nature Portfolio Reporting Summary linked to this article.

### Supplementary information

Supplementary Information

Peer Review File

Reporting Summary

## Data Availability

The data that support the findings of this study are available from the corresponding authors upon request.

## Code availability

The codes for the TEM image processing in this study are available from Github (https://github.com/bhy9704/Image-processing-of-nanoparticles), Zenodo^58^, and from the corresponding authors upon request.

## References

[1] Cros-Gagneux Arnaud (2010). Surface chemistry of InP quantum dots: a comprehensive study. J. Am. Chem. Soc..

[2] Tessier MD (2018). Interfacial oxidation and photoluminescence of InP-based core/shell quantum dots. Chem. Mater..

[3] Duan X (2023). Study of the interfacial oxidation of InP quantum dots synthesized from Tris(dimethylamino)phosphine. ACS Appl. Mater. Interfaces..

[4] Tamang S, Lincheneau C, Hermans Y, Jeong S, Reiss P (2016). Chemistry of InP nanocrystal syntheses. Chem. Mater..

[5] Tessier MD, Dupont D, De Nolf K, De Roo J, Hens Z (2015). Economic and size-tunable synthesis of InP/ZnE (E = S, Se) colloidal quantum dots. Chem. Mater..

[6] Huang K (2010). Internal structure of InP/ZnS nanocrystals unraveled by high-resolution soft X-ray photoelectron spectroscopy. ACS Nano.

[7] Cao F (2018). A layer-by-layer growth strategy for large-size InP/ZnSe/ZnS core–shell quantum dots enabling high-efficiency light-emitting diodes. Chem. Mater..

[8] Reid KR, McBride JR, Freymeyer NJ, Thal LB, Rosenthal SJ (2018). Chemical structure, ensemble and single-particle spectroscopy of thick-shell InP-ZnSe quantum dots. Nano Lett..

[9] Fu Y (2017). Excellent stability of thicker shell CdSe@ZnS/ZnS quantum dots. RSC Adv..

[10] Kim Y (2019). Bright and uniform green light emitting InP/ZnSe/ZnS quantum dots for wide color gamut displays. ACS Appl. Nano Mater..

[11] Park J (2022). Tuning hot carrier dynamics of InP/ZnSe/ZnS quantum dots by shell morphology control. Small.

[12] Gong K, Kelley DF (2015). Lattice strain limit for uniform shell deposition in zincblende CdSe/CdS quantum dots. J. Phys. Chem. Lett..

[13] Hahm D (2019). Design principle for bright, robust, and color-pure InP/ZnSexS1–x/ZnS heterostructures. Chem. Mater..

[14] Kim K, Lee H, Ahn J, Jeong S (2012). Highly luminescing multi-shell semiconductor nanocrystals InP/ZnSe/ZnS. Appl. Phys. Lett..

[15] Wang S (2017). Single‐source precursor route for synthesis of high‐quality green‐emitting quantum dots and their hydrophilic surface modification. Bull. Korean Chem. Soc..

[16] Moon H, Lee C, Lee W, Kim J, Chae H (2019). Stability of quantum dots, quantum dot films, and quantum dot light-emitting diodes for display applications. Adv. Mater..

[17] Orfield NJ (2018). Photophysics of thermally-assisted photobleaching in “Giant” quantum dots revealed in single nanocrystals. ACS Nano.

[18] Cho E (2018). Optical characteristics of the surface defects in InP colloidal quantum dots for highly efficient light-emitting applications. ACS Appl. Nano Mater..

[19] Hans J, Queisser, Haller EE (1998). Defects in semiconductors: some fatal, some vital. Science.

[20] Janke EM (2018). Origin of broad emission spectra in InP quantum dots: contributions from structural and electronic disorder. J. Am. Chem. Soc..

[21] Van Sark WGJHM (2001). Photooxidation and photobleaching of single CdSe/ZnS quantum dots probed by room-temperature time-resolved spectroscopy. J. Phys. Chem. B.

[22] Virieux H (2012). InP/ZnS nanocrystals: coupling NMR and XPS for fine surface and interface description. J. Am. Chem. Soc..

[23] Kim Y (2019). III-V colloidal nanocrystals: control of covalent surfaces. Chem. Sci..

[24] Ren P (2021). Atomic gradient structure alters electronic structure in 3D across the bulk and enhances photoactivity. Adv. Energy Mater..

[25] Kwag J, Kim S, Kang S, Park J (2023). Multiple‐length scale investigation of Pt/C degradation by identical‐location transmission electron microscopy. Bull. Korean Chem. Soc..

[26] Aldana J, Wang YA, Peng X (2001). Photochemical instability of CdSe nanocrystals coated by hydrophilic thiols. J. Am. Chem. Soc..

[27] Spanhel L, Haase M, Weller H, Henglein A (1987). Photochemistry of colloidal semiconductors. 20. Surface modification and stability of strong luminescing CdS particles. J. Am. Chem. Soc..

[28] Peng X, Schlamp MC, Kadavanich AV, Alivisatos AP (1997). Epitaxial growth of highly luminescent CdSe/CdS core/shell nanocrystals with photostability and electronic accessibility. J. Am. Chem. Soc..

[29] Hines DA, Becker MA, Kamat PV (2012). Photoinduced surface oxidation and its effect on the exciton dynamics of CdSe quantum dots. J. Phys. Chem. C.

[30] Raino G (2022). Ultra-narrow room-temperature emission from single CsPbBr(3) perovskite quantum dots. Nat. Commun..

[31] Freymeyer NJ (2020). Effect of indium alloying on the charge carrier dynamics of thick-shell InP/ZnSe quantum dots. J. Chem. Phys..

[32] Zhang K, Chang H, Fu A, Alivisatos AP, Yang H (2006). Continuous distribution of emission states from single CdSe/ZnS quantum dots. Nano Lett..

[33] Cordones AA, Leone SR (2013). Mechanisms for charge trapping in single semiconductor nanocrystals probed by fluorescence blinking. Chem. Soc. Rev..

[34] Galland C (2012). Lifetime blinking in nonblinking nanocrystal quantum dots. Nat. Commun..

[35] Galland C (2011). Two types of luminescence blinking revealed by spectroelectrochemistry of single quantum dots. Nature.

[36] Ruiz Alvarado IA, Karmo M, Runge E, Schmidt WG (2021). InP and AlInP(001)(2 x 4) surface oxidation from density functional theory. ACS Omega.

[37] Rao CSR, Sundaram S, Schmidt RL, Comas J (1983). Study of ion‐implantation damage in GaAs:Be and InP:Be using Raman scattering. J. Appl. Phys..

[38] Bedel E (1986). Characterization of implantation and annealing of Zn-implanted InP by Raman spectrometry. J. Appl. Phys..

[39] Bedel E, Landa G, Carles R, Renucci JB (1985). Raman investigation of the InP lattice dynamics. J. Phys. C: Solid State Phys..

[40] Cavanaugh P (2021). Resonance Raman study of shell morphology in InP/ZnSe/ZnS core/shell/shell nanocrystals. J. Phys. Chem. C.

[41] Cho H, Jung S, Kim M, Kwon H, Bang J (2022). Effects of Zn impurity on the photoluminescence properties of InP quantum dots. J. Lumin..

[42] Ondry JC, Philbin JP, Lostica M, Rabani E, Alivisatos AP (2019). Resilient pathways to atomic attachment of quantum dot dimers and artificial solids from faceted CdSe quantum dot building blocks. ACS Nano.

[43] Chen X, Lou Y, Samia AC, Burda C (2003). Coherency strain effects on the optical response of core/shell heteronanostructures. Nano. Lett..

[44] Pratt A (2013). Enhanced oxidation of nanoparticles through strain-mediated ionic transport. Nat. Mater..

[45] Shen X (2019). Oxidation-induced atom diffusion and surface restructuring in faceted ternary Pt–Cu–Ni nanoparticles. Chem. Mater..

[46] Yamaguchi Y (2022). Atomic diffusion of indium through threading dislocations in InGaN quantum wells. Nano. Lett..

[47] Legros M, Dehm G, Arzt E, Balk TJ (2008). Observation of giant diffusivity along dislocation cores. Science.

[48] Zhang Q (2022). Defect-mediated ripening of core-shell nanostructures. Nat. Commun..

[49] Kim S, Park S, Kim M, Jeong S (2023). Synthesis of single‐crystalline InP tetrapod nanocrystals via addition of ZnCl_2_. Bull. Korean Chem. Soc..

[50] Click SM, Rosenthal SJ (2023). Synthesis, surface chemistry, and fluorescent properties of InP quantum dots. Chem. Mater..

[51] Yeh C-W, Chen G-H, Ho S-J, Chen H-S (2019). Inhibiting the surface oxidation of low-cadmim-content ZnS:(Cd,Se) quantum dots for enhancing application reliability. ACS Appl. Nano Mater.

[52] Dümbgen KC, Zito J, Infante I, Hens Z (2021). Shape, electronic structure, and trap states in indium phosphide quantum dots. Chem. Mater..

[53] Shulenberger KE, Keller HR, Pellows LM, Brown NL, Dukovic G (2021). Photocharging of colloidal CdS nanocrystals. J. Phys. Chem. C.

[54] Nguyen AT, Jen-La Plante I, Ippen C, Ma R, Kelley DF (2021). Extremely slow trap-mediated hole relaxation in room-temperature InP/ZnSe/ZnS quantum dots. J. Phys. Chem. C..

[55] Proppe AH (2023). Highly stable and pure single-photon emission with 250 ps optical coherence times in InP colloidal quantum dots. Nat. Nanotechnol..

[56] Won YH (2019). Highly efficient and stable InP/ZnSe/ZnS quantum dot light-emitting diodes. Nature.

[57] Kopanja L, Žunić D, Lončar B, Gyergyek S, Tadić M (2016). Quantifying shapes of nanoparticles using modified circularity and ellipticity measures. Measurement.

[58] Baek, H. et al. Insights into structural defect formation in individual InP/ZnSe/ZnS quantum dots under UV oxidation. *Zenodo*. 10.5281/zenodo.10521345 (2024).10.1038/s41467-024-45944-238396037

