# Supplementary material for: Insights into structural defect formation in individual InP/ZnSe/ZnS quantum dots under UV oxidation
Source: Nat Commun. 2024 Feb 23;15:1671. doi: 10.1038/s41467-024-45944-2 (PMC10891109; doi:10.1038/s41467-024-45944-2)
Supplement: Supplementary file 1 — Supplementary Information [file 41467_2024_45944_MOESM1_ESM.pdf]

Supplementary Information for

Insights into structural defect formation in individual InP/ZnSe/ZnS quantum dots under UV oxidation

Hayeon Baek[1,2†], Sungsu Kang[1,2†], Junyoung Heo[5], Soonmi Choi[5], Ran Kim[5], Kihyun Kim[5], Nari Ahn[5], Yeo-Geon Yoon[5], Taekjoon Lee[5], Jae Bok Chang[5], Kyung Sig Lee[5], Young-Gil Park[5], Jungwon Park[1,2,3,4*]*

[1]School of Chemical and Biological Engineering, Institute of Chemical Processes, Seoul National University, Seoul 08826, Republic of Korea

[2]Center for Nanoparticle Research, Institute for Basic Science (IBS), Seoul 08826, Republic of Korea

[3]Institute of Engineering Research, College of Engineering, Seoul National University, Seoul 08826, Republic of Korea

[4]Advanced Institute of Convergence Technology, Seoul National University, Suwon 16229, Republic of Korea

[5]Samsung Display Co., Ltd., Giheung-gu, Yongin-si 17113, Gyeonggi-do, Republic of Korea

‡These authors contributed equally.

*Correspondence and requests for materials should be addressed to Y.-G.P. (younggil.park@samsung.com) or J.P. (jungwonpark@snu.ac.kr).

Supplementary Figures

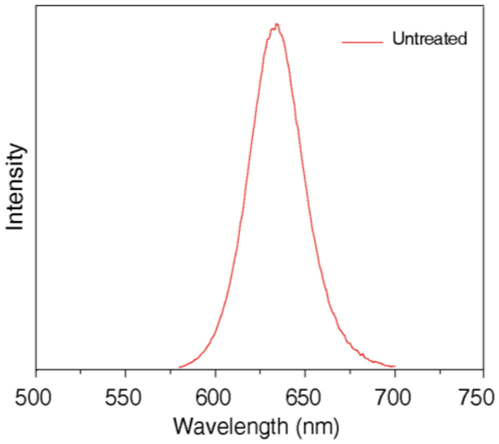

Supplementary Fig. 1 | PL emission spectrum of untreated InP/ZnSe/ZnS QDs. The wavelength at which maximum PL emission intensity is identified is located at 627 nm.

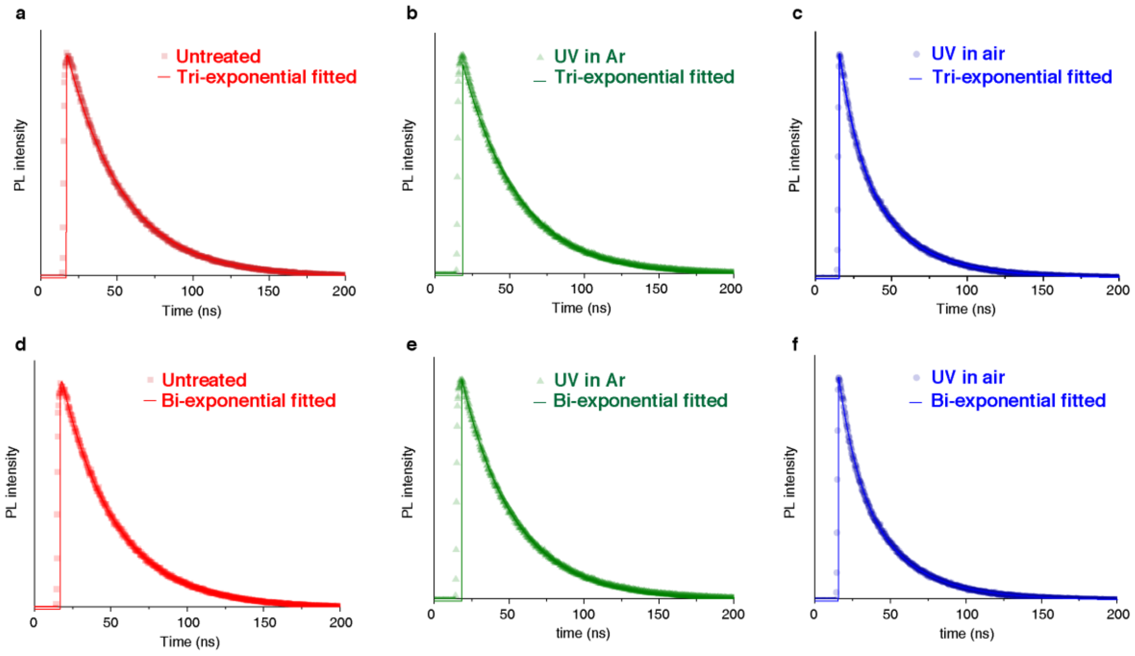

Supplementary Fig. 2 | PL decay curves of untreated and UV-exposed QDs. a–c PL decay curves for a untreated QD sample (**a**), QD sample exposed to UV in Ar (**b**), and QD sample exposed to UV in air (**c**). (lines) Fits by tri-exponential function. **d–f** PL decay curves for the untreated QD sample (**d**), QD sample exposed to UV in Ar atmosphere (**e**), and QD sample exposed to UV in air (f). (lines) Fits by bi-exponential function. See also for Supplementary Table 1 and Methods for details.

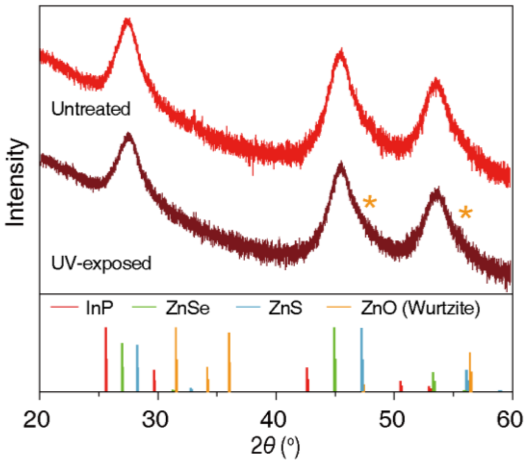

Supplementary Fig. 3 | Powder XRD of untreated and UV-exposed QDs. Major reflections are (311), (220), (111) of ZnSe with the zinc blende phase. After the UV exposure in the air, those major reflections of zinc blende ZnSe phase still remain. However, the appearance of minor additional peaks is observed, which is marked with orange stars. We found that these peaks are located in the positions with are close to the XRD peaks of wurtzite zinc oxide (ZnO) phase.

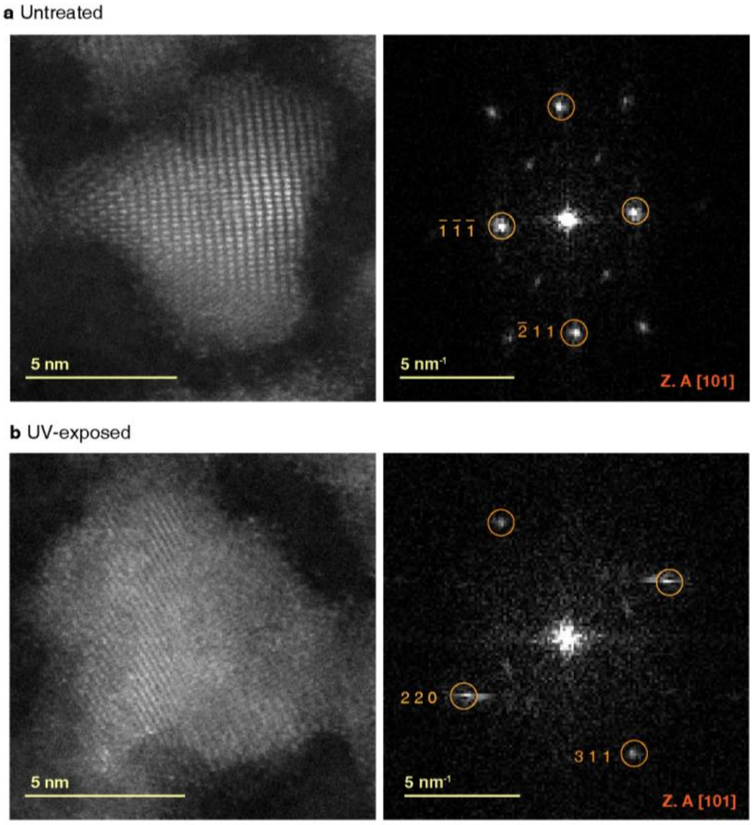

Supplementary Fig. 4 | Morphology of single QD in high magnification. a,b HAADF-STEM images (left) and corresponding FFTs (right) of a QD before (**a**) and after (**b**) the UV-facilitated oxidation.

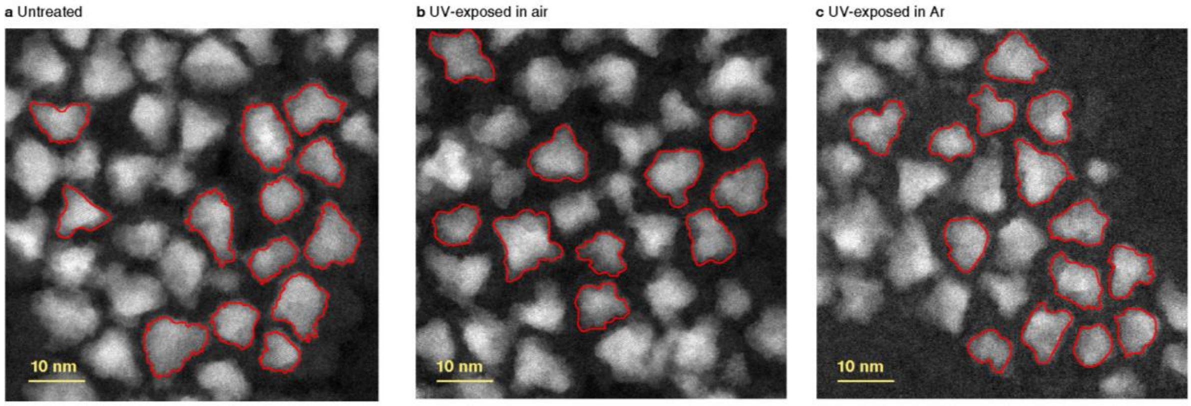

Supplementary Fig. 5 | Additional HAADF-STEM images showing morphological changes in QDs with different treatments. a–c HAADF-STEM images of QDs in untreated QDs (**a**), QDs exposed to UV in air (**b**), and QDs exposed to UV in Ar (**c**). (red lines) Contours of QDs used to measure their 2D projected areas and circularities (Methods).

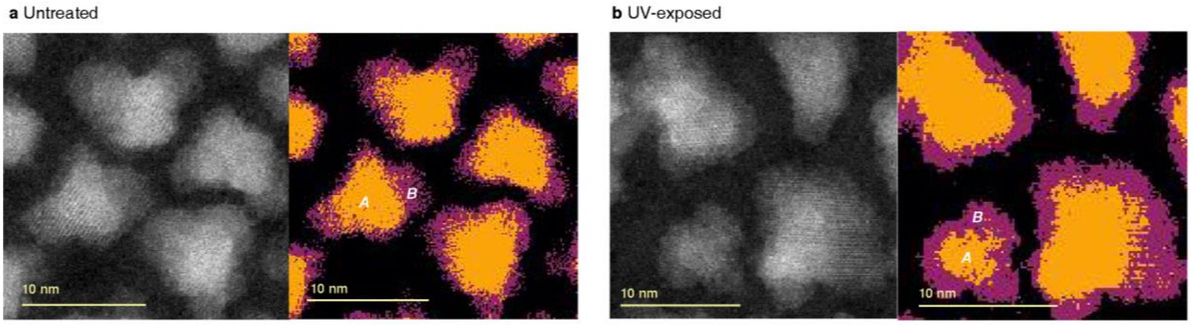

Supplementary Fig. 6 | Binarization of HAADF-STEM images to detect low-contrast and high-contrast regions in QDs. a,b HAADF-STEM images (left) and corresponding binarized images (right) of untreated QDs (**a**) and QDs after UV-facilitated oxidation (**b**). Images are binarized according to the threshold value, and categorized into high-contrast and low-contrast regions. High-contrast and low-contrast regions are denoted with *A* (orange region) and *B* (purple region), respectively.

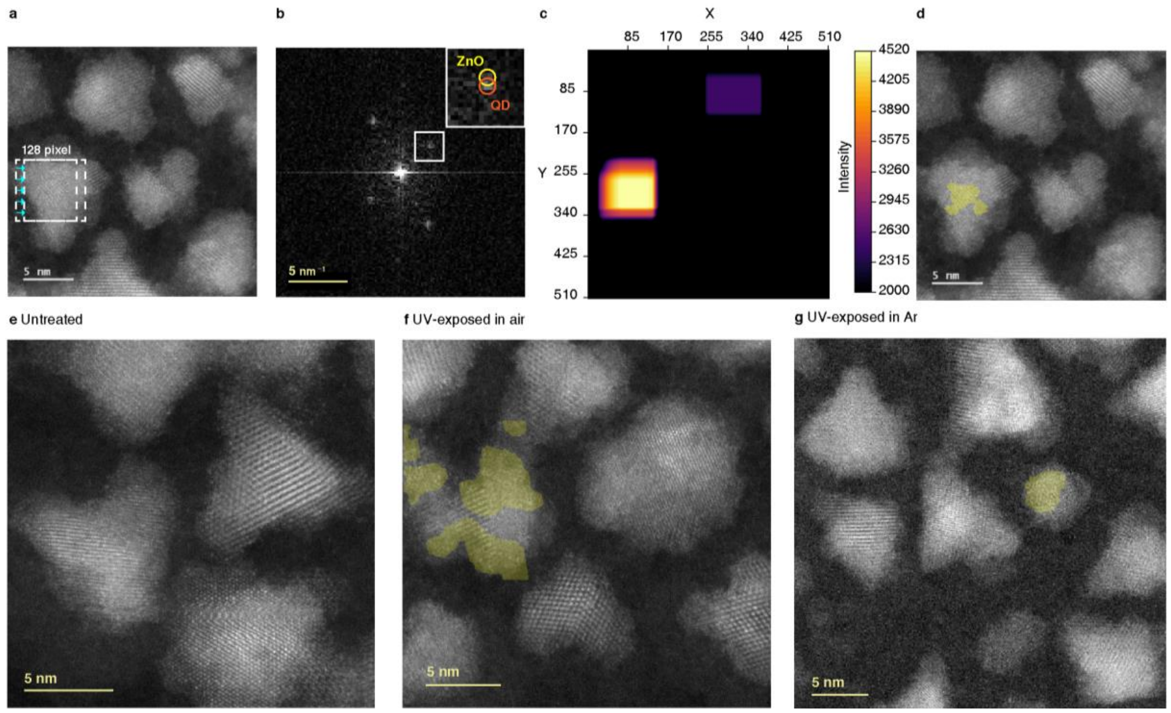

Supplementary Fig. 7 | Detection of oxide formation in QDs. a Representative HAADF-STEM image of QDs after oxidation. **b** A FFT pattern obtained from region marked with white box in (**a**). **c** The result from code operation showing rough detection of regions with ZnO. The region where ZnO domains are identified is indicated with higher pixel values. **d** An inverse FFT of selected ZnO peaks marked with the yellow circle in the inset of (**b**), overlaid on the original HAADF-STEM image (**a**). **e–g** HAADF-STEM images of untreated QDs (**e**), QDs exposed to UV in air (**f**), and QDs exposed to UV in Ar atmosphere (**g**). (yellow regions) ZnO subdomains detected with the described procedure. The details in the code operation of ZnO detection process are described in Methods.

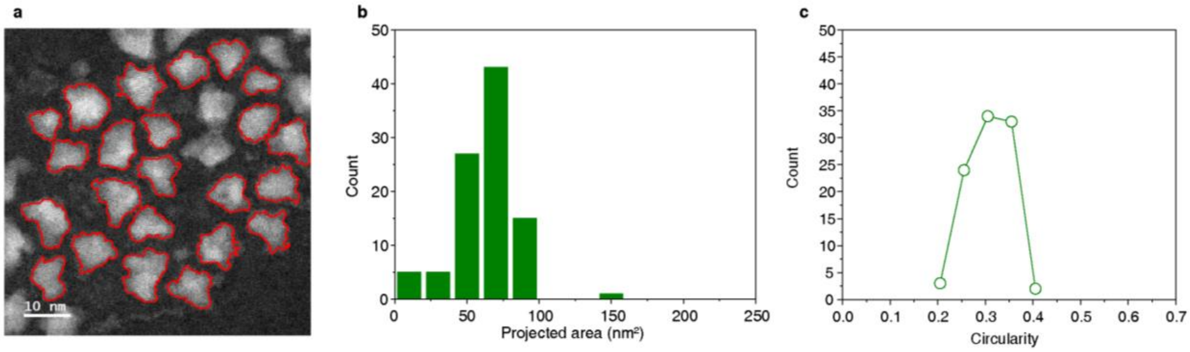

Supplementary Fig. 8 | Morphology of QDs exposed to UV light in Ar. a HAADF-STEM image of QDs exposed to UV light in Ar. (red lines) Contours of QDs used to measure their 2D projected areas and circularities. **b** Projected area distribution of the QDs. **c** Circularity distribution of the QDs. The measured 2D projected area and circularity are similar to those of the untreated QDs (Fig. 2e and f).

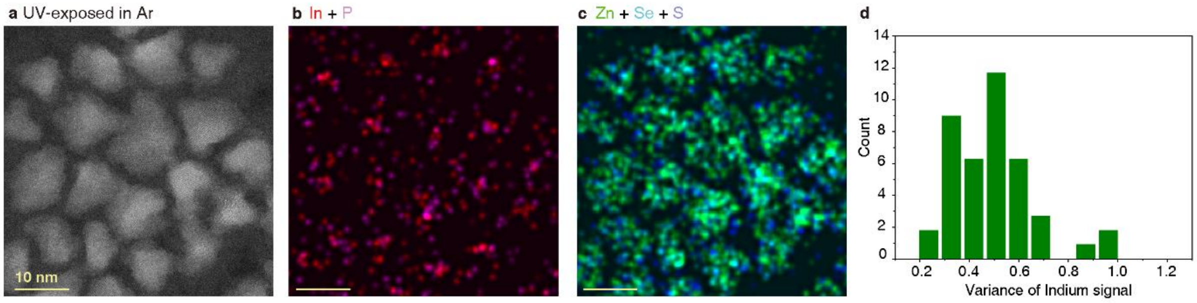

Supplementary Fig. 9 | EDS analysis of QD exposed to UV light in Ar. **a** HAADF-STEM image of QDs exposed to UV light in Ar. **b** EDS map of In (red) and P (purple). **c** EDS map of Zn (green), Se (cyan) and S (blue). All scale bars are 10 nm. **d** Variance of In EDS signals within QDs after UV exposure in Ar.

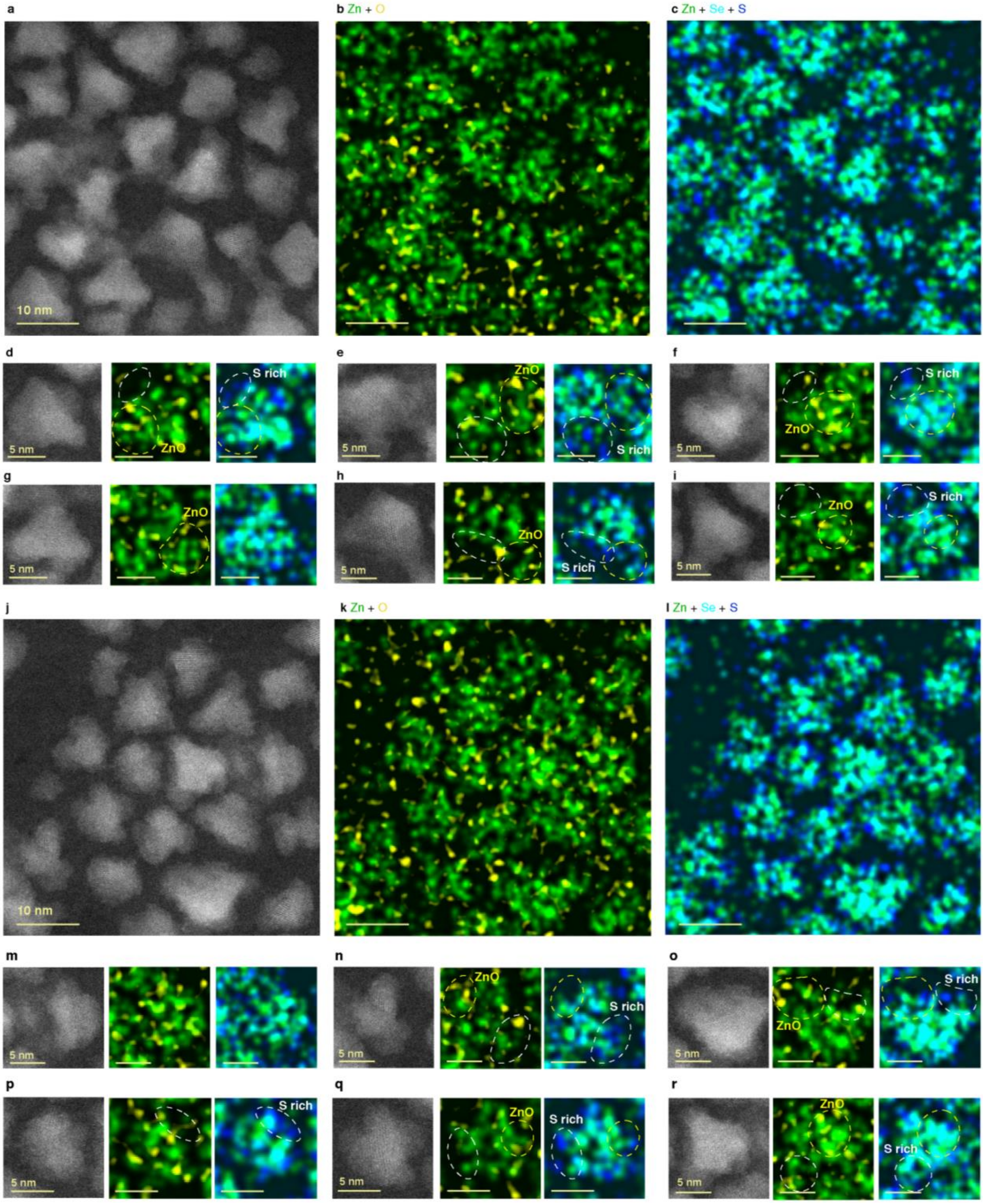

Supplementary Fig. 10 | EDS maps of ZnO and S-rich region in single QDs exposed to UV light in air. a A HAADF-STEM image of QDs after UV-facilitated oxidation. **b,c** Corresponding EDS elemental maps for Zn and O (**b**), and for Zn, Se, and S (**c**). Scale bars in a-c are 10 nm. **a–i** Magnified views of single QDs in (**a**) (left panels) with the EDS elemental

maps for Zn and O (middle panels) and for Zn, Se, and S (right panels). Scale bars in d-i are 5 nm. **j** A HAADF-STEM image of QDs after UV-facilitated oxidation. **k,l** Corresponding EDS elemental maps for Zn and O (**k**), and for Zn, Se, and S (**l**). Scale bars in j-l are 10 nm. **m–r** Magnified views of single QDs in (**k**) (left panels) with the EDS elemental maps for Zn and O (middle panels) and for Zn, Se, and S (right panels). ZnO subdomains and S-rich-regions are marked with circles in the EDS maps, and the colors in the EDS maps correspond to following elements: Zn (green), O (yellow), Se (cyan), S (blue). Scale bars in **(m)-(r)** are 5 nm.

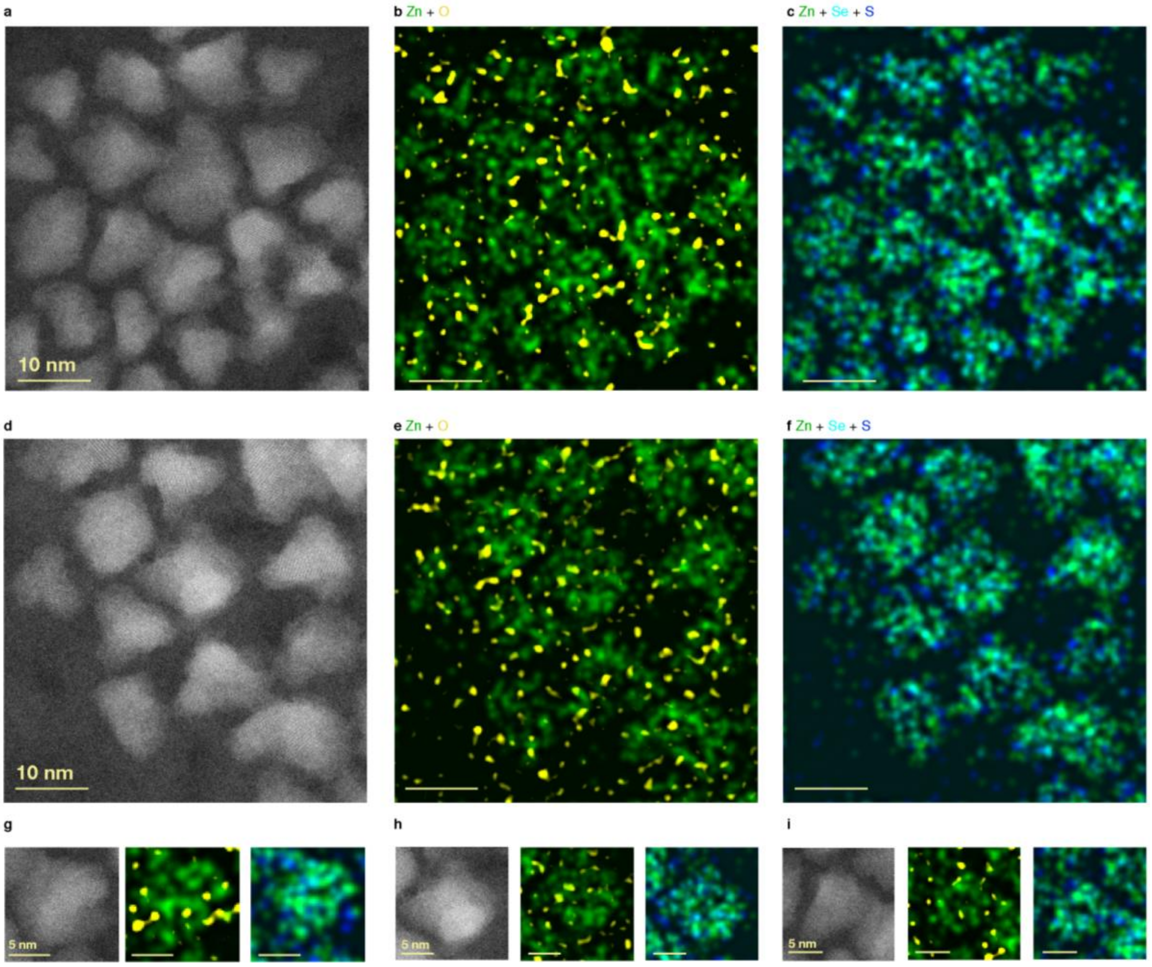

Supplementary Fig. 11 | EDS maps of ZnO and S-rich regions in single QDs exposed to UV light in Ar. a A HAADF-STEM image of QDs after UV exposure in Ar. **b,c** Corresponding EDS elemental maps for Zn and O (**b**), and for Zn, Se, and S (**c**). **d** Additional HAADF-STEM image taken at another site. **e,f** Corresponding EDS elemental maps for Zn and O (**e**), and for Zn, Se, and S (**f**). Scale bars in (**a**)–(**f**) are 10 nm. **g–i** Magnified views of single QDs in (**a**) (left panels) with the EDS elemental maps for Zn and O (middle panels) and for Zn, Se, and S (right panels). Scale bars in **(e)-(i)** are 5 nm. The colors in the EDS maps correspond to following elements: O (green), O (yellow), Se (cyan), S (blue).

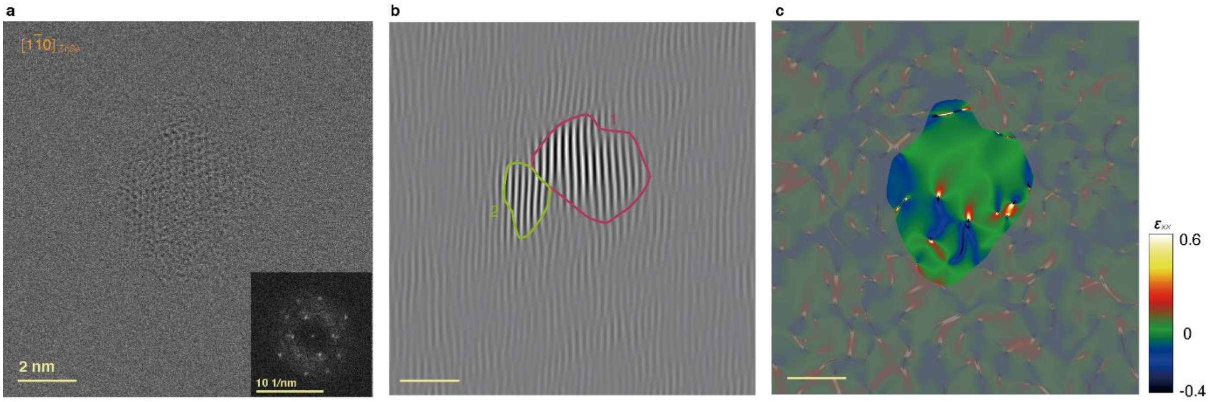

Supplementary Fig. 12 | GPA analysis of oxidized QDs. a,b High-resolution TEM image (**a**), corresponding FFT (inset), and inverse FFT (**b**) of a QD exposed to UV in air. **c** GPA strain map of the UV-exposed QD in (**a**). Scale bars in (**b)** and (**c)** are 2 nm.

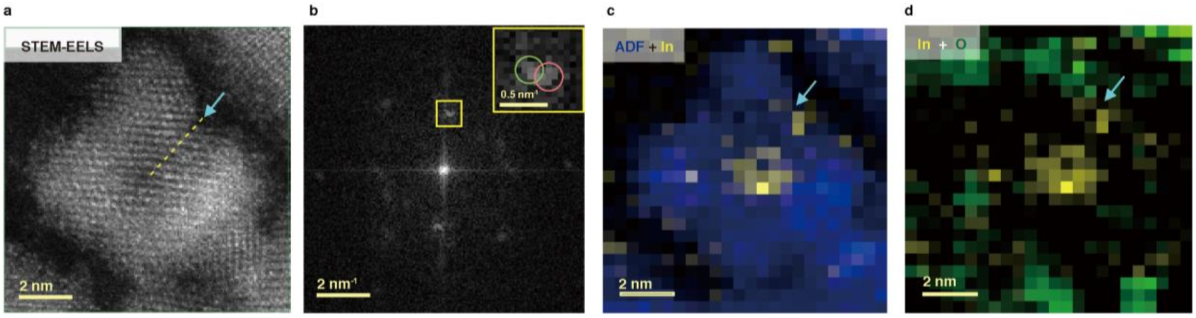

Supplementary Fig. 13 | Formation of dislocation in oxidized QD. a ADF-STEM of a QD exposed to UV in air. The dashed line marked with a yellow dashed line and light-blue colored arrow indicates a dislocation. **b** FFT of the ADF-STEM image in (**a**). Satellite peak is indicated as two circles in the inset. **c** In elemental map (yellow) and ADF-STEM intensity (blue) measured during the EELS measurement of the QD in (**a**). **d** In (yellow) and O elemental maps (green) obtained by EELS of the region in (**a**). Blue arrows indicate the dislocation observed in (**a**).

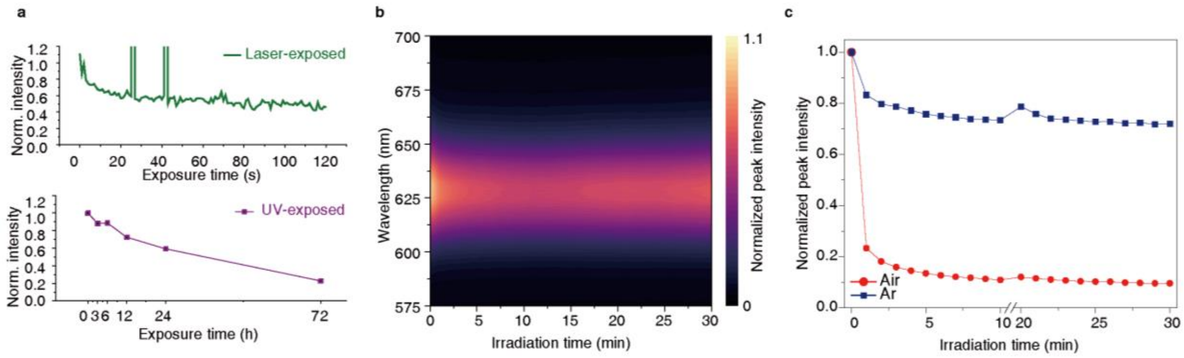

Supplementary Fig. 14 | Comparison of PL emission intensity between laser and UV light exposure to QDs and QDs after the laser exposure in Ar atmosphere. a Changes in the normalized maximum PL emission intensity over laser exposure (upper panel, green line) and UV exposure (lower panel, purple line). **b** *In situ* PL measurements of QDs during the laser exposure in an Ar atmosphere for 30 min. **c** Changes in the normalized emission peak intensity during the laser exposure in Ar (blue line) and ambient (red line) environment for 30 min.

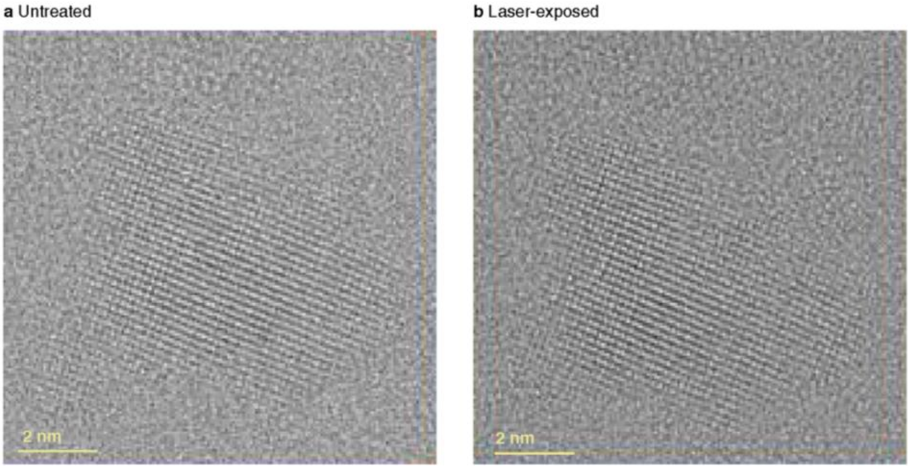

Supplementary Fig. 15 | High-resolution TEM image of identical QD. a High-resolution TEM image of a QD before laser exposure. **b** High-resolution TEM image of the same QD after laser exposure.

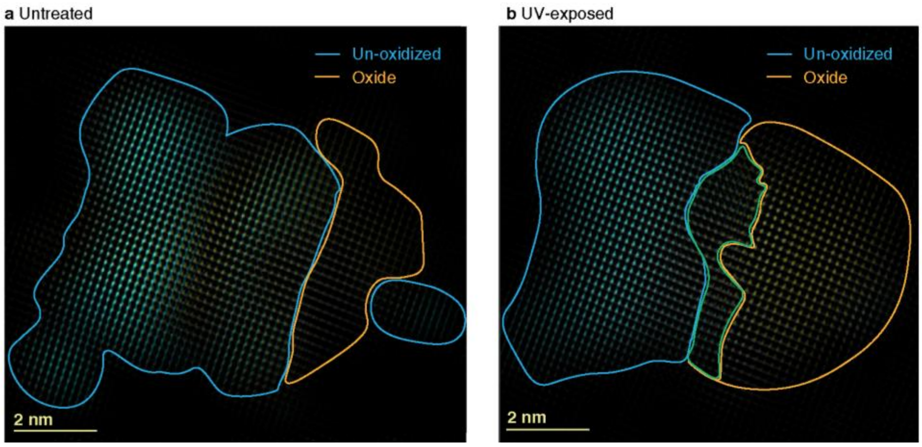

Supplementary Fig. 16 | Oxide subdomain in the identical QD. a The colored inverse FFT of untreated QD. **b** The colored inverse FFT of UV-exposed QD. Un-oxidized part is colored with blue and the oxide is colored with yellow.

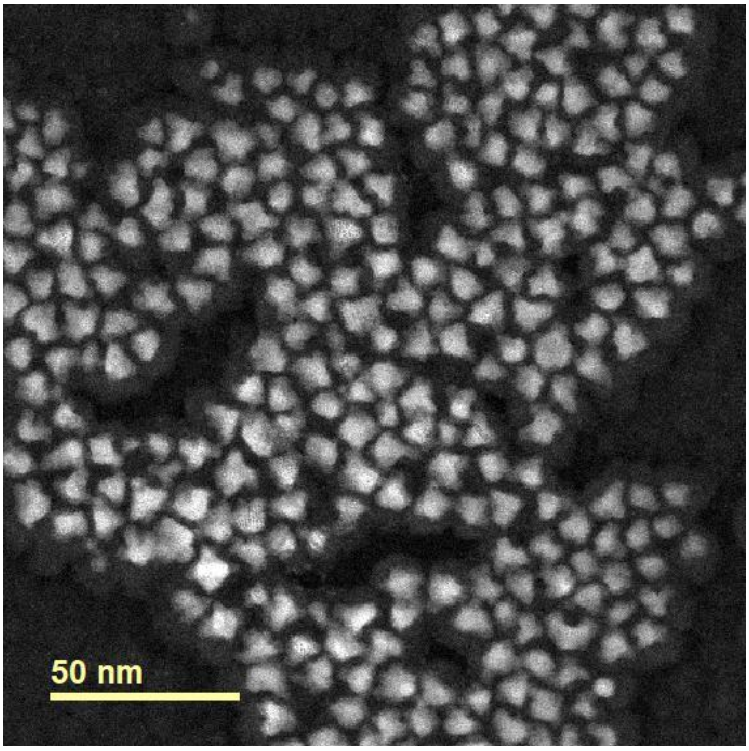

Supplementary Fig. 17 | Low-magnification HAADF-STEM images of QDs. TEM images are obtained from the diluted QD solution (concentration: 5.87×10^{-5} mg/mL). The details of dilution process are provided in the Methods.

Supplementary Table

Supplementary Table 1 | a,b Fitted pre-exponentials (*A*) and lifetimes (τ) of PL decay curves presented in Supplementary Fig. 2 by tri-exponential function (**a**) and bi-exponential function (**b**).

a

Lifetime (pre-exponential)	τ_1 (A_1)	τ_2 (A_2)	τ_3 (A_3)	χ^2
Untreated	1.59 ns (-0.16)	32.17 ns (0.35)	53.71 ns (0.084)	1.07
UV in Ar	9.38 ns (5.65E-3)	54.40 ns (0.085)	31.76 ns (0.32)	1.03
UV in air	25.39 ns (0.24)	45.03 ns (8.51E-2)	7.21 ns (0.12)	1.11

b

Lifetime (pre-exponential)	τ_1 (A_1)	τ_2 (A_2)	χ^2
Untreated	32.52 ns (0.34)	54.38 ns (7.60E-2)	1.10
UV in Ar	30.59 ns (0.30)	51.71 ns (0.11)	1.03
UV in air	14.01 ns (0.21)	36.67 ns (0.22)	1.27
