# Supplementary material for: Insights into structural defect formation in individual InP/ZnSe/ZnS quantum dots under UV oxidation
Source: Nat Commun. 2024 Feb 23;15:1671. doi: 10.1038/s41467-024-45944-2 (PMC10891109; doi:10.1038/s41467-024-45944-2)
Supplement: Supplementary file 2 — Peer Review File [file 41467_2024_45944_MOESM2_ESM.pdf]

nature portfolio

Peer Review File

Insights into structural defect formation in individual InP/ZnSe/ZnS quantum dots under UV oxidation

Open Access This file is licensed under a Creative Commons Attribution 4.0 International License, which permits use, sharing, adaptation, distribution and reproduction in any medium or format, as long as you give appropriate credit to the original author(s) and the source, provide a link to the Creative Commons license, and indicate if changes were made. In the cases where the authors are anonymous, such as is the case for the reports of anonymous peer reviewers, author attribution should be to 'Anonymous Referee' followed by a clear attribution to the source work. The images or other third party material in this file are included in the article's Creative Commons license, unless indicated otherwise in a credit line to the material. If material is not included in the article's Creative Commons license and your intended use is not permitted by statutory regulation or exceeds the permitted use, you will need to obtain permission directly from the copyright holder. To view a copy of this license, visit http://creativecommons.org/licenses/by/4.0/.

REVIEWER COMMENTS

Reviewer #1 (Remarks to the Author):

Review on NCOMMS-23-34285
This manuscript reports a state-of-the-art TEM study of InP/ZnSe/ZnS quantum dots providing an unprecedented insight into the formation of structural defects responsible for photo-induced degradation of QD photoluminescence. The TEM experiments and data are truly impressive and bring a lot of new information on the nature and propagation of defects during the UV-facilitated oxidation of QDs. The work could have profound and long-lasting impacts not only on the fundamental understanding and synthesis of QD but also on a wide range of QD applications such as QLED displays. I therefore strongly recommend the publication of this paper in Nature Communications.
I suggest authors to consider the following comments upon revision.
1. Fig. 2D: Indicate "locally rough surfaces",
2. Fig. 2f: Provide explanation on how EDS map scatterness is quantified. Did the authors measure scatterness for the UV exposure in Ar measure? If so add the data points to 2f.
3. Fig. 2g: Does none of the QDs in untreated and exposure in Ar show ZnO formation? If so state clearly. If there were some add their domain size distribution to Fig. 2g as columns of different color.
4. Can EDS scatterness of S be used in a similar way as that of In to quantify the formation of S rich region and made a plot similar to 2f?

Reviewer #2 (Remarks to the Author):

The paper "High-resolution observation of defect formation in individual photo-oxidized InP/ZnSe/ZnS quantum dots" presents the insight of structural defects in single InP/ZnSe/ZnS core/shell/shell QDs by using atomically-resolved transmission electron microscopy (TEM). However, this work focuses more on observation and drawing conclusions, while lacks in-depth analysis, which are not sufficiently influential and innovative for the publication of Nature Communications; and there are deficiencies (listed below) that should be addressed before acceptance for other publications. Some detailed comments are as follows:
1.From the characterizations obtained through STEM and EELS, the authors concluded that the oxidation of QDs is accompanied by the diffusion of In and Zn. However, the mechanism and kinetic analysis of this diffusion process were not explained. I suggest adding corresponding analysis to further explore the physical and chemical mechanisms involved in this process.
2.The methods in this manuscript illustrated that the red QD solution was diluted with toluene to 5.87×10^{-4} mg/mL. Diluting to this concentration usually requires a large amount of solvent. How did the author select a trace solution while ensuring the presence of QD in the solution before characterizing it? I think this needs to be written more clearly in the method
3.The authors claimed that the PLQY of QD solutions exposed to UV light in an Ar atmosphere decreased from 71.7% to 70%. What is the reason for this decrease? Is it still within the error range? To better understand the cause of the decrease in PLQY, more tests can be conducted to be analyzed comprehensively and provide possible explanations for the observed phenomenon.
4.This article has many formatting errors that need to be corrected, such as "thean" and "including InP- and Cd-based QDs and is frequently used…".

Reviewer #3 (Remarks to the Author):

The present paper studies the degradation mechanism of InP/ZnSe/ZnS core/shell/shell QDs upon UV irradiation under air. In addition to Raman spectroscopy, STEM, EDS and EELS have been used on the single-particle level to identify the structural changes occurring during UV exposure in the presence or absence of air.

While these findings could be in principle of high interest for understanding the degradation mechanisms in InP-based QDs, the highly inhomogeneous character of the used QDs as visible from the TEM images (e.g., Fig. 2a,d) makes an unambiguous interpretation very delicate. There is a significant size and shape distribution already in the pristine sample without any light or air exposure. More spherical, state-of-the-art InP/ZnSe/ZnS QDs with lower size distribution have been obtained by working at higher temperature (340°C, cf. Nature 2019). Moreover, there are two strong hypotheses, which are in my opinion not fully supported by the data:
1) oxydation of the outermost ZnS shell and formation of ZnO domains (cf. comment 7 below)
2) diffusion of indium into the ZnSe shell: only Fig. 2 e/f seem to underpin this argument and this data is obtained from one single particle. The statistics on a larger number of QDs would be required to confirm this claim.

Further comments:
1) The synthesis procedure for the used QDs needs to be given, at least in SI.

2) On p.8 the authors state that the InP core diameter is 1.8 nm, which does not seem to match an emission at 630 nm.

3) The observed (although slight) red-shift of the PL after photo-oxidation is unexpected and the cited Zn diffusion into the InP lattice (p. 6) should rather lead to a blue-shift. Please comment.

4) The accuracy of PLQY measurements is overestimated, no digit after the comma should be given, and the error indicated.

5) Sup. Inf. Fig. 5: what do the authors mean with « clean surfaces »? The surfaces of the tetrahedral particles are for sure rough and not smooth, which is clearly visible in the images and has been demonstrated in other studies.

6) The PL decay curves and fits should be given in SI, together with the individual lifetimes and not just average lifetime. Two- and three-exponential fits should be compared.

7) In the comparison of Fig. SI 5 and 6, both of them exhibit small spherical particles on top of the large-sized tetrahedra. Where do they come from in the case of the sample kept under Ar?

8) The manuscript should be carefully proof-read to improve the style and eliminate errors.

Responses to the reviewers' comments

We truly appreciate the reviewers' valuable comments to improve the quality of our manuscript (ID: NCOMMS-23-34285) entitled **"High-resolution observation of defects formation in individual photo-oxidized InP/ZnSe/ZnS quantum dots."** We carefully addressed the Reviewers #1, #2, and #3's comments in the revised manuscript. The modifications made in the manuscript are highlighted in blue.

○ **Reviewer #1**

General comment:

This manuscript reports a state-of-the-art TEM study of InP/ZnSe/ZnS quantum dots providing an unprecedented insight into the formation of structural defects responsible for photo-induced degradation of QD photoluminescence. The TEM experiments and data are truly impressive and bring a lot of new information on the nature and propagation of defects during the UV-facilitated oxidation of QDs. The work could have profound and long-lasting impacts not only on the fundamental understanding and synthesis of QD but also on a wide range of QD applications such as QLED displays. I therefore strongly recommend the publication of this paper in Nature Communications.

Response to the general comment: We highly appreciate the Reviewer #1's positive comments. We have made our best efforts to answer the reviewer's comments, as detailed in our point-to-point responses below. During this revision, we significantly revised the entire manuscripts to address the comments from all the reviewers and to strengthen our findings on the QD oxidation, which we believe improve the quality of the manuscript, by conducting i) advanced TEM analyses on the statistically meaningful numbers of QDs, ii) measurements of QD morphologies before and after the oxidation, and iii) suggestion of the possible oxidation mechanisms. Since these changes are made throughout the manuscript, we would like to ask the reviewer's favor to look into the manuscript as well as our point-to-point responses.

Comment #1:

Fig. 2D: Indicate "locally rough surfaces"

Response #1:

We thank the Reviewer #1 for the valuable comment. In the revised Fig. 2c which shows the tens of QDs after the UV-facilitated oxidation, we indicated the locally rough surfaces of the QDs with red lines. The locally rough surfaces can also be seen in Fig. 2d in more details, showing the higher magnification HAADF-STEM images of single QDs. Our claim that the QD surfaces become rougher can be easily noticed by comparing Fig. 2c and 2d to Fig. 2a and 2b, which show HAADF-STEM images of untreated QDs (before oxidation), and Supplementary Fig. 4. Additional HAADF-STEM images of QDs (untreated, exposed to UV light in air and in Ar) showing surface morphology are shown in Supplementary Fig. 5.

To further show the changes in the surface morphology, we have calculated the circularity, defined as the 2D projected area divided by the square of the perimeter, of QDs treated in three different conditions (untreated, UV exposure in air, UV exposure in Ar; please see revised Methods for details).

It has been shown that circularity can describe the surface roughness of the irregularly-shaped nanoparticles [*Measurements*. (2016) **92**, 252-263]. The circularity of a circle is 1.0, and that of a right triangle is 0.61. From the measurement of approximately 100 QD particles, we found that the oxidized QDs (exposed by the UV light in the air) tend to show decreased circularity due to the increased perimeters of the QDs by the roughening, as shown in Fig. 2e. In Fig. 2e, the mean circularity decreases from 0.37 to 0.30 after the 72 h of the UV-facilitated oxidation.

Original Figure (Fig. 2; page 21; in the main text)

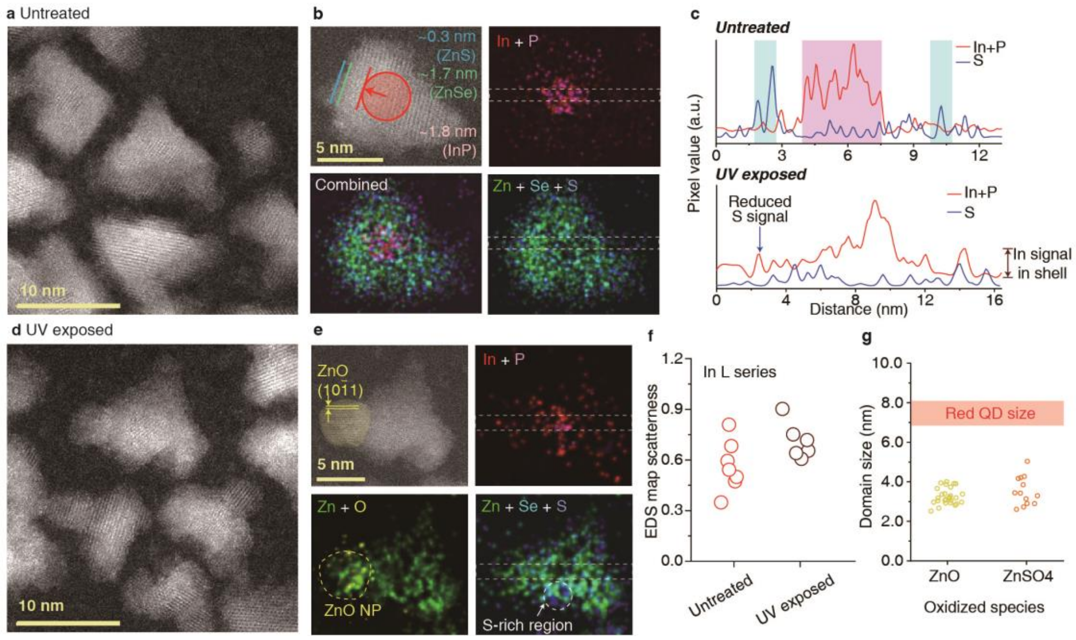

Fig. 2 | Structural changes in a single QD after photobleaching. a HAADF-STEM image of the untreated QD. **b** EDS mapping of In, P, Zn, Se, and S in the untreated. **c** Line scan from the EDS map of the untreated and UV-exposed QD. **d** HAADF-STEM image of UV-exposed QD. **e** EDS mapping of In, P, Zn, Se, and S in the UV-exposed QD. **f** Spatial distribution of indium in the untreated and UV-exposed QD. **g** Size distribution of oxidized Zn species.

Revised Figure (Fig. 2a–2e; page 26; in the main text)

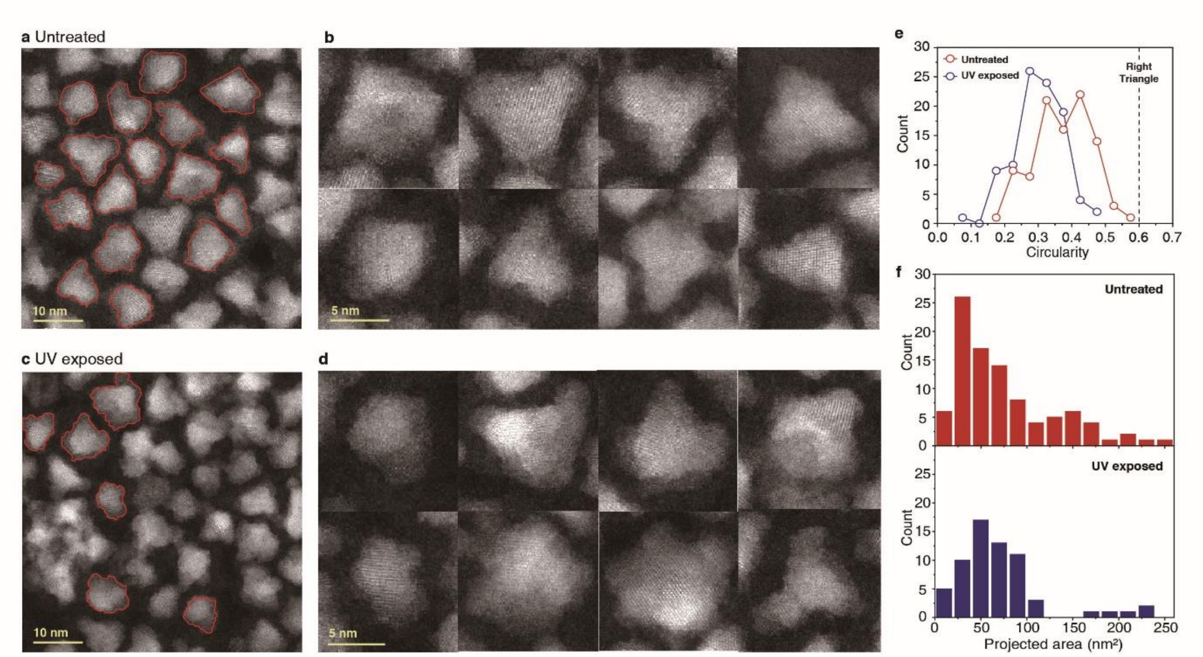

a HAADF-STEM image of the QDs before the UV-facilitated oxidation. (red line) Contours of the QDs used for the calculation of circularity. **b** Enlarged images of representative QDs before the UV-facilitated oxidation. **c** HAADF-STEM image of the QDs after the UV-facilitated oxidation for 72 h. (red line) Contours of the QDs used for the calculation of circularity. **d** Enlarged images of representative QDs after the UV-facilitated oxidation. **e** Circularity distributions of QDs before (red symbols) and after (blue symbols) the UV-facilitated oxidation. (dashed line) Circularity of a right triangle, 0.61. **f** Distributions of 2D projected area of QDs before (upper panel) and after (lower panel) the UV-facilitated oxidation.

Added Figure (Supplementary Fig. 4; page 5; in the Supplementary Information)

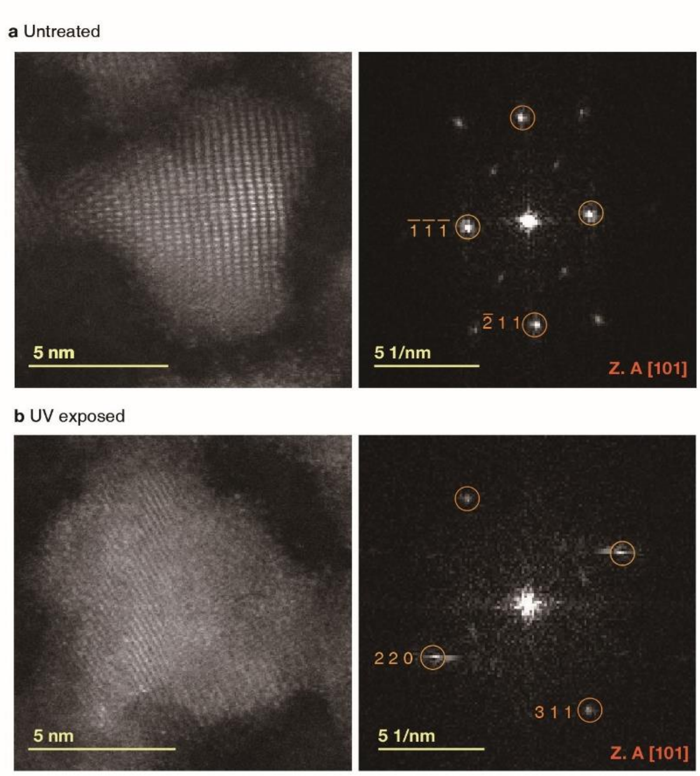

Supplementary Fig. 4 | Morphology of single QD in high magnification. a,b HAADF-STEM images (left) and corresponding FFTs (right) of a QD before (**a**) and after (**b**) the UV-facilitated oxidation.

Added Figure (Supplementary Fig. 5; page 6; in the Supplementary Information)

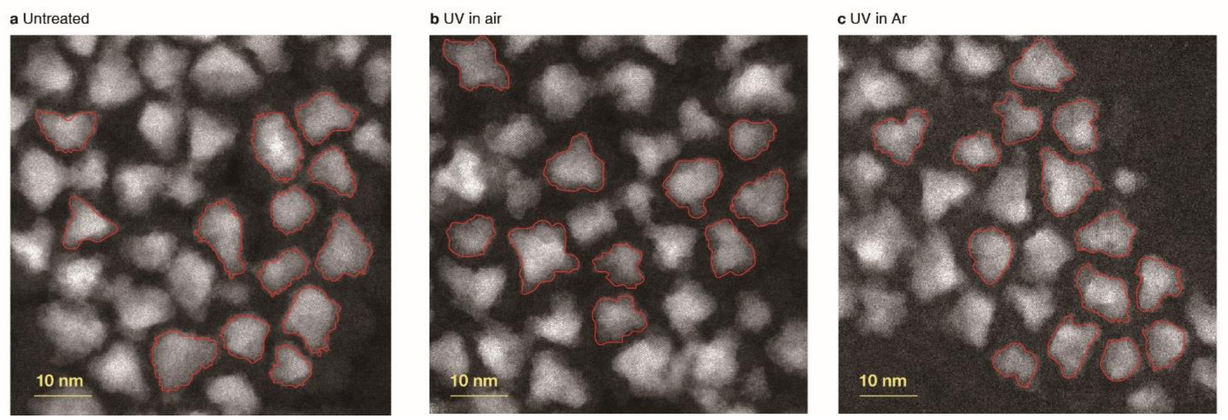

Supplementary Fig. 5 | Additional HAADF-STEM images showing morphological changes in QDs with different treatments. a–c HAADF-STEM images of QDs in untreated QDs (**a**), QDs exposed to UV in air (**b**), and QDs exposed to UV in Ar (**c**). (red lines) Contours of QDs used to measure their 2D projected areas and circularities (Methods).

Added text (Methods; page 18; in the main text)

Calculation of circularity

Circularity can be used to describe the surface roughness of the irregularly-shaped nanoparticles[52]. To investigate the surface roughness of the QDs before and after the UV-facilitated oxidation, we calculated the circularity of the QDs using the HAADF-STEM images. First, the images were binarized. Then, the contours of the QDs were identified from the binarized images, which enabled the calculation of the perimeters and 2D projected area of the QDs. The circularity, C, is calculated based on the equation below.

$$C = \frac{A}{D^2}$$

where A indicates the 2D projected area of a particle and D indicates the perimeter of the same particle. In HAADF-STEM images of the QDs, aggregated and vertically-overlapped QD particles, presumably formed during TEM sample preparation processes, were often found. We excluded those QD particles from the circularity calculation for accurate measurement, and only the isolated QD particles (marked in red contours in Fig. 2a and c) were used for the calculation. 96, 90, and 97 particles were measured for the pristine sample (without a treatment), sample exposed to UV in the air, and sample exposed to UV in an Ar atmosphere, respectively.

Original text (page 8; in the main text)

... High-resolution STEM images of the InP/ZnSe/ZnS QDs before and after 72 h of UV exposure are presented in Fig. 2. The untreated QDs mostly exhibit triangular shapes, projections of the tetrahedral morphology of QDs (Fig. 2a), and consist of single-crystalline domains (Supplementary Fig. 4). The EDS elemental maps show that the untreated QDs exhibit a well-defined InP/ZnSe/ZnS core/shell/shell structure. The InP core is spherical with a diameter of approximately 1.8 nm and is located at the center of the QD. The core is covered with a 1.7-nm-thick ZnSe shell, which is fully passivated by a 0.3-nm-thick ZnS shell (Fig. 2b and 2c). Some of the UV-exposed QDs exhibit a locally rough surface, but their size does not dramatically change, as confirmed by the high-angle annular dark-field (HAADF) STEM images in Fig. 2d and Supplementary Fig. 4. ...

Revised text (page 8; in the main text)

... The untreated QDs mostly exhibit shapes similar to triangles, the projections of a tetrahedral morphology (Fig. 2a and 2b), and consist of mostly single-crystalline domains, as shown in high-resolution high-angle annular dark-field (HAADF) STEM images (Fig. 2b and Supplementary Fig. 4). The UV-exposed QDs mostly exhibit more irregular shapes with rough surface and large size variations compared to the untreated QDs (Fig. 2c, 2d, and Supplementary Fig. 4). The difference in the particle morphology and surface structures is more clearly indicated from the 2D projected areas and circularities of the QDs, measured by identifying their contours marked with the red lines in Fig. 2a and 2c (Methods). In the UV-exposed QDs, the circularities measured for approximately 100 QD particles have smaller values (Fig. 2e, blue symbols) compared to those of the untreated QDs (Fig. 2e, red symbols) and a reference value for a right triangle (Fig. 2e, dashed line), due to the rough surface characteristic of the UV-exposed QDs (Supplementary Fig. 4 and Supplementary Fig. 5).

Comment #2:

Fig. 2f: Provide explanation on how EDS map scatterness is quantified. Did the authors measure scatterness for the UV exposure in Ar measure? If so add the data points to 2f.

Response #2:

The variance of In signal in an EDS map (referred to scatterness in the previous version of the manuscript) was calculated by averaging the distance-weighted In signal of a 2D EDS map from the center of mass of the In in the EDS map, as described in the revised Methods shown below. As the Reviewer mentioned, we also measured the variance of In signal of the QDs exposed to the UV light in Ar, and the result is added in Supplementary Fig. 9. The spatial distribution of In species is confined around the center of the QDs (Supplementary Fig. 9b), and the variances of In signals are similar to those of QDs before the oxidation (Supplementary Fig. 9d).

Added Figure (Supplementary Fig. 9; page 10; in the Supplementary Information)

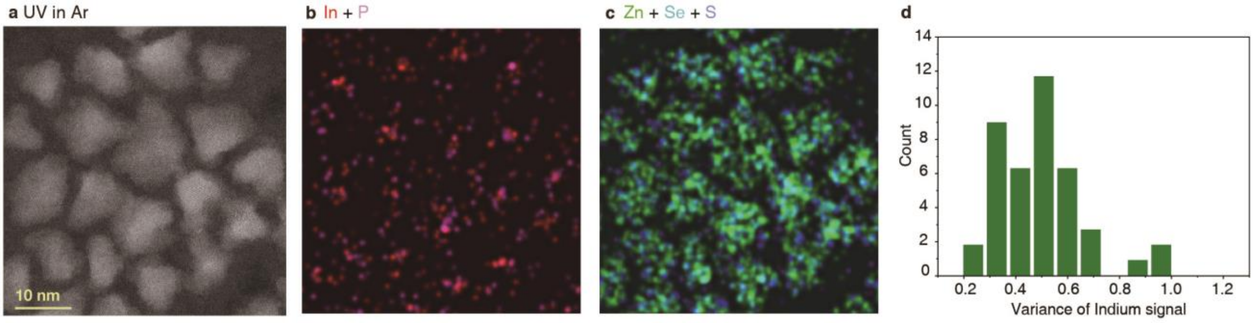

Supplementary Fig. 9 | EDS analysis of QD exposed to UV light in Ar. **a** HAADF-STEM image of QDs exposed to UV light in Ar. **b** EDS map of In (red) and P (purple). **c** EDS map of Zn (green), Se (cyan) and S (blue). **d** Variance of In EDS signals within QDs after UV exposure in Ar.

Added text (Methods; page 18–19; in the main text)

Calculation of In signal variance

The spatial distribution of In species was calculated from the EDS elemental maps for In by defining "variance of EDS signal". The variance of In signal in the EDS map is an average of the signal intensities weighted by the distance of each signal from the center of mass of the In signal in a 2D EDS map, followed by a normalization. It has a value of 0 if the In signal is detected only at a single pixel in a 2D EDS elemental map, and has a value of 1 if the In signal is uniformly distributed over a 2D map. To calculate the signal variance of In species over a single QD, we first cropped a 2D EDS elemental map for In to include a single QD and then, calculated the locations (x_{center}, y_{center}) of center of mass of the In signals in the cropped image. The distance-weighted signal average is calculated by multiplying a matrix describing the distance from a pixel at (x_i, y_j) to the center of mass by the 2D EDS elemental map for In:

$$S = \frac{1}{N}\sum_{i,j}\sqrt{(x_i - x_{center})^2 + (y_j - y_{center})^2} \cdot I$$

where I indicates a 2D EDS elemental map for In, and N indicates the total number of pixels in the map. Finally, S is divided by the S_{ref}, which is calculated for a virtual EDS map composed of uniform signals in the same way.

Added text (page 10; in the main text)

...On the other hand, the In remains in the core region after UV exposure in an Ar atmosphere, suggesting that the QD oxidation is strongly associated with the diffusion of In species (Supplementary Fig. 9). ...

Comment #3:

Fig. 2g: Does none of the QDs in untreated and exposure in Ar show ZnO formation? If so state clearly. If there were some add their domain size distribution to Fig. 2g as columns of different color.

Response #3:

Thank you for the comment. To understand whether the UV exposure induces the formation of ZnO nanoparticles in the QD samples, we have calculated the ratio of the ZnO formed particles (Fig. 2n). The ZnO nanoparticles were identified in the HAADF-STEM images by comparing the observed lattice spacings of the QDs with the known interplanar distances of wurtzite ZnO in the fast Fourier transforms of the images by a home-written script in MATLAB. The detailed workflow is shown in the Supplementary Fig. 7 and Methods. As a result, we found that the fractions of the QD particles with ZnO domains increased from 0.13 in the untreated QD sample to 0.30 in the sample exposed to the UV in the air. On the other hand, the fraction of ZnO-formed QD particles in the sample exposed to the UV in an Ar atmosphere is 0.20, less than the QDs exposed to the UV light in the air. These results are added as a Fig. 2n and corresponding main text, shown below. The small numbers of ZnO particles in the untreated QD sample is attributed to the unavoidable contact with the air during the TEM sample preparation, although we had stored the QD samples in a glove box with a O_2 concentration less than 0.5 ppm.

Added Figure (Fig. 2n; page 26; in the main text)

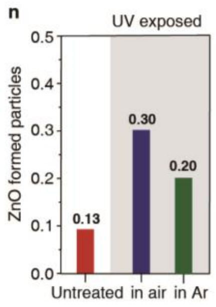

n Ratio of oxidized particles in untreated QDs (red column), QDs exposed to UV in air (blue column), and QD exposed to UV in Ar atmosphere (green column).

Added Figure (Supplementary Fig. 7; page 8; in the Supplementary Information)

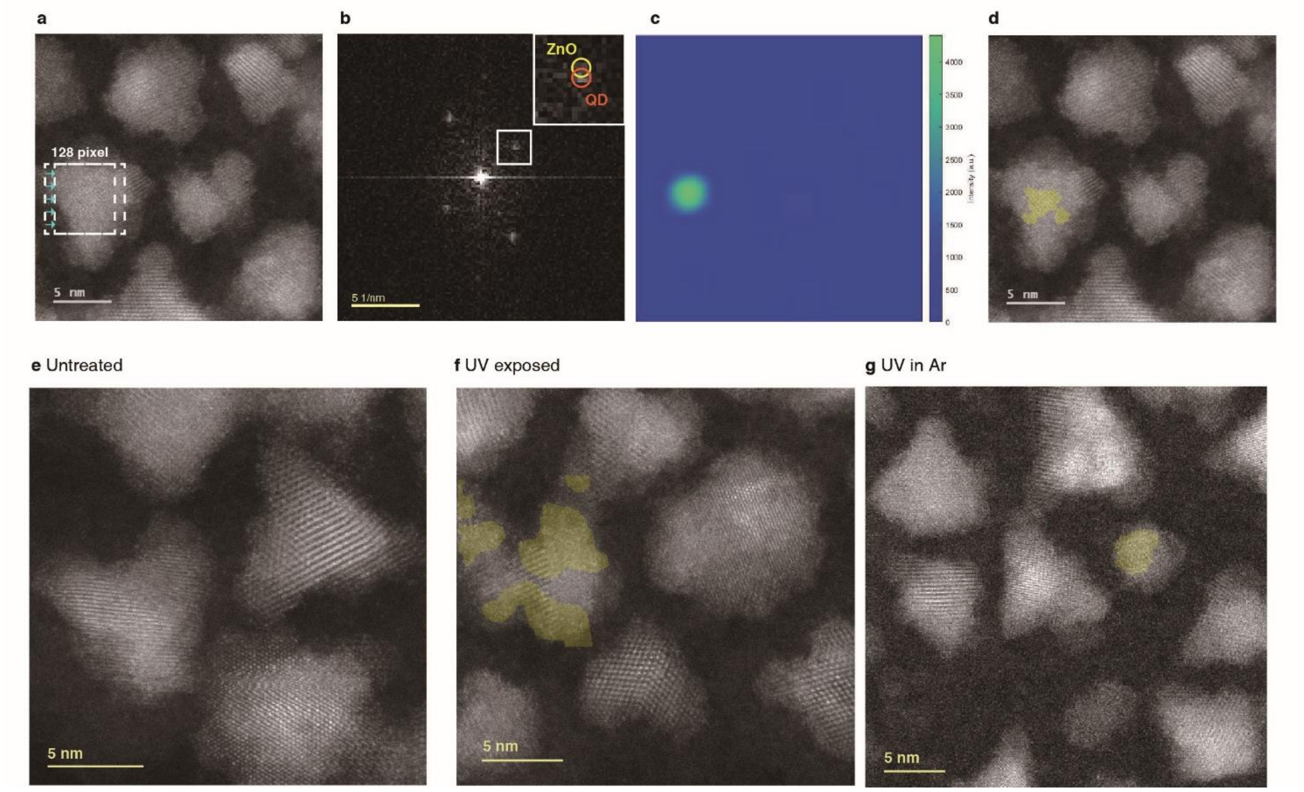

Supplementary Fig. 7 | Detection of oxide formation in QDs. a Representative HAADF-STEM image of QDs after oxidation. **b** A FFT pattern obtained from region marked with white box in (**a**). **c** The result from code operation showing rough detection of regions with ZnO. The region where ZnO domains are identified is indicated with higher pixel values. **d** An inverse FFT of selected ZnO peaks marked with the yellow circle in the inset of (**b**), overlaid on the original HAADF-STEM image (**a**). **e**–**g** HAADF-STEM images of untreated QDs (**e**), QDs exposed to UV in air (**f**), and QDs exposed to UV in Ar atmosphere (**g**). (yellow regions) ZnO subdomains detected with the described procedure. The details in the code operation of ZnO detection process are described in Methods.

Added text (Methods; page 19–20; in the main text)

Detection of ZnO subdomains in the QDs

The identification of ZnO subdomains within individual QDs was achieved through the HAADF-STEM images by using a custom MATLAB script. Initially, a small segment of the image (typically located at upper-left corner at first) was selected. FFT was then performed on this segment (Supplementary Fig. 7a). The size of the segment and HAADF-STEM images were typically 128 pixel × 128 pixel and 512 pixel × 512 pixel, respectively. From the FFT of a segment, the presence of ZnO was identified (Supplementary Fig. 7b) by detecting a peak corresponding to major lattice spacings of wurtzite ZnO, such as 2.51 Å, 2.65 Å, and 2.85 Å. If crystalline peaks of ZnO were detected in the FFT, a value of "1" was added to the 'matrix'. The 'matrix' is constructed to record information about the ZnO subdomains, with the same dimensions as the STEM image. Initially, all values in the 'matrix' were zero. This procedure was repeated by sweeping through the entire STEM image, moving 1 pixel from left to right. When the segment reached the edge of the image, it moves to the next row (1 pixel below). The ZnO detection procedure ended when the segment reached the last part of the image, and the 'matrix' is visualized as a heat map. The representative results is shown in Supplementary Fig. 7c. Once the rough location of ZnO is identified, an inverse FFT is performed at the position by crystalline ZnO peaks in the FFT pattern to map the regions with ZnO lattices (Supplementary Fig. 7d).

Added text (Methods; page 9–10; in the main text)

... Furthermore, multiple images are inspected by the crystalline phase analysis to identify ZnO domains in QDs (Supplementary Fig. 7 and Methods), and the population of the QDs with ZnO on their surfaces increases after the UV exposure in air (Fig. 2n). ... Small amount of ZnO domains are identified in these QDs exposed to UV in Ar (Fig. 2n, green bar), possibly formed by a trace amount of oxygen in the solvents and is attributed to the decrease in the PLQY (Fig. 1d).

Comment #4:

Can EDS scatterness of S be used in a similar way as that of In to quantify the formation of S rich region and made a plot similar to 2f?

Response #4:

As we mentioned in the Response #2, the variance of In signals in the EDS maps is calculated based on the dispersion of EDS signal from the center of the mass in each particle. However, since ZnS are forming a shell on a QD, the S signals would inherently appear to be dispersed in the EDS maps, meaning that the use of EDS signal variance to investigate the distribution of S species may be irrelevant. Instead, we have tried to further supplement our arguments on the oxidation-induced changes in the ZnS shell structure by conducting additional STEM-EDS analysis of the QDs. The results are presented in Supplementary Fig. 10, which clearly shows the presence of S-rich regions in the oxidized QDs with ZnO nanoparticles.

Added Figure (Supplementary Fig. 10; page 11; in the Supplementary Information)

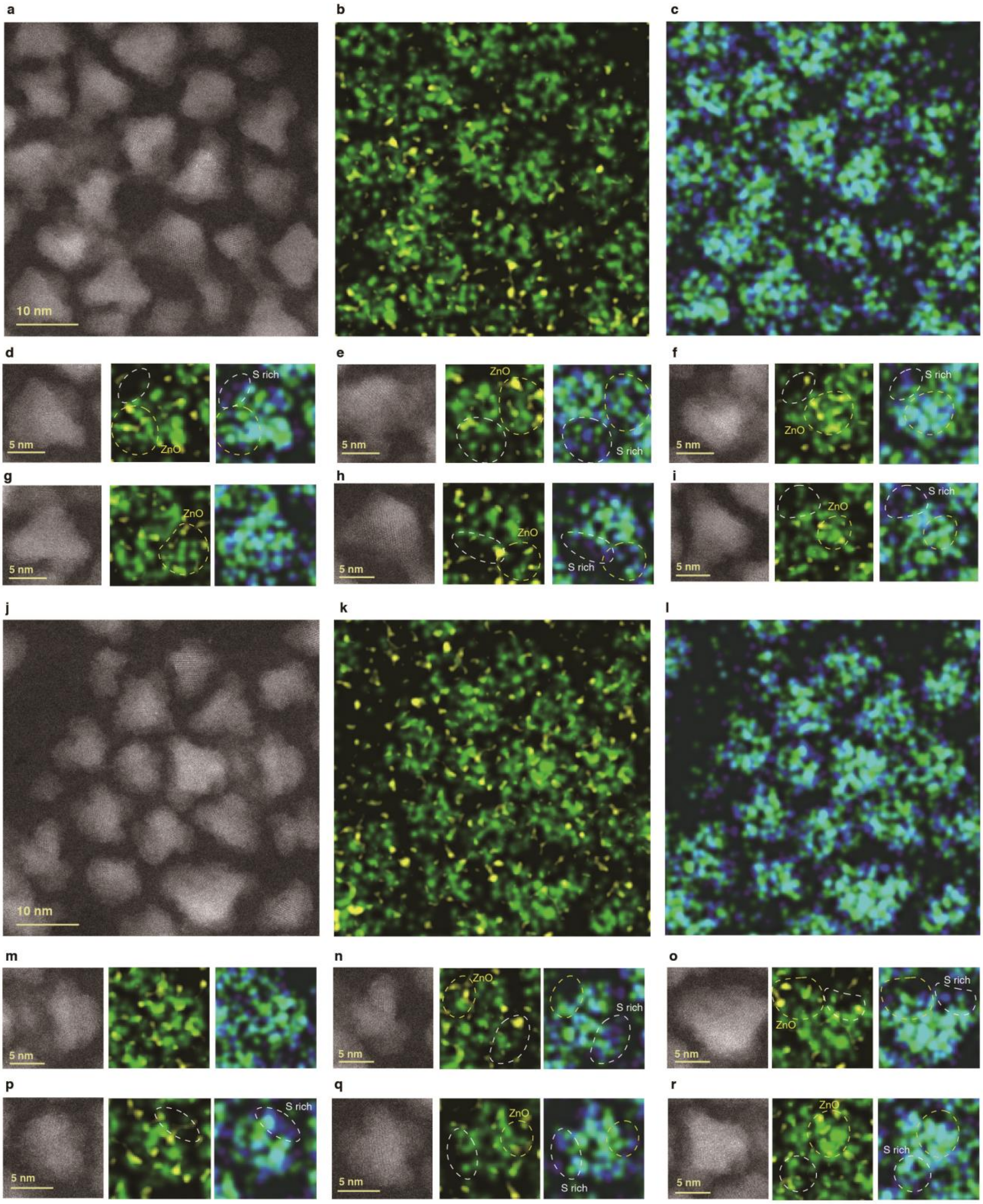

Supplementary Fig. 10 | EDS maps of ZnO and S-rich region in single QDs exposed to UV light in air. a A HAADF-STEM image of QDs after UV-facilitated oxidation. **b,c** Corresponding EDS elemental maps for Zn and O (**b**), and for Zn, Se, and S (**c**). **d–i** Magnified views of single QDs in (**a**) (left panels) with the EDS elemental maps for Zn and O (middle panels) and for Zn, Se, and S (right panels). **j** A HAADF-STEM image of QDs after UV-facilitated oxidation. **k,l** Corresponding EDS

elemental maps for Zn and O (**k**), and for Zn, Se, and S (**l**). **m–r** Magnified views of single QDs in (**k**) (left panels) with the EDS elemental maps for Zn and O (middle panels) and for Zn, Se, and S (right panels). ZnO subdomains and S-rich-regions are marked with circles in the EDS maps, and the colors in the EDS maps correspond to following elements: Zn (green), O (yellow), Se (cyan), S (blue).

Thank you very much for many appropriate and valuable comments. We are sure that these insightful comments have improved the quality of the manuscript significantly.

○ **Reviewer #2**

General comment:

The paper "High-resolution observation of defect formation in individual photo-oxidized InP/ZnSe/ZnS quantum dots" presents the insight of structural defects in single InP/ZnSe/ZnS core/shell/shell QDs by using atomically-resolved transmission electron microscopy (TEM). However, this work focuses more on observation and drawing conclusions, while lacks in-depth analysis, which are not sufficiently influential and innovative for the publication of Nature Communications; and there are deficiencies (listed below) that should be addressed before acceptance for other publications. Some detailed comments are as follows:

Response to the general comment: We highly appreciate the Reviewer #2's insightful comments. In this revision, we believe that we have improved the quality of the manuscript significantly by carrying out more in-depth and quantitative analyses and advanced TEM investigations. Specifically, we have conducted detailed structural and elemental analyses on many QDs to suggest the oxidation mechanisms of the InP/ZnSe/ZnS QDs based on statistics. Since these changes are made throughout the manuscript, we would like to ask the reviewer's favor to look into the manuscript as well as our point-to-point responses to the reviewer's comments provided below.

Comment #1:

From the characterizations obtained through STEM and EELS, the authors concluded that the oxidation of QDs is accompanied by the diffusion of In and Zn. However, the mechanism and kinetic analysis of this diffusion process were not explained. I suggest adding corresponding analysis to further explore the physical and chemical mechanisms involved in this process.

Response #1:

We are thankful for the Reviewer #2's valuable comment. The Reviewer #2 pointed out that the underlying physical and chemical mechanism of the In diffusion process in the manuscript is not described well. In the revised manuscript, we detailed the mechanism of QD oxidation with the accompanied events, such as the diffusion of In species, by providing i) structural analyses of approximately 100 QD particles based on HAADF-STEM imaging, ii) elemental distribution changes in 50 individual QDs from STEM-EDS elemental maps, and iii) phase distributions within the single QDs. Briefly, the changes in the morphology of the QDs are distinct after the UV exposure in the air, as shown in the additional HAADF-STEM images showing more QDs (revised Fig. 2a–2d, Supplementary Fig. 4 and Supplementary Fig. 5), and the circularities of QDs which were measured to support roughened surfaces after the oxidation (Fig. 2e and Methods). With these morphological changes, we found that the projected size of the QDs tend to increase after the UV-facilitated oxidation, as shown in the revised Fig. 2f. We therefore were able to hypothesize that the roughened surfaces and the increased QD sizes in the UV-exposed QDs are induced by the oxidation of the QDs. Our additional analyses based on the image contrast of the HAADF-STEM images, in which the contrast is correlated to the mean atomic number in the local region, show that the increased portion of the projected QD sizes mainly comes from the low-atomic-number materials (Fig. 2g–2h and Supplementary Fig. 6), which is identified to be ZnO from the phase analyses in the fast Fourier transforms (FFT) (Fig. 2i–2k, Supplementary Fig. 7 and Methods).

The oxide formation deforms the crystal structure of the QDs, and changes the chemical structure of the core/shell/shell QDs. We have consistently confirmed that the oxidized QDs exhibit broader distributions of In species over the QDs compared to the untreated QDs (Fig. 3a–3j). The diffusion of In might occur thorough the dislocation line with the QDs, which is derived from crystalline structure changes accompanied by the ZnO formation on the surface. The lattice parameter between ZnO and ZnS is quite different (2.85 Å for ZnO and 3.12 Å for ZnS), and dislocation can form at the ZnO-ZnS interface (Supplementary Fig. 13). It is known that dislocation can facilitate atomic diffusion in a crystal [*Nano Lett.* (2022) **22**, 6930-6935; *Science* (2008) **319**, 1646-1649; *Nat. Commun.* (2022) **13**, 2211-2220]. Also, oxide formation on nanoparticles can induce strain (Supplementary Fig. 12), and strain can facilitate atomic diffusion [*Nat. Mater.* (2014) **13**, 26-30; *Chem. Mater.* (2019) **31**, 1720-1728]. The chemical structure of the core/shell/shell QDs are modified, such as In diffusion and S distribution heterogeneity, by the crystalline defects formation after oxidation. The deformation of the chemical structure, such as alloyed interface at the core-shell [*J. Chem. Phys.* (2020) **152**, 161104-161110], oxidation of the QDs [*Science* (1998) **281**, 945-950; *ACS Omega* (2021) **6**, 6297-6304; *Chem. Mater.* (2023) **35**, 822-836; *ACS Appl. Nano Mater.* (2019) **2**, 5290-5301; *Chem. Mater.* (2021) **33**, 6885-6896], and crystalline defects [*Nano Lett.* (2018) **18**, 709-716; *Science* (1998) **281**, 945-950; *J. Am. Chem. Soc.* (2018) **140**, 15791-15803; *Nano. Lett.* (2003) **3**, 799-803; *Chem. Mater.* (2023) **35**, 822-836] can affect the luminescence properties. We have described the above descriptions for the oxidation mechanism in the revised main text and the schematic illustration for the oxidation mechanism is added as Fig. 3k.

Original Figure (Fig. 2; page 21; in the main text)

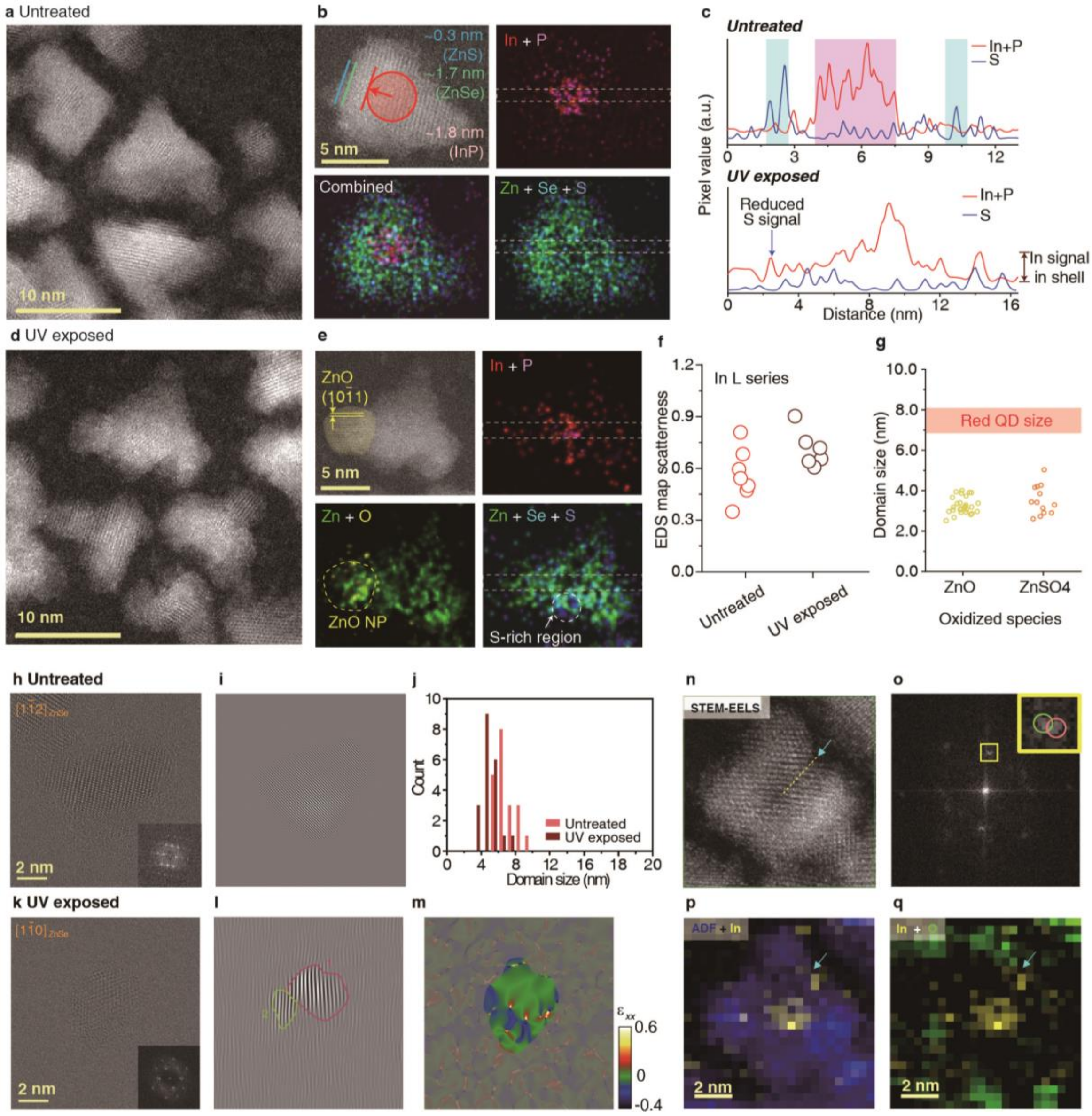

Fig. 2 | Structural changes in a single QD after photobleaching. a HAADF-STEM image of the untreated QD. **b** EDS mapping of In, P, Zn, Se, and S in the untreated. **c** Line scan from the EDS map of the untreated and UV-exposed QD. **d** HAADF-STEM image of UV-exposed QD. **e** EDS mapping of In, P, Zn, Se, and S in the UV-exposed QD. **f** Indium spatial distribution of the untreated and UV-exposed QD. **g** Size distribution of oxidized Zn species. **h,i** HRTEM image (**h**), its FFT (inset), and inverse FFT (**i**) of the untreated QD. **j** Domain size distribution of the untreated QD. **k,l** HRTEM image (**k**), its FFT (inset), and inverse FFT (**l**) of the UV-exposed QD. **m** GPA strain map of the UV-exposed QD in (**k**). **n** ADF-STEM of the UV-exposed QD. The dashed line marked with a yellow dashed line and light-blue colored arrow indicates a dislocation. **o** FFT of the ADF-STEM image in (**n**). Satellite peak is indicated as two circles in the inset. **p** In elemental map (yellow) and ADF-STEM intensity (blue) measured during the EELS measurement of the QD in (**n**). **q** In (yellow) and O elemental maps (green) obtained by EELS of the region in (**n**). Blue arrows indicate the dislocation observed in (**n**).

Revised Figure (Fig. 2; page 26; in the main text)

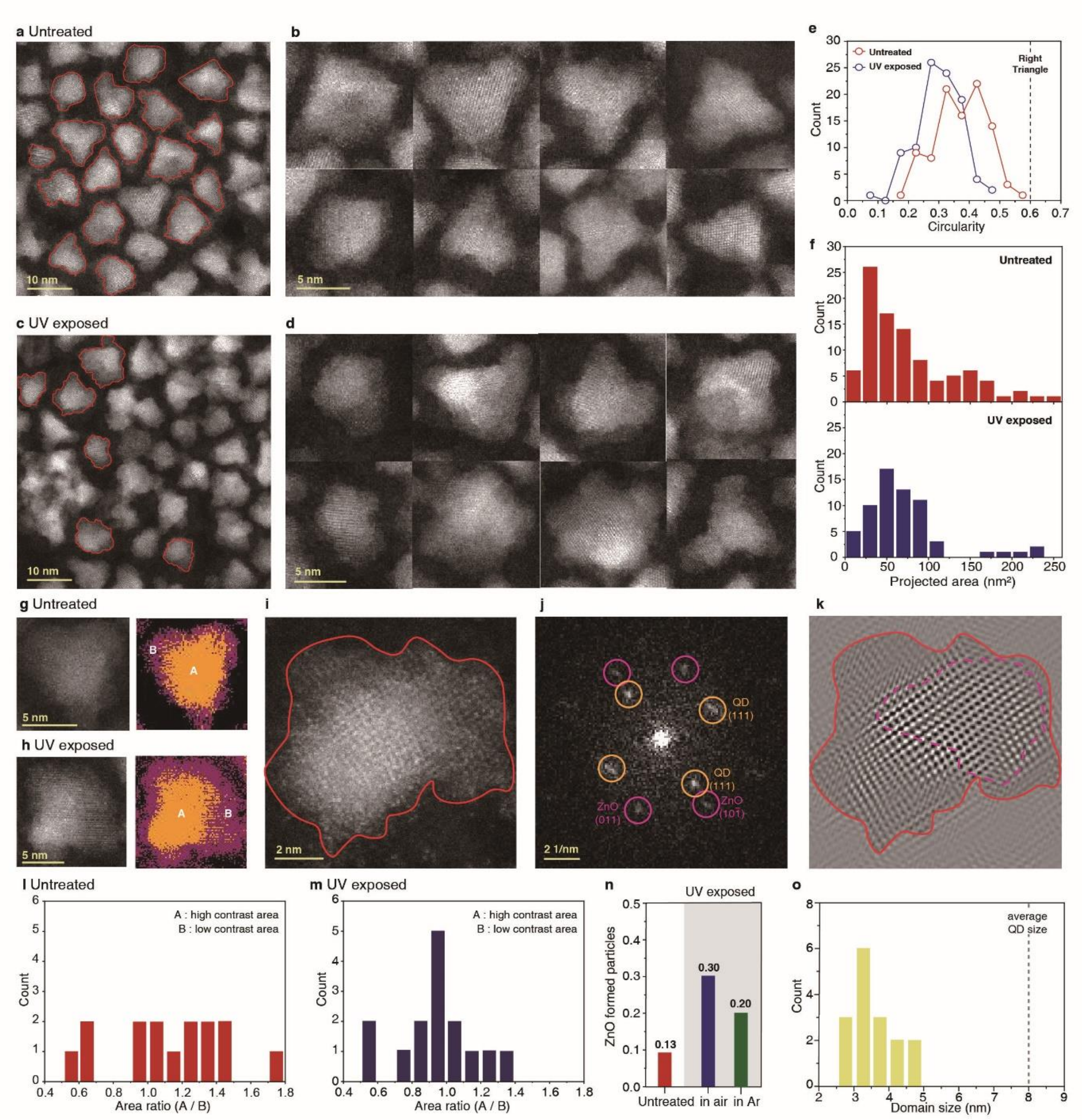

Fig. 2 | Crystal structure change of QDs by oxidation. a HAADF-STEM image of the QDs before the UV-facilitated oxidation. (red line) Contours of the QDs used for the calculation of circularity. **b** Enlarged images of representative QDs before the UV-facilitated oxidation. **c** HAADF-STEM image of the QDs after the UV-facilitated oxidation for 72 h. (red line) Contours of the QDs used for the calculation of circularity. **d** Enlarged images of representative QDs after the UV-facilitated oxidation. **e** Circularity distributions of QDs before (red symbols) and after (blue symbols) the UV-facilitated oxidation. (dashed line) Circularity of a right triangle, 0.61. **f** Distributions of 2D projected area of QDs before (upper panel) and after (lower panel) the UV-facilitated oxidation. **g,h** HAADF-STEM images (left panels) and corresponding binarized images (right panels) showing high-contrast and low-contrast regions in QDs before (**g**) and after the UV-facilitated oxidation (**h**). High-contrast and low-contrast regions are denoted with A (orange color) and B (purple color), respectively. **i–k** HAADF-STEM image of an oxidized QD taken at a higher magnification (**i**), corresponding FFT (**j**), and inverse FFT (**k**) generated from the marked peaks in (**j**). (red lines) Contours of the QD. (purple line) Detected

ZnO subdomain. **l,m** Areal ratio of high-contrast regions to low-control region of the QDs before (**l**) and after (**m**) the UV-facilitated oxidation. **n** Ratio of oxidized particles in untreated QDs (red column), QDs exposed to UV in air (blue column), and QD exposed to UV in Ar atmosphere (green column). **o** Domain size distribution of ZnO subdomains. The dashed line indicates the average size of the QDs.

Added Figure (Fig. 3; page 28; in the main text)

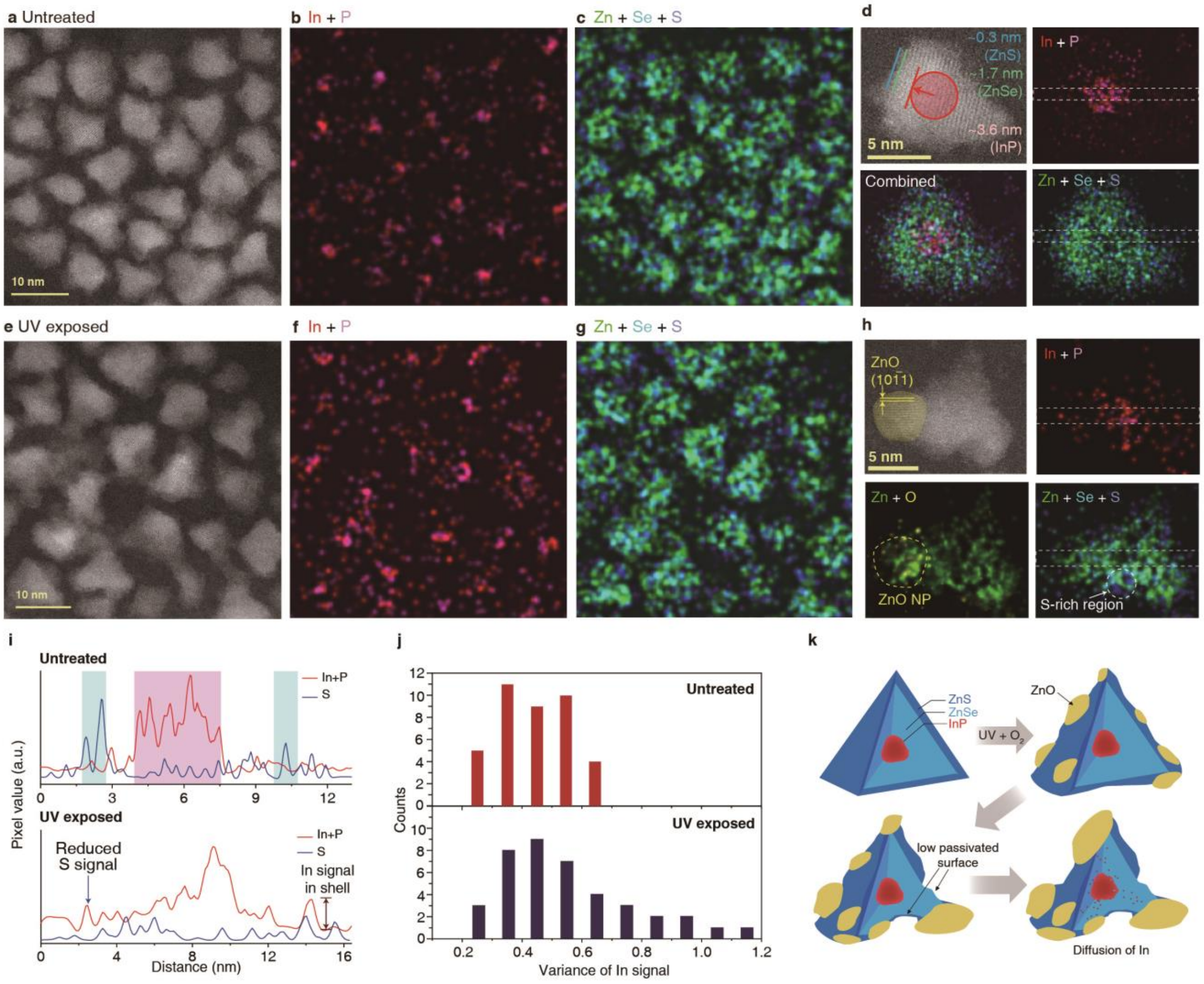

Fig. 3 | Chemical structure change of QDs by oxidation. a HAADF-STEM image of the QDs before oxidation**. b,c** Corresponding EDS elemental maps for In (red) and P (purple) (**b**) and for Zn (green), Se (cyan), and S (blue) (**c**). **d** EDS analysis of single QD before oxidation taken at a higher magnification. (upper left panel) HAADF-STEM image. (upper right panel) EDS elemental map for In and P. (lower right panel) EDS elemental map for Zn, Se, and S. (lower left panel) EDS elemental map showing all the elements. **e** HAADF-STEM image of the QDs after oxidation**. f,g** Corresponding EDS elemental maps for In (red) and P (purple) (**f**) and for Zn (green), Se (cyan), and S (blue) (**g**). **h** EDS analysis of single QD after oxidation taken at a higher magnification. (upper left panel) HAADF-STEM image. (upper right panel) EDS elemental map for In and P. (lower right panel) EDS elemental map for Zn, Se, and S. (lower left panel) EDS elemental map showing all the elements. **i** Line profiles extracted from the EDS map for In and P along the line marked in (**d**) and (**h**)**. j** Variance of In signals

in the EDS elemental maps, measured for QD before (upper panel) and after (lower panel) the UV-facilitated oxidation. **k** Schematic illustration for the proposed degradation mechanism of InP/ZnSe/ZnS QDs by the UV-facilitated oxidation**.**

Added Figure (Supplementary Fig. 5; page 6; in the supplementary Information)

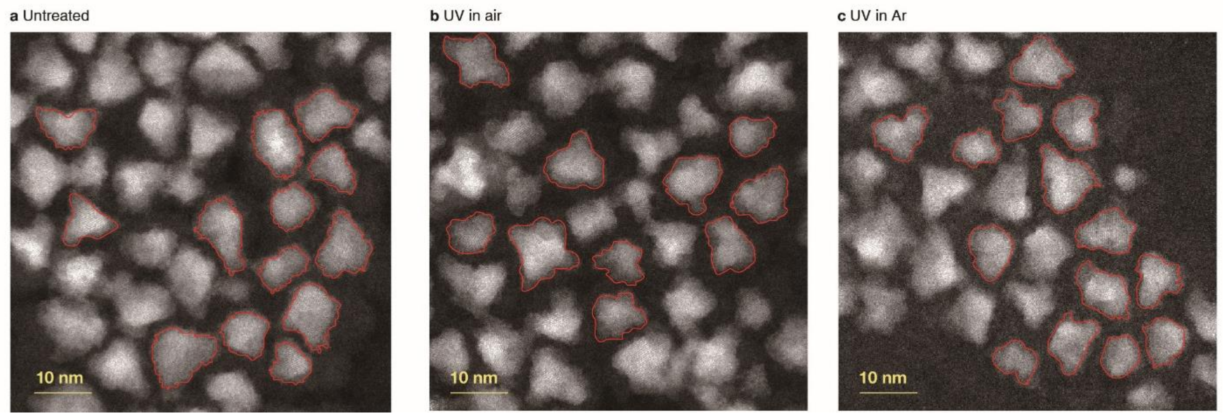

Supplementary Fig. 5 | Additional HAADF-STEM images showing morphological changes in QDs with different treatments. a–c HAADF-STEM images of QDs in untreated QDs (**a**), QDs exposed to UV in air (**b**), and QDs exposed to UV in Ar (**c**). (red lines) Contours of QDs used to measure their 2D projected areas and circularities (Methods).

Added Figure (Supplementary Fig. 6; page 7; in the supplementary Information)

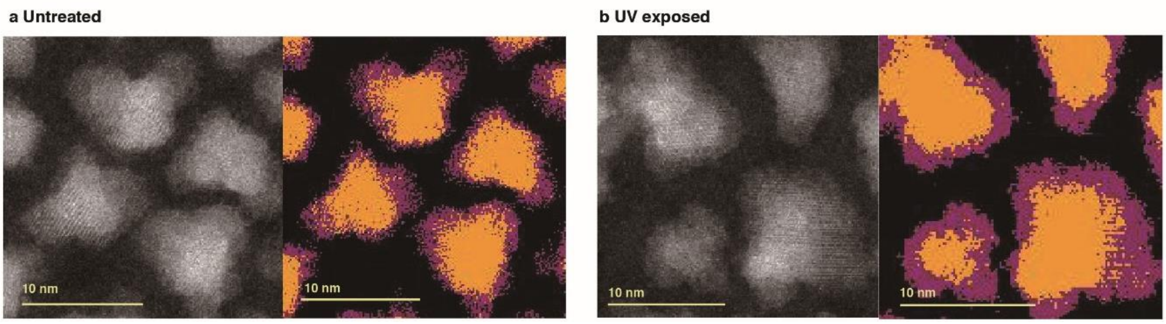

Supplementary Fig. 6 | Binarization of HAADF-STEM images to detect low-contrast and high-contrast regions in QDs. a,b HAADF-STEM images (left) and corresponding binarized images (right) of untreated QDs (**a**) and QDs after UV-facilitated oxidation (**b**). High-contrast and low-contrast regions are denoted with A (orange color) and B (purple color), respectively.

Added Figure (Supplementary Fig. 7; page 8; in the supplementary Information)

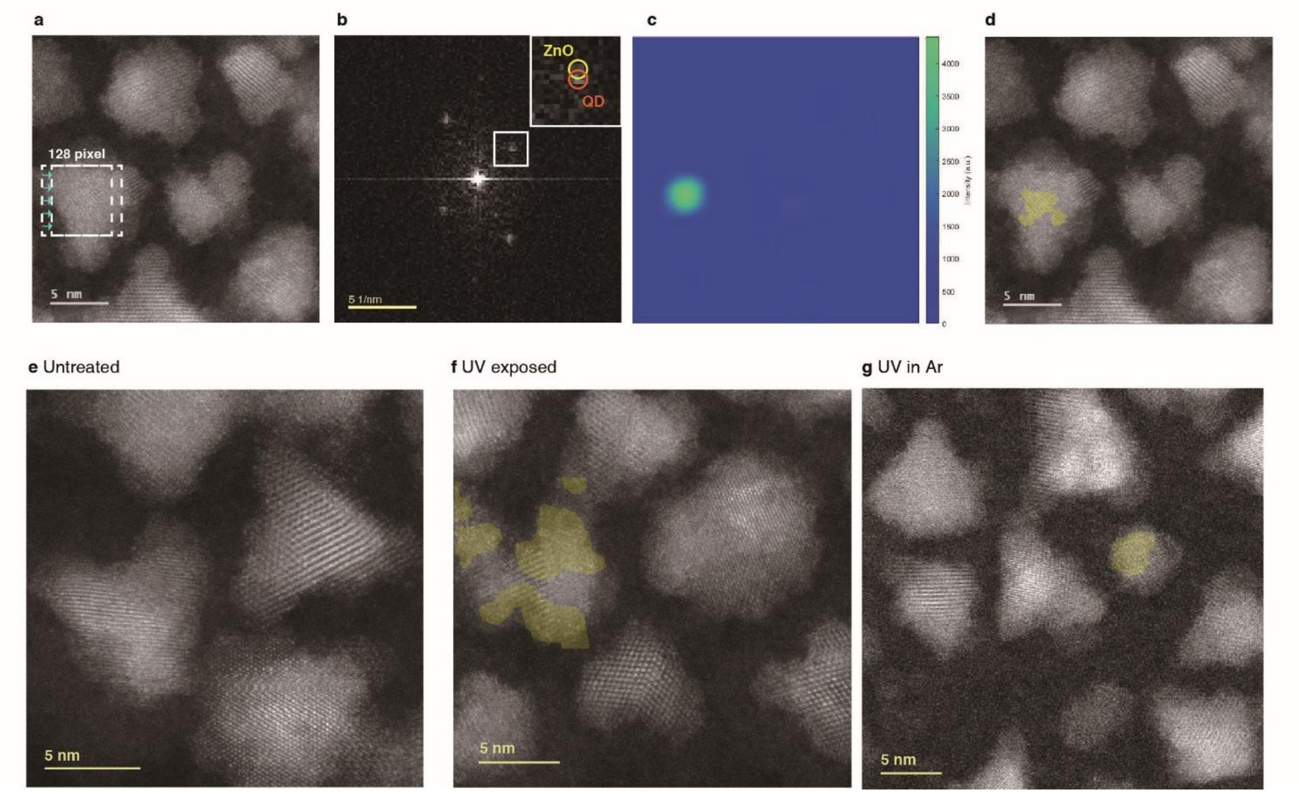

Supplementary Fig. 7 | Detection of oxide formation in QDs. a Representative HAADF-STEM image of QDs after oxidation. **b** A FFT pattern obtained from region marked with white box in (**a**). **c** The result from code operation showing rough detection of regions with ZnO. The region where ZnO domains are identified is indicated with higher pixel values. **d** An inverse FFT of selected ZnO peaks marked with the yellow circle in the inset of (**b**), overlaid on the original HAADF-STEM image (**a**). **e–g** HAADF-STEM images of untreated QDs (**e**), QDs exposed to UV in air (**f**), and QDs exposed to UV in Ar atmosphere (**g**). (yellow regions) ZnO subdomains detected with the described procedure. The details in the code operation of ZnO detection process are described in Methods.

Added Figure (Supplementary Fig. 12; page 14; in the supplementary Information)

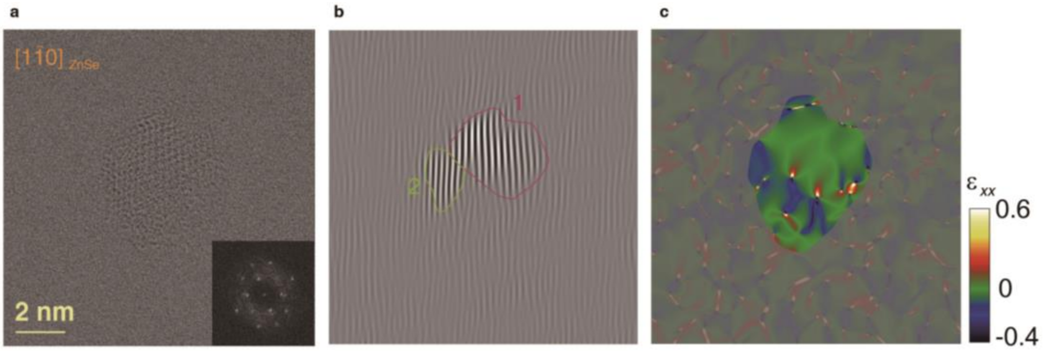

Supplementary Fig. 12 | GPA analysis of UV exposed QDs. a,b High-resolution TEM image (**a**), its FFT (inset), and inverse FFT (**b**) of the UV-exposed QD. **c** GPA strain map of the UV-exposed QD in (**a**).

Added Figure (Supplementary Fig. 13; page 15; in the supplementary Information)

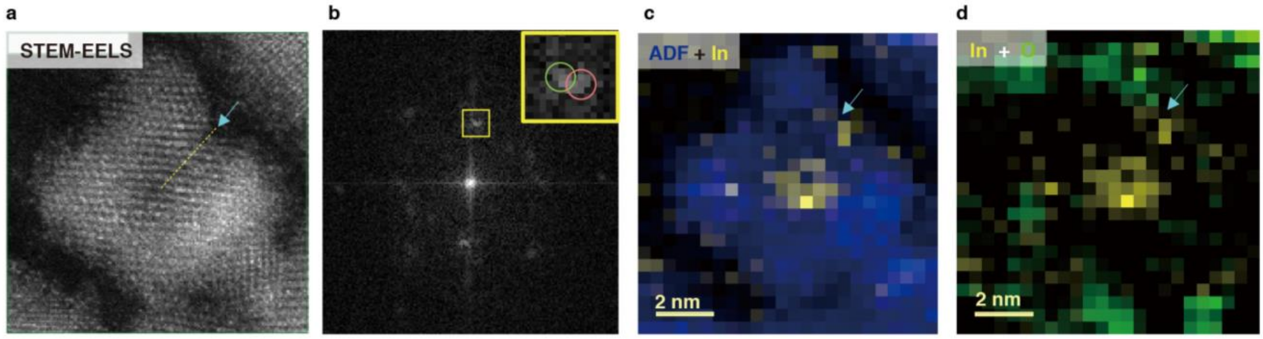

Supplementary Fig. 13 | Formation of dislocation in UV exposed QD. a ADF-STEM of the UV-exposed QD. The dashed line marked with a yellow dashed line and light-blue colored arrow indicates a dislocation. **b** FFT of the ADF-STEM image in (**a**). Satellite peak is indicated as two circles in the inset. **c** In elemental map (yellow) and ADF-STEM intensity (blue) measured during the EELS measurement of the QD in (**a**). **d** In (yellow) and O elemental maps (green) obtained by EELS of the region in (**a**). Blue arrows indicate the dislocation observed in (**a**).

Added text (Methods; page 18; in the main text)

Calculation of circularity

Circularity can be used to describe the surface roughness of the irregularly-shaped nanoparticles[52]. To investigate the surface roughness of the QDs before and after the UV-facilitated oxidation, we calculated the circularity of the QDs using the HAADF-STEM images. First, the images were binarized. Then, the contours of the QDs were identified from the binarized images, which enabled the calculation of the perimeters and 2D projected area of the QDs. The circularity, C, is calculated based on the equation below.

$$C = \frac{A}{D^2}$$

where A indicates the 2D projected area of a particle and D indicates the perimeter of the same particle. In HAADF-STEM images of the QDs, aggregated and vertically-overlapped QD particles, presumably formed during TEM sample preparation processes, were often found. We excluded those QD particles from the circularity calculation for accurate measurement, and only the isolated QD particles (marked in red contours in Fig. 2a and c) were used for the calculation. 96, 90, and 97 particles were measured for the untreated sample (without a treatment), sample exposed to UV in the air, and sample exposed to UV in an Ar atmosphere, respectively.

Added text (Methods; page 19–20; in the main text)

Detection of ZnO subdomains in the QDs

The identification of ZnO subdomains within individual QDs was achieved through the HAADF-STEM images by using a custom MATLAB script. Initially, a small segment of the image (typically located at upper-left corner at first) was selected. FFT was then performed on this segment (Supplementary Fig. 7a). The size of the segment and HAADF-STEM images were typically 128 pixel × 128 pixel and 512 pixel × 512 pixel, respectively. From the FFT of a segment, the presence of ZnO was identified (Supplementary Fig. 7b) by detecting a peak corresponding to major lattice spacings of wurtzite ZnO, such as 2.51 Å, 2.65 Å, and 2.85 Å. If crystalline peaks of ZnO were detected in the FFT, a value of "1" was added to the 'matrix'. The 'matrix' is constructed to record information about the ZnO subdomains, with the same dimensions as the STEM image. Initially, all values in the 'matrix' were zero. This procedure was repeated by sweeping through the entire STEM image, moving 1 pixel from left to right. When the segment reached the edge of the image, it moves to the next row (1 pixel below). The ZnO detection procedure ended when the segment reached the last part of the image, and the 'matrix' is visualized as a heat map. The representative results is shown in Supplementary Fig. 7c. Once the rough location of ZnO is identified, an inverse FFT is performed at the position by crystalline ZnO peaks in the FFT pattern to map the regions with ZnO lattices (Supplementary Fig. 7d).

Original text (page 8-9; in the main text)

...The untreated QDs mostly exhibit triangular shapes, projections of the tetrahedral morphology of QDs (Fig. 2a), and consist of single-crystalline domains (Supplementary Fig. 4). The EDS elemental maps show that the untreated QDs exhibit a well-defined InP/ZnSe/ZnS core/shell/shell structure. The InP core is spherical with a diameter of approximately 1.8 nm and is located at the center of the QD. The core is covered with a 1.7-nm-thick ZnSe shell, which is fully passivated by a 0.3-nm-thick ZnS shell (Fig. 2b and 2c). Some of the UV-exposed QDs exhibit a locally rough surface, but their size does not dramatically change, as confirmed by the high-angle annular dark-field (HAADF) STEM images in Fig. 2d and Supplementary Fig. 4. It is interesting to note that the elemental distribution in the EDS elemental maps becomes noticeably different after UV exposure in air. In the EDS maps, In and P are severely delocalized from the QD center (Fig. 2e). The EDS elemental maps indicate that the interface between the InP core and ZnSe shell in the oxidized QD is not as clear as that in the pristine QD, as shown in the line profiles extracted from the EDS elemental maps (Fig. 2c). These results are confirmed by comparing the extent to which the In signal is scattered over the QD domains in several pristine and oxidized QDs (Fig. 2f). In remains in the core region after UV exposure in an Ar atmosphere, suggesting that the QD oxidation is strongly associated with the diffusion of In species (Supplementary Fig. 5). Inward diffusion of Zn atoms from the ZnSe/ZnS shell can occur simultaneously with the observed In diffusion, inducing partial alloying at the core-shell interface which is known to act as a charge carrier trap[30,40].

Revised text (page 8–10; in the main text)

... The untreated QDs mostly exhibit shapes similar to triangles, the projections of a tetrahedral morphology (Fig. 2a and 2b), and consist of mostly single-crystalline domains, as shown in high-

resolution high-angle annular dark-field (HAADF) STEM images (Fig. 2b and Supplementary Fig. 4). The UV-exposed QDs mostly exhibit more irregular shapes with rough surface and large size variations compared to the untreated QDs (Fig. 2c, 2d, and Supplementary Fig. 4). The difference in the particle morphology and surface structures is more clearly indicated from the 2D projected areas and circularities of the QDs, measured by identifying their contours marked with the red lines in Fig. 2a and 2c (Methods). In the UV-exposed QDs, the circularities measured for approximately 100 QD particles have smaller values (Fig. 2e, blue symbols) compared to those of the untreated QDs (Fig. 2e, red symbols) and a reference value for a right triangle (Fig. 2e, dashed line), due to the rough surface characteristic of the UV-exposed QDs (Supplementary Fig. 4 and Supplementary Fig. 5).

QDs also exhibit the changes in their sizes as well as the morphological changes as a result of the oxidation. The 2D projected areas of the untreated QDs ranges from 0 to 100 nm^2 and the area distribution becomes broader by having populations around 250 nm^2 after the UV exposure in the air, as shown in Fig. 2f. The size increase of the UV-exposed QDs is mainly due to the increased domains with low image contrast in the HAADF-STEM images (Fig. 2g and 2h). Image contrast in HAADF-STEM images is correlated with the mean atomic number of a material, allowing the identification of materials with different atomic numbers from the image. We perform binarization of HAADF-STEM images of untreated and UV-exposed QDs to identify high- and low-contrast regions, as marked with A and B, respectively, in the right panels of Fig. 2g, 2h and Supplementary Fig. 6. Binarized images show that the low-contrast area increases in the UV-exposed QDs (Fig. 2h, purple regions), compared to the high-contrast area (Fig. 2h, orange regions), indicating the formation of low-atomic-number materials on the surface of the QDs by oxidation. Indeed, crystalline phase analysis in fast Fourier transforms (FFT) reveals that ZnO domains are observed in low-contrast regions of those QDs (Fig. 2i–k) by. In the FFT of the HAADF-STEM images of the oxidized QD, the peaks correspond to wurtzite ZnO (Fig. 2j, purple circles) and ZnSe or ZnS (Fig. 2j, orange circles) can be identified, and the inverse FFT generated from those peaks reveals a ZnO domain in the QD (Fig. 2k). The surface of QDs is indicated as red line, and the ZnO domain is located at the right side of the QD with a dashed purple line in the representative QD shown in Fig. 2k. The formation of ZnO domains on the oxidized QDs is also found in multiple QDs as shown in the areal ratio of high-contrast regions to low-contrast regions (Fig. 2l and 2m). Furthermore, multiple images are inspected by the crystalline phase analysis to identify ZnO domains in QDs (Supplementary Fig. 7 and Methods), and the population of the QDs with ZnO on their surfaces increases after the UV exposure in air (Fig. 2n). The size distribution of the ZnO subdomains is shown in Fig. 2o. On the other hand, the QDs exposed to UV in an Ar atmosphere exhibit little morphological changes compared to the untreated QDs, as shown in Supplementary Fig. 5 and 8. Small amount of ZnO domains are identified in these QDs exposed to UV in Ar (Fig. 2n, green bar), possibly formed by a trace amount of oxygen in the solvents and is attributed to the decrease in the PLQY (Fig. 1d).

Revised text (page 10; in the main text)

... These results are confirmed by comparing the variance of the In signals over the EDS maps of single QDs for 50 untreated and oxidized QDs (Fig. 3j and Methods). ...

Added text (page 11; in the main text)

... Summarizing our observations, the oxidation-induced degradation process of InP/ZnSe/ZnS QDs can be described as follows (Fig. 3k): i) oxidation of a InP/ZnSe/ZnS QD induces the formation of ZnO domains on the QD surface. ii) The formation of surface ZnO domains leads to partial etching of ZnS layers to form poorly-passivated ZnSe layers. iii) The ZnO domains also deform the lattice structure of the QD and form dislocation within the QD, which serves as a path for the diffusion of In species.

Comment #2:

The methods in this manuscript illustrated that the red QD solution was diluted with toluene to 5.87×10−4 mg/mL. Diluting to this concentration usually requires a large amount of solvent. How did the author select a trace solution while ensuring the presence of QD in the solution before characterizing it? I think this needs to be written more clearly in the method

Response #2:

Thank you for the careful comment on the experiment. As Reviewer #2 mentioned, we added explanation of the dilution process of the QD solution we used for the structure analysis. The brief description is as follows: (i) dilution starts from the QD stock solution which was 30 wt% (=1.76×10^{-1} mg/mL) in toluene. (ii) We diluted the solution to 5.87×10^{-3} mg/mL with toluene. (iii) Then we diluted again with toluene to 5.87×10^{-4} mg/mL. We have updated detailed dilution process on the "Methods" section, in main text. The QD stock solution is highly concentrated, therefore large number of QDs are observable even after the dilution (Supplementary Fig. 17).

Added text (Methods; page 15; in the main text)

The red QD solution was diluted from a red QD stock solution (30 wt% or 1.76×10^{-1} mg/mL) with toluene to 5.87×10^{-4} mg/mL for UV irradiation experiments. We first diluted stock solution into 5.87×10^{-3} mg/mL and then re-diluted with toluene to 5.87×10^{-4} mg/mL. The QD stock solution is highly concentrated, therefore large number of QDs are observable even after the dilution within field of views of (S)TEM used in this study (Supplementary Fig. 17). ...

Added Figure (Supplementary Fig. 17; page 19; in the Supplementary Information)

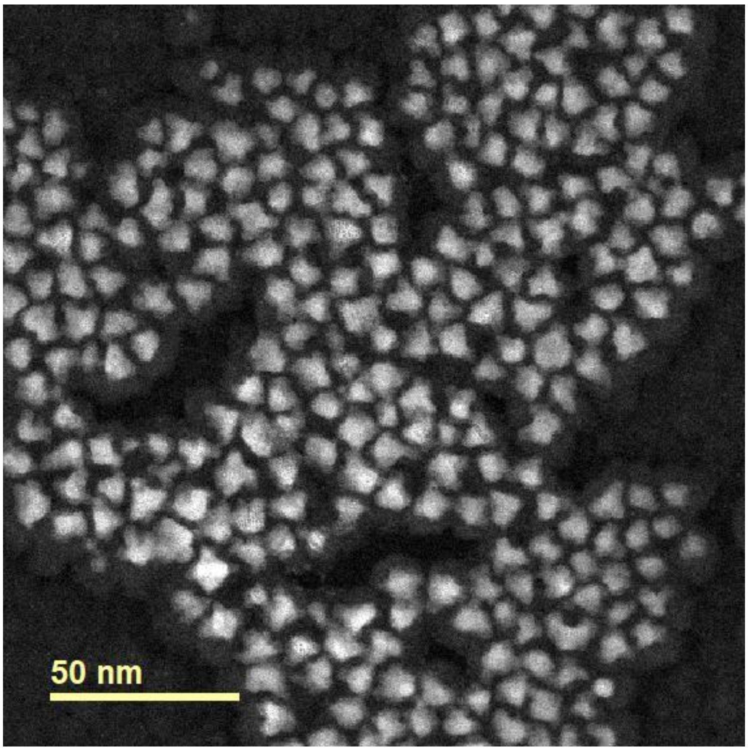

Supplementary Fig. 17 | Low-magnification HAADF-STEM images of QDs. TEM images are obtained from the diluted QD solution (concentration: 5.87×10^{-5} mg/mL). The details of dilution process are provided in the Methods.

Comment #3:

The authors claimed that the PLQY of QD solutions exposed to UV light in an Ar atmosphere decreased from 71.7% to 70%. What is the reason for this decrease? Is it still within the error range? To better understand the cause of the decrease in PLQY, more tests can be conducted to be analyzed comprehensively and provide possible explanations for the observed phenomenon.

Response #3:

We appreciate the Reviewer #2's for the valuable comment. Reviewer #2 raised a concern about the reason for the PLQY changes in the QDs exposed to the UV in an Ar environment and the accuracy of the measurements. To address this concern, we have measured the PLQY of ten samples for each circumstance (untreated, UV exposure in the air, UV exposure in the Ar), and the mean PLQYs with their standard deviations are updated to be the revised Fig. 1d. The mean PLQY of the untreated samples is 77±3%, similar to that provided in the original Fig. 1d, while the mean PLQYs of the QD samples exposed to the UV in the air and Ar is obtained to be 7±3% and 52±1%, respectively. The reason for the PLQY decrease in the QDs exposed by UV in the Ar atmosphere is presumably the mild degradation of the QDs (Fig. 2n), identified in the HAADF-STEM and STEM-EDS analyses presented in Supplementary Fig. 11. Such degradation could be from the UV-facilitated oxidation by a trace amount of oxygen in the solvent or by photo-induced lattice structure degradation. We updated the revised version of Fig. 1d in the main text.

Original Figure (Fig. 1; page 20; in the main text)

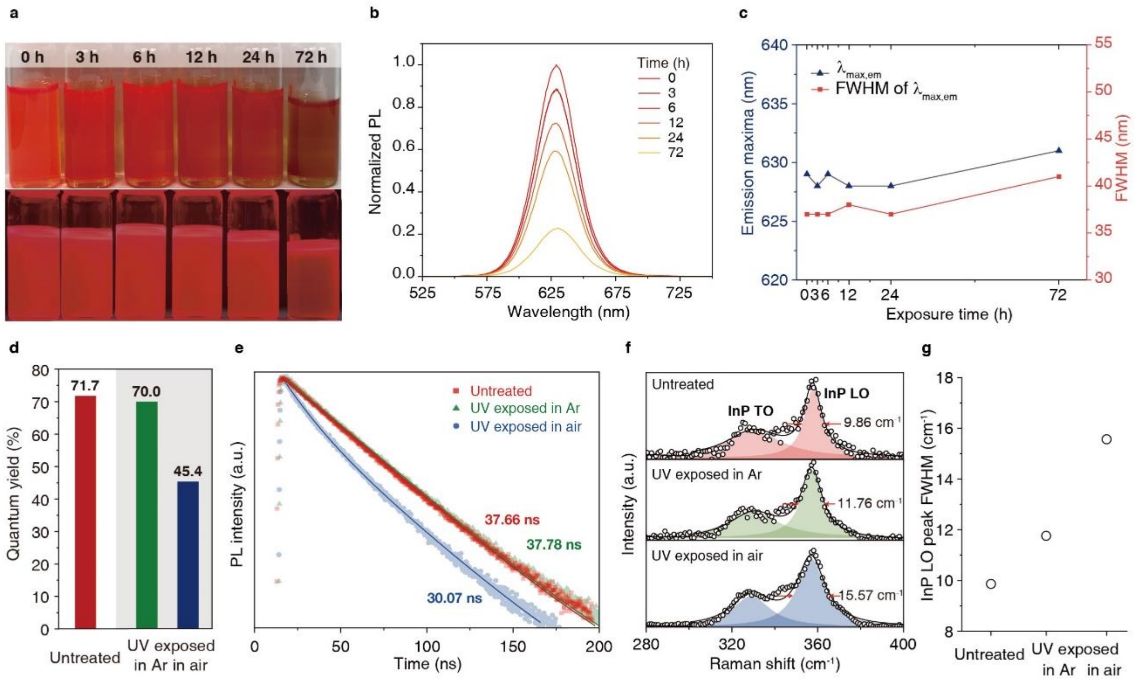

Fig. 1 | Photobleaching of an InP/ZnSe/ZnS QD solution under UV illumination. a Images of the QD solutions before UV exposure and after UV treatment under ambient conditions. Upper panels show images taken under ambient illumination. Lower panels show images taken under UV illumination. **b** PL emission spectra of the QD solutions after UV exposure over different times. **c** Changes in the emission peak maxima and FWHM of the PL emission spectra in (**b**). **d** PLQY changes after UV exposure in Ar and in air. **e** PL lifetime changes after UV exposure in Ar and in air. **f** Raman spectra of InP/ZnSe/ZnS QDs after UV exposure in Ar and in air. **g** FWHM of the InP LO peaks in (**f**).

Revised Figure (Fig. 1d; page 24; in the main text)

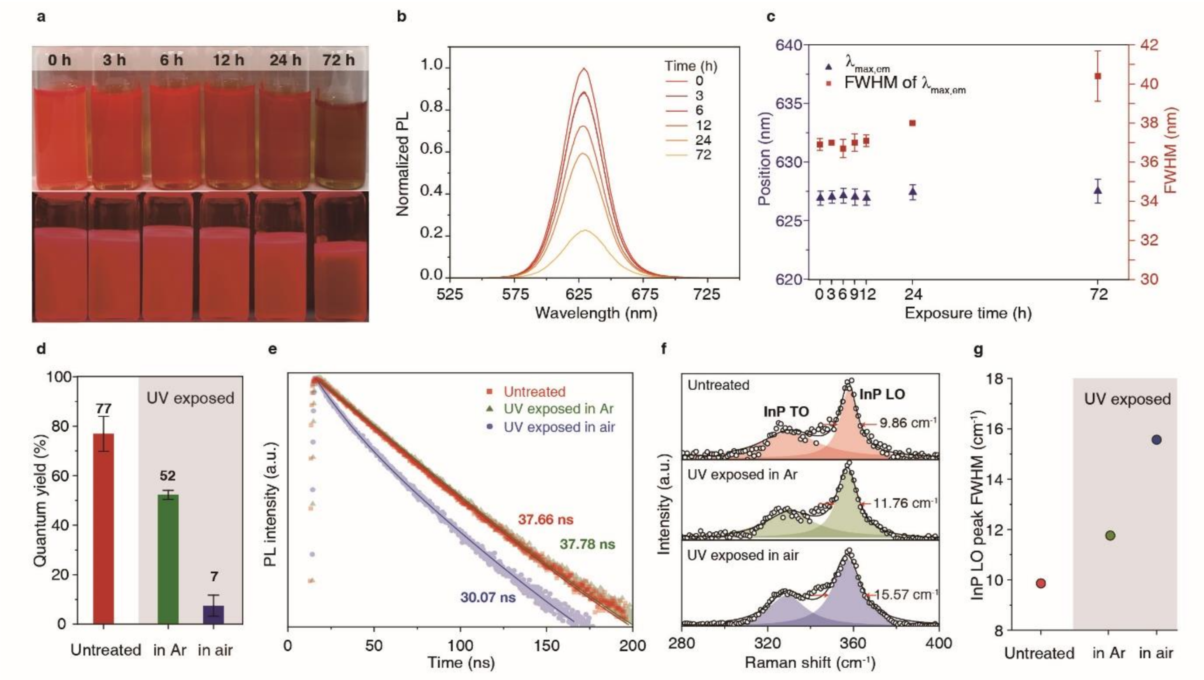

Fig. 1 | Photobleaching of an InP/ZnSe/ZnS QD solution under UV illumination. a Images of the QD solutions before UV exposure and after UV treatment under ambient conditions. Upper panels show images taken under ambient illumination. Lower panels show images taken under UV illumination. **b** PL emission spectra of the QD solutions after UV exposure over different times. **c** Changes in the emission peak maxima and FWHM of the PL emission spectra in (**b**). PL emission spectra is obtained by 10 separated measurements for each UV exposure circumstances, and the error bars indicate the standard deviations. **d** PLQY of untreated QD solution (red column), QD solution exposed to UV in air (blue column), and QD exposed to UV in Ar atmosphere (green column). The PLQY values are the mean of the 10 separated measurements, and the error bars indicate the standard deviations. **e** PL lifetime changes after UV exposure in Ar and in air. **f** Raman spectra of InP/ZnSe/ZnS QDs after UV exposure in Ar and in air. **g** FWHM of the InP LO peaks in (**f**).

Added Figure (Fig. 2n; page 26; in the main text)

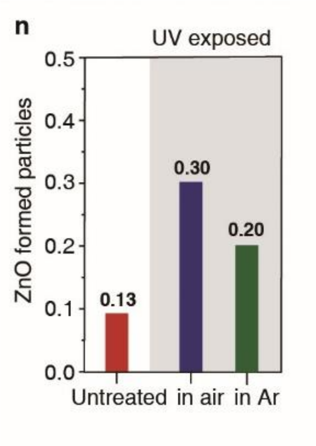

n Ratio of oxidized particles in untreated QDs (red column), QDs exposed to UV in air (blue column), and QD exposed to UV in Ar atmosphere (green column).

Added Figure (Supplementary Fig. 11; page 13; in the Supplementary Information)

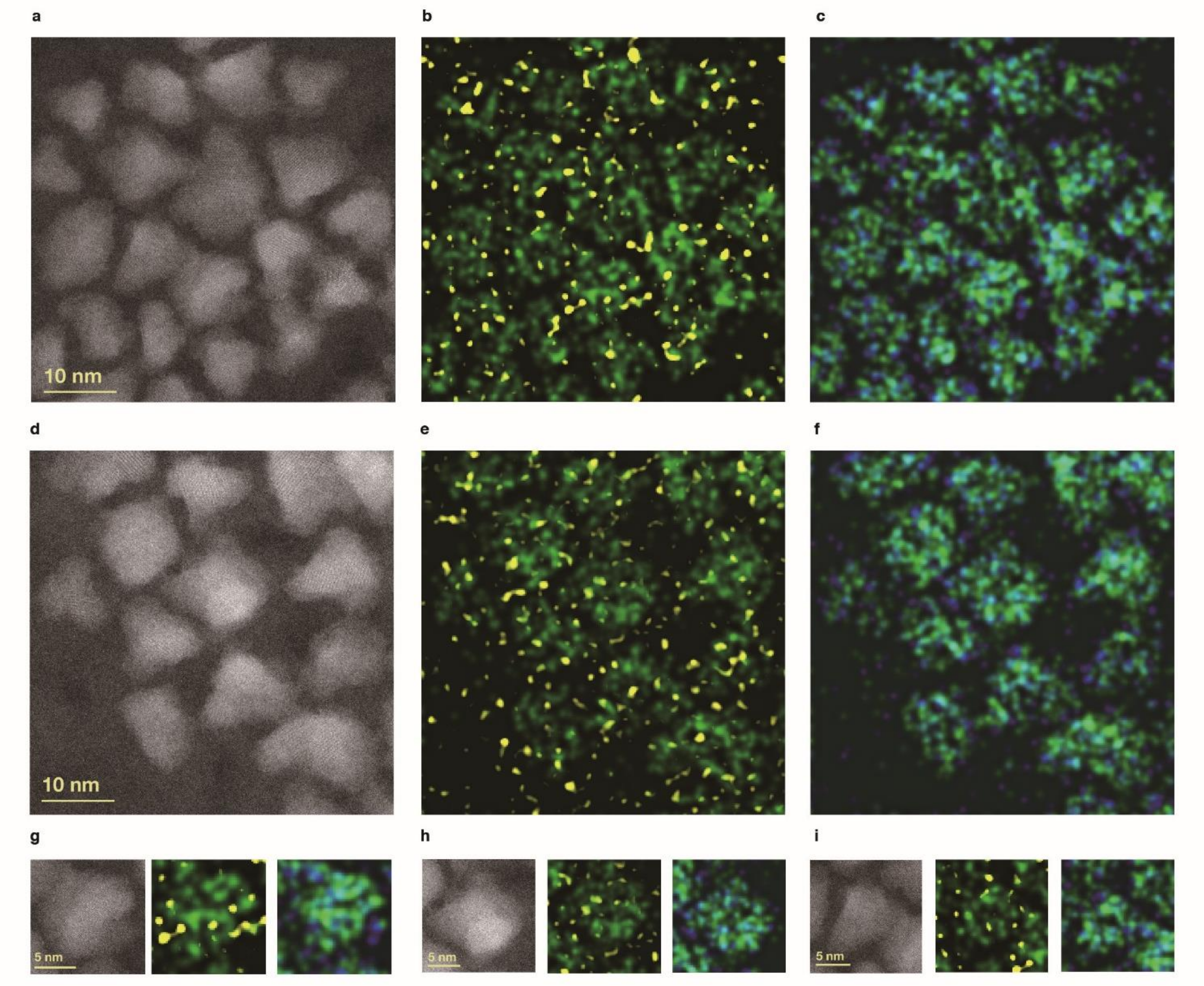

Supplementary Fig. 11 | EDS maps of ZnO and S-rich regions in single QDs exposed to UV light in Ar. a A HAADF-STEM image of QDs after UV exposure in Ar. **b,c** Corresponding EDS elemental maps for Zn and O (**b**), and for Zn, Se, and S (**c**). **d** Additional HAADF-STEM image taken at another site. **e,f** Corresponding EDS elemental maps for Zn and O (**e**), and for Zn, Se, and S (**f**). **g–i** Magnified views of single QDs in (**a**) (left panels) with the EDS elemental maps for Zn and O (middle panels) and for Zn, Se, and S (right panels). The colors in the EDS maps correspond to following elements: O (green), O (yellow), Se (cyan), S (blue).

Original text (page 5–6; in the main text)

...We conduct control experiments in which the QD solutions are exposed to UV light in an Ar atmosphere (Methods). UV exposure in an Ar atmosphere yields the mostly identical PLQY of the QD solution after 72 h of exposure, changing from 71.7% to 70.0% (green bar, Fig. 1d). ...

Revised text (page 6; in the main text)

... UV exposure in an Ar atmosphere yields a mild reduction in PLQY of the QD solution after 72 h of

exposure, from 77±3% to 52±1% (green bar, Fig. 1d), which is much smaller than the change in the PLQY caused by UV exposure in air....

Added text (Methods; page 16; in the main text)

... The PLQY values are the mean of the 10 independent measurements, and the error bars indicate the standard deviations (Fig. 1d)...

Comment #4:

This article has many formatting errors that need to be corrected, such as "thean" and "including InP- and Cd-based QDs and is frequently used…".

Response #4:

We thank Reviewer #2's careful comment on the manuscript. We thoroughly revised the manuscript to remove typos and improve styles. We would like to ask the reviewer to look into the revised manuscript.

Thank you very much for many appropriate and valuable comments. We are sure that these insightful comments have improved the quality of the manuscript significantly.

○ **Reviewer #3**

The present paper studies the degradation mechanism of InP/ZnSe/ZnS core/shell/shell QDs upon UV irradiation under air. In addition to Raman spectroscopy, STEM, EDS and EELS have been used on the single-particle level to identify the structural changes occurring during UV exposure in the presence or absence of air.

While these findings could be in principle of high interest for understanding the degradation mechanisms in InP-based QDs, the highly inhomogeneous character of the used QDs as visible from the TEM images (e.g., Fig. 2a,d) makes an unambiguous interpretation very delicate. There is a significant size and shape distribution already in the pristine sample without any light or air exposure. More spherical, state-of-the-art InP/ZnSe/ZnS QDs with lower size distribution have been obtained by working at higher temperature (340℃, cf. Nature 2019). Moreover, there are two strong hypotheses, which are in my opinion not fully supported by the data:

Response to the general comment: We highly appreciate the Reviewer #3's valuable comments. We have made our best efforts to answer the reviewer's comments, as detailed in our point-to-point responses below. During this revision, we significantly revised the entire manuscripts to address all the comments from the reviewer and to strengthen our claim on the QD oxidation process, by conducting i) advanced TEM analyses on the statistically meaningful numbers of QDs, ii) measurements of QD morphologies before and after the oxidation, and iii) suggestion of the possible oxidation mechanisms. Since these changes are made throughout the manuscript, we would like to ask the reviewer's favor to look into the manuscript as well as our point-to-point responses.

Comment #1:

oxydation of the outermost ZnS shell and formation of ZnO domains (cf. comment 7 below)

Response #1:

Thank you for the comment, we have responded your comment in the Response #7.

Comment #2:

diffusion of indium into the ZnSe shell: only Fig. 2 e/f seem to underpin this argument and this data is obtained from one single particle. The statistics on a larger number of QDs would be required to confirm this claim.

Response #2:

We appreciate the Reviewer #3's valuable comment. We have conducted additional STEM-EDS measurements for 50 QDs for the untreated and oxidized samples, and consistently found that the spatial distribution of In species are dispersed over the single QDs in the oxidized sample compared to those in the untreated samples, as shown in the revised Fig. 3, which show the EDS elemental maps of tens of QD particles in a single field of view. This can be also seen in the histogram showing the variance of In signal in EDS the maps, calculated by measuring dispersion of indium from the center of mass of the In in the EDS map as described in Methods. The histogram for the In signal variance in EDS maps (referred to scatterness in the previous version of the manuscript) of the oxidized (UV

exposed in the air) QD samples exhibit a broader distribution compared to that of the untreated sample. We believe that these results, analyzed for many QD particles for each treatment, indicate that the diffusion of In species occurs consistently by the oxidation of the QDs. These results are updated in the Fig. 3j in the main text.

Original Figure (Fig. 2; page 21; in the main text)

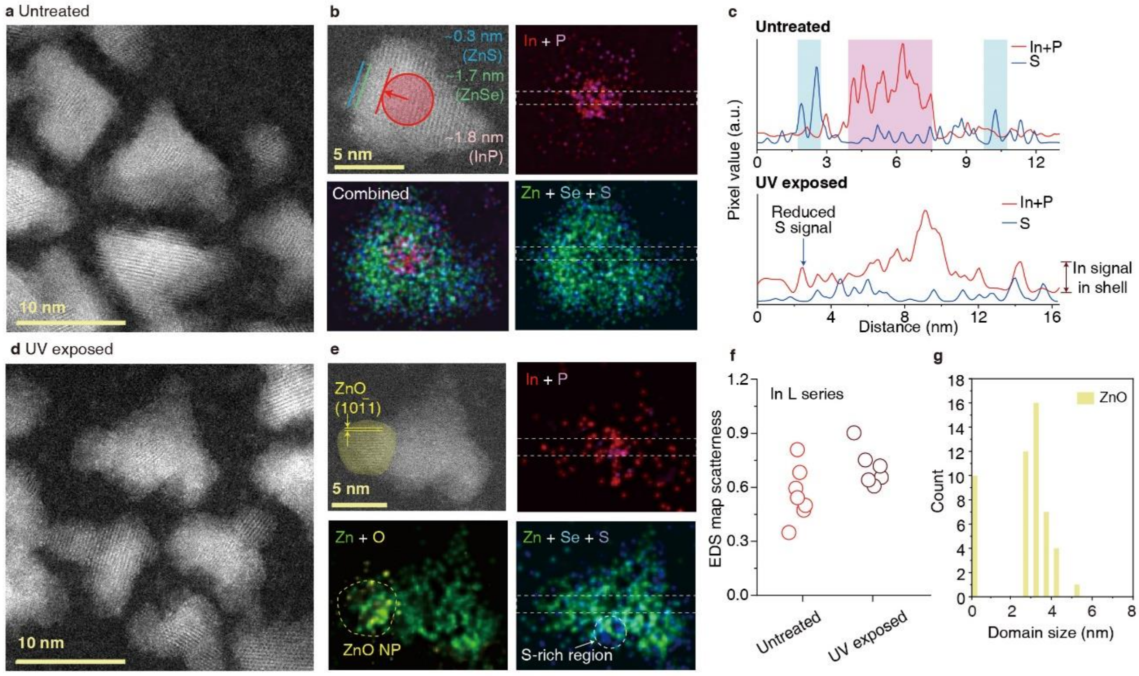

Fig. 2 | Structural changes in a single QD after photobleaching. a HAADF-STEM image of the untreated QD. **b** EDS mapping of In, P, Zn, Se, and S in the untreated. **c** Line scan from the EDS map of the untreated and UV-exposed QD. **d** HAADF-STEM image of UV-exposed QD. **e** EDS mapping of In, P, Zn, Se, and S in the UV-exposed QD. **f** Indium spatial distribution of the untreated and UV-exposed QD. **g** Size distribution of oxidized Zn species.

Revised Figure (Fig. 3; page 28; in the main text)

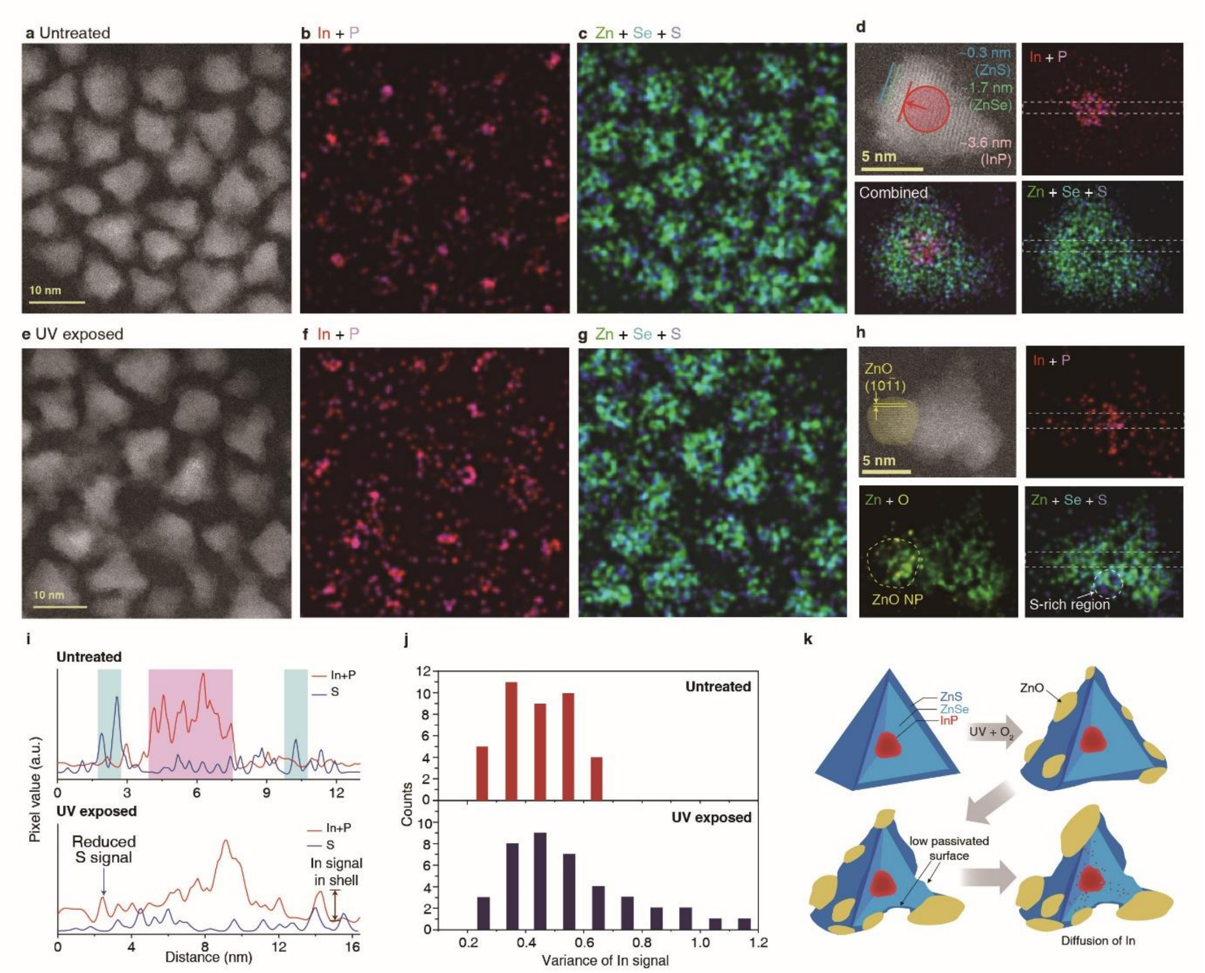

Fig. 3 | Chemical structure change of QDs by oxidation. a HAADF-STEM image of the QDs before oxidation**. b,c** Corresponding EDS elemental maps for In (red) and P (purple) (**b**) and for Zn (green), Se (cyan), and S (blue) (**c**). **d** EDS analysis of single QD before oxidation taken at a higher magnification. (upper left panel) HAADF-STEM image. (upper right panel) EDS elemental map for In and P. (lower right panel) EDS elemental map for Zn, Se, and S. (lower left panel) EDS elemental map showing all the elements. **e** HAADF-STEM image of the QDs after oxidation**. f,g** Corresponding EDS elemental maps for In (red) and P (purple) (**f**) and for Zn (green), Se (cyan), and S (blue) (**g**). **h** EDS analysis of single QD after oxidation taken at a higher magnification. (upper left panel) HAADF-STEM image. (upper right panel) EDS elemental map for In and P. (lower right panel) EDS elemental map for Zn, Se, and S. (lower left panel) EDS elemental map showing all the elements. **i** Line profiles extracted from the EDS map for In and P along the line marked in (**d**) and (**h**)**. j** Variance of In signals in the EDS elemental maps, measured for QD before (upper panel) and after (lower panel) the UV-facilitated oxidation. **k** Schematic illustration for the proposed degradation mechanism of InP/ZnSe/ZnS QDs by the UV-facilitated oxidation**.**

Added text (Methods; page 18–19; in the main text)

Calculation of In signal variance

The spatial distribution of In species was calculated from the EDS elemental maps for In by defining "variance of EDS signal". The variance of In signal in the EDS map is an average of the signal intensities weighted by the distance of each signal from the center of mass of the In signal in a 2D EDS map, followed by a normalization. It has a value of 0 if the In signal is detected only at a single pixel in a 2D EDS elemental map, and has a value of 1 if the In signal is uniformly distributed over a 2D map. To calculate the signal variance of In species over a single QD, we first cropped a 2D EDS elemental map for In to include a single QD and then, calculated the locations (x_{center}, y_{center}) of center of mass of the In signals in the cropped image. The distance-weighted signal average is calculated by multiplying a matrix describing the distance from a pixel at (x_i, y_j) to the center of mass by the 2D EDS elemental map for In:

$$S = \frac{1}{N}\sum_{i,j}\sqrt{(x_i - x_{center})^2 + (y_j - y_{center})^2} \cdot I$$

where I indicates a 2D EDS elemental map for In, and N indicates the total number of pixels in the map. Finally, S is divided by the S_{ref}, which is calculated for a virtual EDS map composed of uniform signals in the same way.

Added text (page 10; in the main text)

... These results are confirmed by comparing the variance of the In signals over the EDS maps of single QDs for 50 untreated and oxidized QDs (Fig. 3j and Methods)....

Comment #3:

The synthesis procedure for the used QDs needs to be given, at least in SI.

Response #3:

Thank for the Reviewer #3's helpful comment. We have updated the synthesis procedure in the supplementary information. The brief explanation of the synthesis procedure is follows: i) InP core is synthesized with In acetate and TMS_3P as In and P precursor, and palmitic acid as ligands. ii) InP core crude solution is cleaned before shell passivation. iii) Zn oleate, Zn acetate and oleic acid is dissolved in trioctylamine, and then heated to synthetic temperature. iv) The InP core solution is injected into the synthesis medium and Zn oleate and Se/TOP solution is injected for ZnSe shelling, and for ZnS shelling, Zn oleate and S/TOP solution is added afterward.

Added Text (Methods; page 14–15; in the main text)

Synthesis process

InP/ZnSe/ZnS QD solution was synthesized by Samsung Display Ltd and the procedure for the synthesis of the InP/ZnSe/ZnS QD is reported elsewhere[54-57]. Briefly, InP core was synthesized with

using indium acetate ($In(OAc)_3$) and trimethylsilyl phosphine (TMS_3P) as In and P precursor, and palmitic acid as ligands. The $In(OAc)_3$ and palmitic acid were dissolved in octadecene and then evacuated using a rotary pump. Under the flow of N_2 gas, the solution was heated to the temperature of 280 °C, at which the TMS_3P dissolved in trioctylphosphine (TOP) was quickly injected to the solution. The InP core crude solution was cleaned for the further shell passivation. For shell passivation, Zn oleate, zinc acetate ($Zn(OAc)_2$) and oleic acid dissolved in trioctylamine were degassed, and then heated to 280 ºC under a N_2 flow. Then, the temperature is dropped to 180 ºC and the InP core solution was injected quickly. Afterwards, the mixture was heated again to 340 ºC and Zn oleate and Se/TOP were added to the mixture for ZnSe passivation. Finally, Zn oleate and the S/TOP were added to the mixture for further ZnS passivation. The red QD solution was stored inside a glove box ($O_2 < 0.5$ ppm) to avoid any oxygen contact.

Comment #4:

On p.8 the authors state that the InP core diameter is 1.8 nm, which does not seem to match an emission at 630 nm.

Response #4:

Thank you for the careful comment. We have found that there were typos in the main text and corrected them. The diameter of the InP core is 3.6 nm, which is consistent with the reference [*Nature* (2019) **575**, 634-638]. We apologize for the confusion and updated the manuscript and the Fig. 3d. Furthermore, we have thoroughly proof-read the entire manuscript for the typos and styles to improve the quality of the writings.

Original Figure (Fig. 2b; page 21; in the main text)

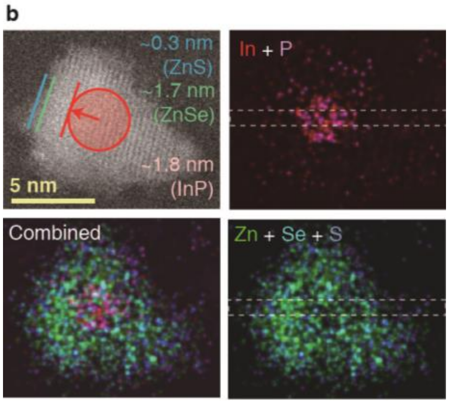

b EDS mapping of In, P, Zn, Se, and S in the untreated.

Revised Figure (Fig. 3d; in the main text)

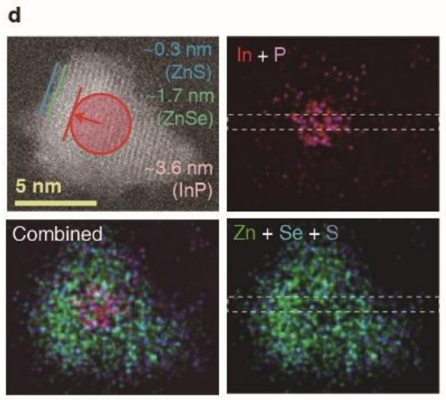

d EDS analysis of single QD before oxidation taken at a higher magnification. (upper left panel) HAADF-STEM image. (upper right panel) EDS elemental map for In and P. (lower right panel) EDS elemental map for Zn, Se, and S. (lower left panel) EDS elemental map showing all the elements.

Original text (page 4; in the main text)

… These QDs are composed of an approximately 1.8-nm-diameter InP core, a 2-nm-thick ZnSe shell, and an approximately 0.2-nm-thick outermost ZnS shell. …

Revised text (page 4; in the main text)

… These QDs consist of an approximately 3.6-nm-diameter InP core, a 2-nm-thick ZnSe shell, and an approximately 0.2-nm-thick outermost ZnS shell. …

Comment #5:

The observed (although slight) red-shift of the PL after photo-oxidation is unexpected and the cited Zn diffusion into the InP lattice (p. 6) should rather lead to a blue-shift. Please comment.

Response #5:

We are thankful for the Reviewer #3's insightful comment. We have first checked the consistency of our PL measurements by measuring the emission spectra of ten samples for each oxidation time (0, 3, 6, 9, 12, 24, and 72 h), and plotted the mean values with their standard deviations as shown in the revised Fig. 1c. From the results, we found out that the mentioned shift of the maximum PL emission wavelength ($\lambda_{max,em}$) is within the error ranges (Fig. 1c), which suggest that the $\lambda_{max,em}$ does not show noticeable shift by the oxidation of the QDs. Nonetheless, as the reviewer mentioned, the observed structural changes may induce the shift of $\lambda_{max,em}$. However, we would like to mention that we have observed several types of structural changes in the QDs induced by the oxidation and these changes, within the marginal degree we study here, would make the emission peak shift complex. For example, the Zn diffusion into the InP core may contribute to blue-shift, while the In diffusion into the ZnSe shell may induce red-shift by making the effective core size larger. Furthermore, our detailed TEM-based characterization revealed that some of the outer ZnS shell is oxidized to ZnO, which lead to the

incomplete passivation of the ZnSe shell. The imperfect exciton confinement originated from the poor passivation is likely to further shift the emission wavelength. Having considered these counter-acting contributions and the fact that the degree of UV-facilitated compositional changes are small which occur within a range that does not induce disintegration of QD domain, it is more reasonable that structural changes are represented as a widened FWHM and larger error bar in the $\lambda_{max,em}$ of the QD solution oxidized for 72 h, instead of noticeable peak shift, as can be seen in the revised Fig. 1c. We added discussion on this in the revised manuscript.

Original Figure (Fig. 1; page 20; in the main text)

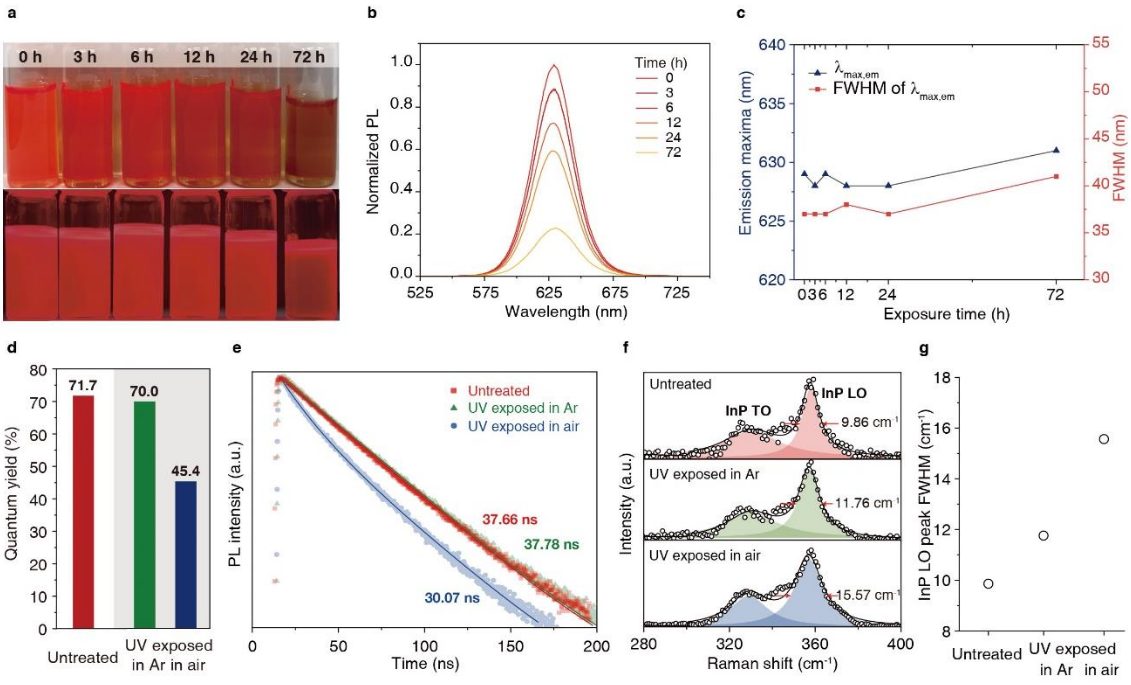

Fig. 1 | Photobleaching of an InP/ZnSe/ZnS QD solution under UV illumination. a Images of the QD solutions before UV exposure and after UV treatment under ambient conditions. Upper panels show images taken under ambient illumination. Lower panels show images taken under UV illumination. **b** PL emission spectra of the QD solutions after UV exposure over different times. **c** Changes in the emission peak maxima and FWHM of the PL emission spectra in (**b**). **d** PLQY changes after UV exposure in Ar and in air. **e** PL lifetime changes after UV exposure in Ar and in air. **f** Raman spectra of InP/ZnSe/ZnS QDs after UV exposure in Ar and in air. **g** FWHM of the InP LO peaks in (**f**).

Revised Figure (Fig. 1c; page 25; in the main text)

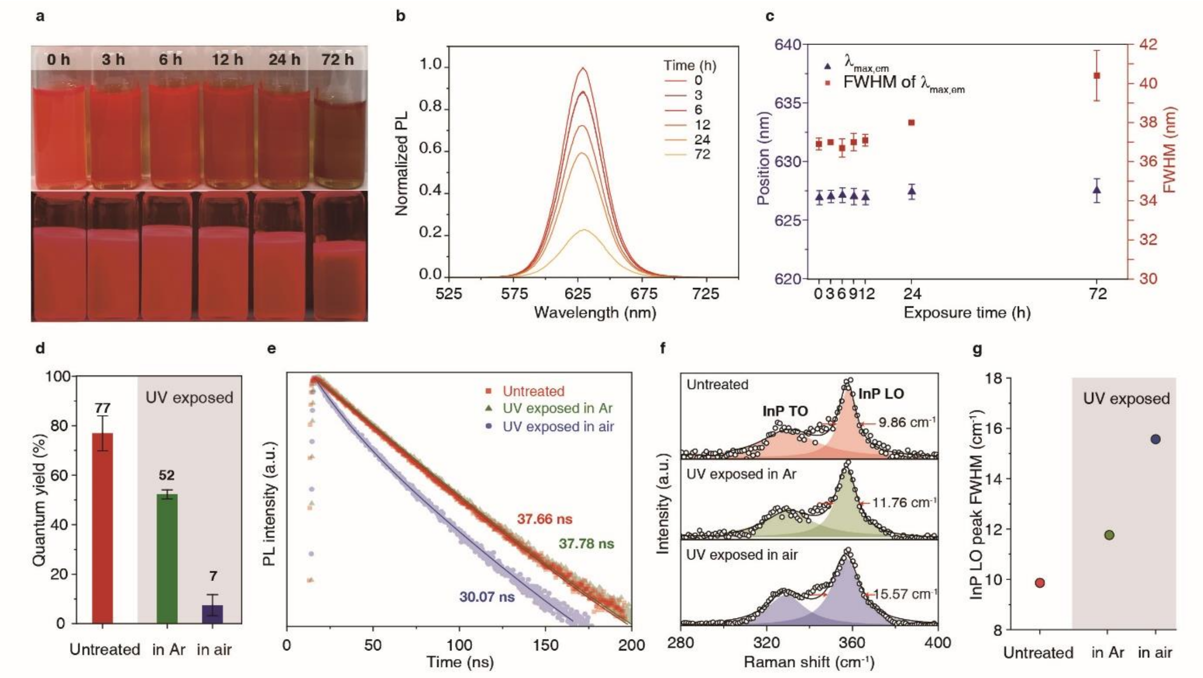

Fig. 1 | Photobleaching of an InP/ZnSe/ZnS QD solution under UV illumination. a Images of the QD solutions before UV exposure and after UV treatment under ambient conditions. Upper panels show images taken under ambient illumination. Lower panels show images taken under UV illumination. **b** PL emission spectra of the QD solutions after UV exposure over different times. **c** Changes in the emission peak maxima and FWHM of the PL emission spectra in (**b**). PL emission spectra is obtained by 10 separated measurements for each UV exposure circumstances, and the error bars indicate the standard deviations. **d** PLQY of untreated QD solution (red column), QD solution exposed to UV in air (blue column), and QD exposed to UV in Ar atmosphere (green column). The PLQY values are the mean of the 10 separated measurements, and the error bars indicate the standard deviations. **e** PL lifetime changes after UV exposure in Ar and in air. **f** Raman spectra of InP/ZnSe/ZnS QDs after UV exposure in Ar and in air. **g** FWHM of the InP LO peaks in (**f**).

Original text (page 6; in the main text)

InP/ZnSe/ZnS QDs oxidized by UV irradiation exhibit emissions mediated by trap states. The QDs oxidized over different times from 0 to 72 h exhibit a slight red shift in the PL emission peak from 625 to 628 nm. Furthermore, the full width at half maximum (FWHM) of the emission peak increases from 37 to 41 nm, as shown in Fig. 1c. Such red-shift and broadening of the emission peak have also been previously observed in InP/ZnSe core/shell QDs, where Zn substitutes into the lattice of the InP cores[19]. The inclusion of Zn within the InP cores is known to induce the formation of shallow hole traps, resulting in a broad and red-shifted emission from the QD[19,29-31]. Therefore, it can be hypothesized that the photo-oxidized InP/ZnSe/ZnS QDs exhibiting broadened emission peaks also possess trap-associated emission pathways[28,32]. ...

Revised text (page 6; in the main text)

... While the maximum PL emission wavelength, $\lambda_{max,em}$, of the QDs remains similar around 627 nm

during the oxidation from 0 to 72 h, the full width at half maximum (FWHM) of the emission peak increases from 37 to 41 ± 1 nm, as shown in Fig. 1c. Such broadening of the emission peak is consistent with the previous observations where impurities in the lattice of the ZnSe shell induce emission broadening of InP/ZnSe core/shell QDs [20]. The inclusion of impurities, such as indium species, within the ZnSe shell is known to induce the formation of shallow hole traps, resulting in a broad emission from the QD[30-32]....

Added text (Methods; page 15; in the main text)

... The PL emission is obtained for 10 independent QD solution samples for each time of UV exposure, and mean values are indicated with error bars which represent standard deviations (Fig. 1c).

Comment #6:

The accuracy of PLQY measurements is overestimated, no digit after the comma should be given, and the error indicated.

Response #6:

Thank you for the careful comment. Similar to the Comment #5, we have measured PLQYs of ten samples for each treatment (untreated, UV exposure in the air, UV exposure in the Ar atmosphere). The mean PLQY of the ten measurements with the standard deviation are presented in the revised Fig. 1d, which is presented in the Response #5. The mean PLQY of the untreated samples is 77±3%, while the mean PLQYs of the QD samples exposed to the UV in the air and Ar is obtained to be 7±3% and 52±1%, respectively. The size of error bars is an order of one percent, so we removed the digits after the comma in the figures, tables, and manuscript, as the reviewer mentioned. We are thankful to the reviewer for pointing out.

Original text (page 5; in the main text)

... The measured PL quantum yield (PLQY) of the QD solution shows an approximately 26% reduction after the 72 h of the UV exposure, from 71.7% to 45.4%, as shown in Fig. 1d. ...

Revised text (page 5; in the main text)

... The measured PL quantum yield (PLQY) of the QD solution shows an approximately 70% reduction after 72 h of the UV exposure, from 77 ± 3% to 7 ± 3%, as shown in Fig. 1d (Methods). ...

Original text (page 5–6; in the main text)

... UV exposure in an Ar atmosphere yields the mostly identical PLQY of the QD solution after 72 h of exposure, changing from 71.7% to 70.0% (green bar, Fig. 1d). ...

Revised text (page 6; in the main text)

... UV exposure in an Ar atmosphere yields a mild reduction in PLQY of the QD solution after 72 h of

exposure, from 77±3% to 52±1% (green bar, Fig. 1d), which is much smaller than the change in the PLQY caused by UV exposure in air. ...

Added text (Methods; page 16; in the main text)

... The PLQY values are the mean of the 10 independent measurements, and the error bars indicate the standard deviations (Fig. 1d). ...

Comment #7:

Sup. Inf. Fig. 5: what do the authors mean with « clean surfaces »? The surfaces of the tetrahedral particles are for sure rough and not smooth, which is clearly visible in the images and has been demonstrated in other studies

Response #7:

We thank the Reviewer #3 for the insightful comment. We tried to express the surface morphology changes in QDs after the UV exposure, which can be noticed in the revised Fig. 2 and Supplementary Fig. 8 and further supported by estimating the circularities of QDs (Methods). We also provided additional images such as the morphology of single QD in high magnification (Supplementary Fig. 4), and additional HAADF-STEM images showing morphological changes of QDs (Supplementary Fig. 5). We revised the manuscript to avoid confusion.

Original Figure (Fig. 2; page 21; in the main text)

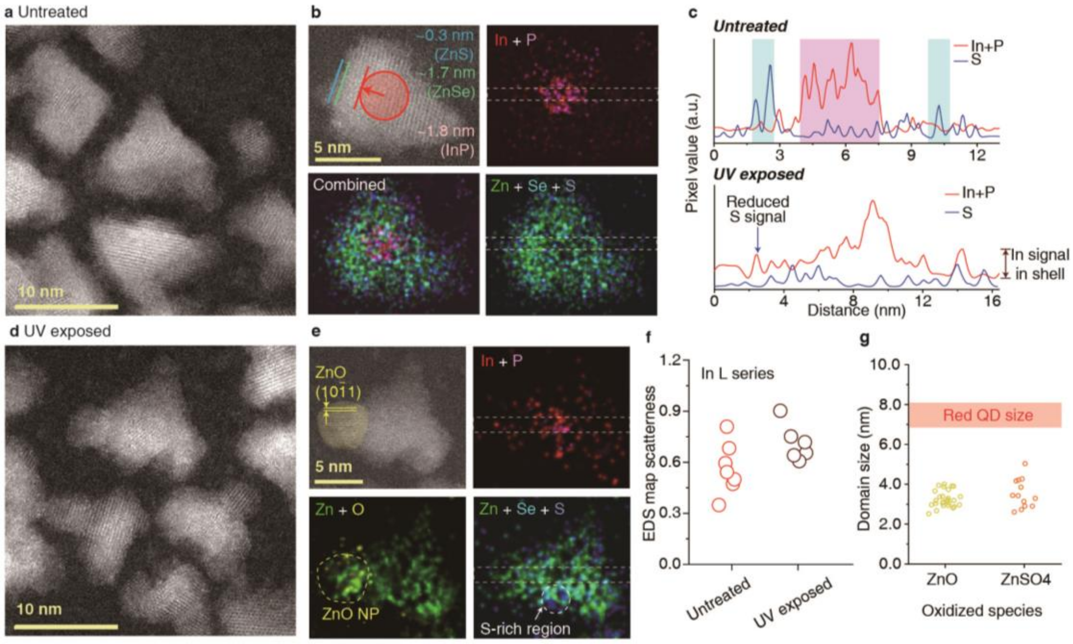

Fig. 2 | Structural changes in a single QD after photobleaching. a HAADF-STEM image of the untreated QD. **b** EDS mapping of In, P, Zn, Se, and S in the untreated. **c** Line scan from the EDS map of the untreated and UV-exposed QD. **d** HAADF-STEM image of UV-exposed QD. **e** EDS mapping

of In, P, Zn, Se, and S in the UV-exposed QD. **f** Spatial distribution of indium in the untreated and UV-exposed QD. **g** Size distribution of oxidized Zn species.

Revised Figure (Fig. 2a–2e; page 26; in the main text)

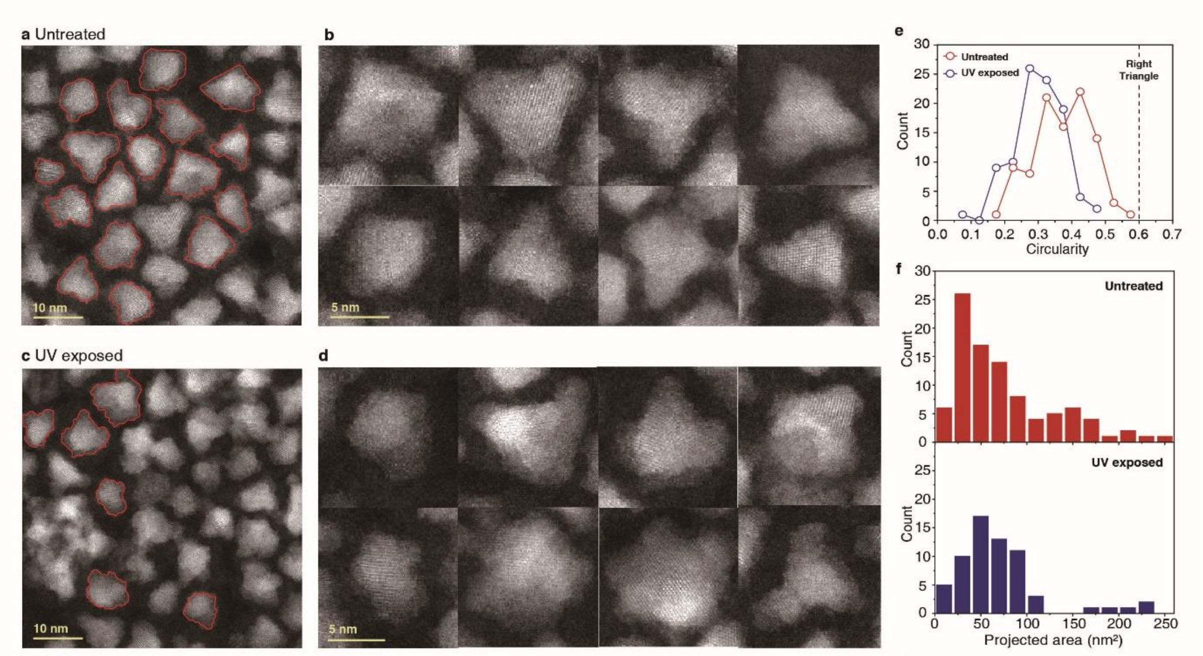

a HAADF-STEM image of the QDs before the UV-facilitated oxidation. (red line) Contours of the QDs used for the calculation of circularity. **b** Enlarged images of representative QDs before the UV-facilitated oxidation. **c** HAADF-STEM image of the QDs after the UV-facilitated oxidation for 72 h. (red line) Contours of the QDs used for the calculation of circularity. **d** Enlarged images of representative QDs after the UV-facilitated oxidation. **e** Circularity distributions of QDs before (red symbols) and after (blue symbols) the UV-facilitated oxidation. (dashed line) Circularity of a right triangle, 0.61. **f** Distributions of 2D projected area of QDs before (upper panel) and after (lower panel) the UV-facilitated oxidation.

Added Figure (Supplementary Fig. 4; page 5; in the Supplementary Information)

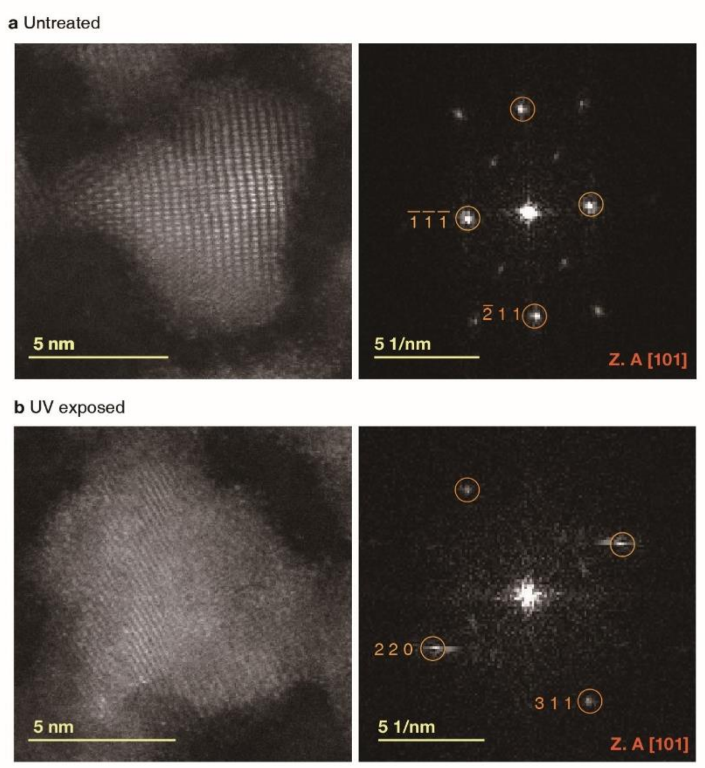

Supplementary Fig. 4 | Morphology of single QD in high magnification. a,b HAADF-STEM images (left) and corresponding FFTs (right) of a QD before (**a**) and after (**b**) the UV-facilitated oxidation.

Added Figure (Supplementary Fig. 5; page 6; in the Supplementary Information)

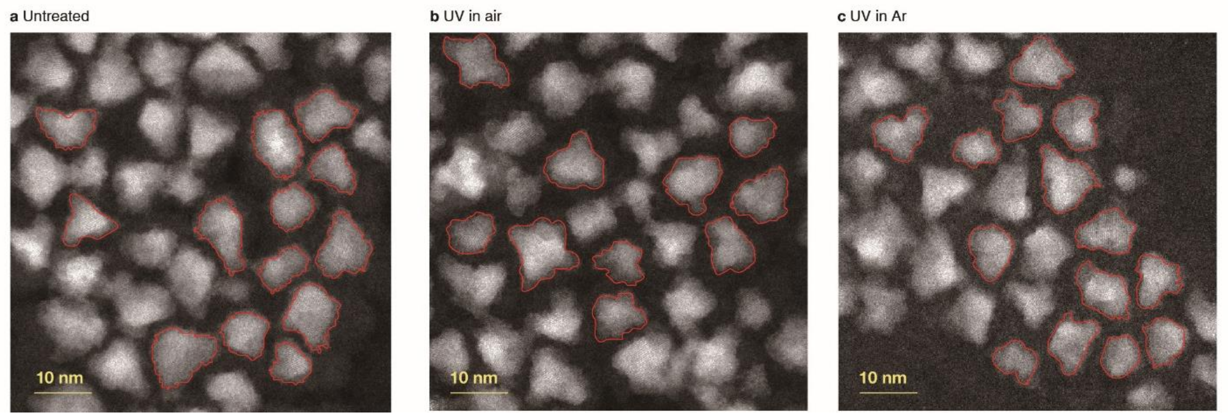

Supplementary Fig. 5 | Additional HAADF-STEM images showing morphological changes in QDs with different treatments. a–c HAADF-STEM images of QDs in untreated QDs (**a**), QDs exposed to UV in air (**b**), and QDs exposed to UV in Ar (**c**). (red lines) Contours of QDs used to measure their 2D projected areas and circularities (Methods).

Added Figure (Supplementary Fig. 8; page 9; in the Supplementary Information)

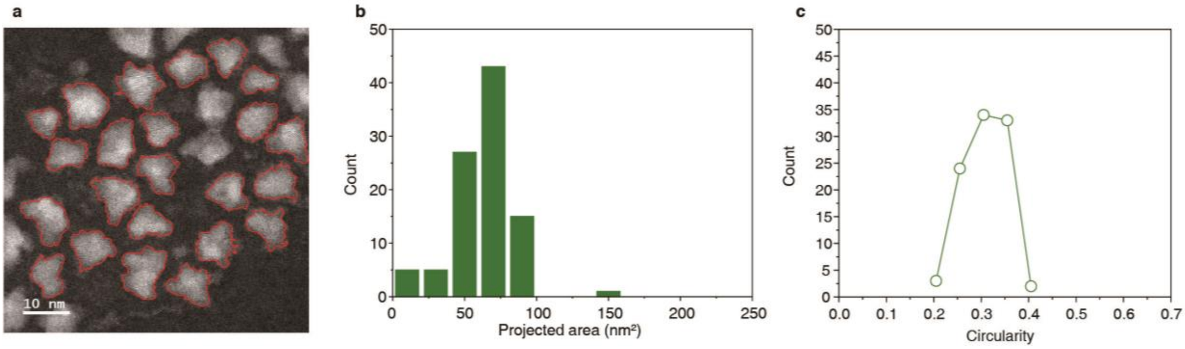

Supplementary Fig. 8 | Morphology of QDs exposed to UV light in Ar. a HAADF-STEM image of QDs exposed to UV light in Ar. (red lines) Contours of QDs used to measure their 2D projected areas and circularities. **b** Projected area distribution of the QDs. **c** Circularity distribution of the QDs. The measured 2D projected area and circularity are similar to those of the untreated QDs (Fig. 2e and f).

Added text (Methods; page 18; in the main text)

Calculation of circularity

Circularity can be used to describe the surface roughness of the irregularly-shaped nanoparticles[52]. To investigate the surface roughness of the QDs before and after the UV-facilitated oxidation, we calculated the circularity of the QDs using the HAADF-STEM images. First, the images were binarized. Then, the contours of the QDs were identified from the binarized images, which enabled the calculation of the perimeters and 2D projected area of the QDs. The circularity, *C*, is calculated based on the equation below.

$$C = \frac{A}{D^2}$$

where *A* indicates the 2D projected area of a particle and *D* indicates the perimeter of the same particle. In HAADF-STEM images of the QDs, aggregated and vertically-overlapped QD particles, presumably formed during TEM sample preparation processes, were often found. We excluded those QD particles from the circularity calculation for accurate measurement, and only the isolated QD particles (marked in red contours in Fig. 2a and c) were used for the calculation. 96, 90, and 97 particles were measured for the pristine sample (without a treatment), sample exposed to UV in the air, and sample exposed to UV in an Ar atmosphere, respectively.

Original text (page 8; in the main text)

... High-resolution STEM images of the InP/ZnSe/ZnS QDs before and after 72 h of UV exposure are presented in Fig. 2. The untreated QDs mostly exhibit triangular shapes, projections of the tetrahedral morphology of QDs (Fig. 2a), and consist of single-crystalline domains (Supplementary Fig. 4). The EDS elemental maps show that the untreated QDs exhibit a well-defined InP/ZnSe/ZnS core/shell/shell structure. The InP core is spherical with a diameter of approximately 1.8 nm and is located at the center of the QD. The core is covered with a 1.7-nm-thick ZnSe shell, which is fully passivated by a 0.3-nm-

thick ZnS shell (Fig. 2b and 2c). Some of the UV-exposed QDs exhibit a locally rough surface, but their size does not dramatically change, as confirmed by the high-angle annular dark-field (HAADF) STEM images in Fig. 2d and Supplementary Fig. 4. ...

Revised text (page 8; in the main text)

... . The untreated QDs mostly exhibit shapes similar to triangles, the projections of a tetrahedral morphology (Fig. 2a and 2b), and consist of mostly single-crystalline domains, as shown in high-resolution high-angle annular dark-field (HAADF) STEM images (Fig. 2b and Supplementary Fig. 4). The UV-exposed QDs mostly exhibit more irregular shapes with rough surface and large size variations compared to the untreated QDs (Fig. 2c, 2d, and Supplementary Fig. 4). The difference in the particle morphology and surface structures is more clearly indicated from the 2D projected areas and circularities of the QDs, measured by identifying their contours marked with the red lines in Fig. 2a and 2c (Methods). In the UV-exposed QDs, the circularities measured for approximately 100 QD particles have smaller values (Fig. 2e, blue symbols) compared to those of the untreated QDs (Fig. 2e, red symbols) and a reference value for a right triangle (Fig. 2e, dashed line), due to the rough surface characteristic of the UV-exposed QDs (Supplementary Fig. 4 and Supplementary Fig. 5).

Revised text (page 9; in the main text)

... On the other hand, the QDs exposed to UV in an Ar atmosphere exhibit little morphological changes compared to the untreated QDs, as shown in Supplementary Fig. 5 and 8....

Comment #8:

The PL decay curves and fits should be given in SI, together with the individual lifetimes and not just average lifetime. Two- and three-exponential fits should be compared.

Response #8:

We appreciate the Reviewer #3's valuable comment. We added the PL decay curves and corresponding fits as Supplementary Fig. 2 and Supplementary Table 1. In our manuscript, the decay curves were fitted with triexponential functions, and the averages of the fitted lifetimes were presented in the Fig. 1e. The exponential equation for the fitting is in the "Methods" section. Also, we added the results from the bi- and tri-exponential fits.

Added Figure (Supplementary Fig. 2; page 3; in the Supplementary Information)

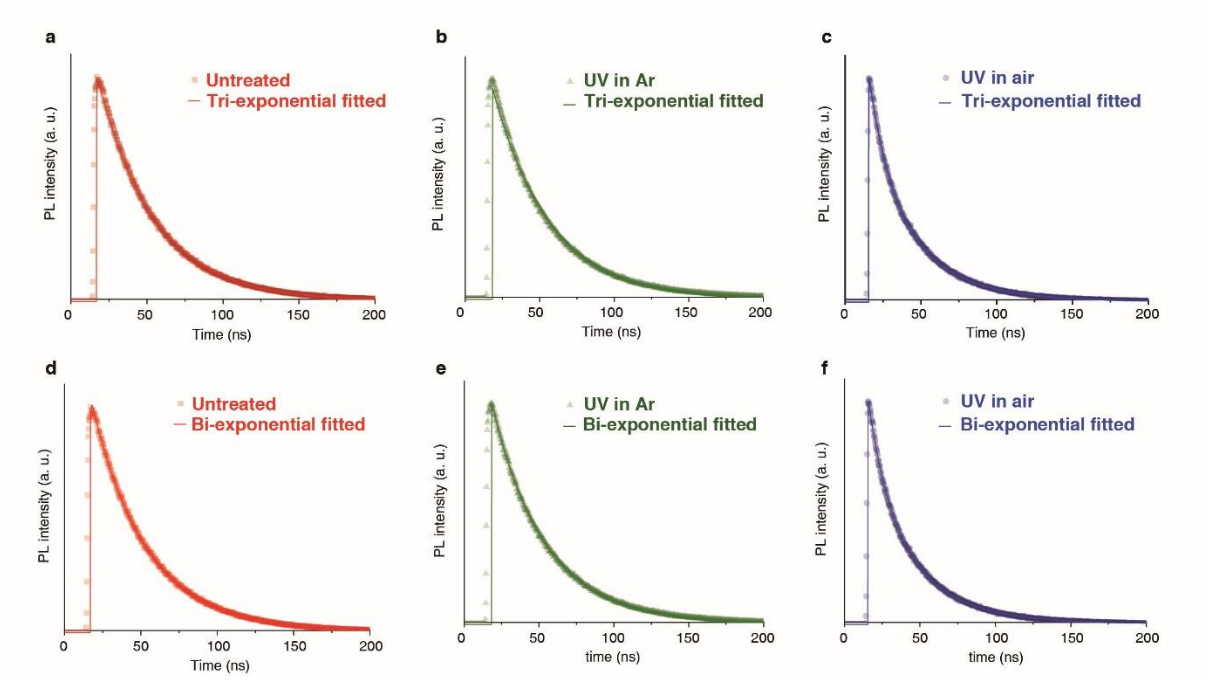

Supplementary Fig. 2 | PL decay curves of untreated and UV-exposed QDs. a–c PL decay curves for a untreated QD sample (**a**), QD sample exposed to UV in Ar (**b**), and QD sample exposed to UV in air (**c**). (lines) Fits by tri-exponential function. **d–f** PL decay curves for the untreated QD sample (**d**), QD sample exposed to UV in Ar atmosphere (**e**), and QD sample exposed to UV in air (f). (lines) Fits by bi-exponential function. See also for Supplementary Table 1 and Methods for details.

Added Table (Supplementary Table 1; page 20; in the Supplementary Information)

Supplementary Table 1 | a,b Fitted pre-exponentials (*A*) and lifetimes (τ) of PL decay curves presented in Supplementary Fig. 2 by tri-exponential function (**a**) and bi-exponential function (**b**).

a

Lifetime (pre-exponential)	τ_1 (A_1)	τ_2 (A_2)	τ_3 (A_3)	χ^2
Untreated	1.59 ns (-0.16)	32.17 ns (0.35)	53.71 ns (0.084)	1.07
UV in Ar	9.38 ns (5.65E-3)	54.40 ns (0.085)	31.76 ns (0.32)	1.03
UV in air	25.39 ns (0.24)	45.03 ns (8.51E-2)	7.21 ns (0.12)	1.11

b

Lifetime (pre-exponential)	τ_1 (A_1)	τ_2 (A_2)	χ^2
Untreated	32.52 ns (0.34)	54.38 ns (7.60E-2)	1.10
UV in Ar	30.59 ns (0.30)	51.71 ns (0.11)	1.03
UV in air	14.01 ns (0.21)	36.67 ns (0.22)	1.27

Added text (Methods; page 16; in the main text)

PL decay curves shown in Fig. 1e in the main text were fitted with tri-exponential function, which is shown below:

$$y = y_0 + A_1 e^{-t/\tau_1} + A_2 e^{-t/\tau_2} + A_3 e^{-t/\tau_3}.$$

The fitted results are presented in Supplementary Fig. 2 and the obtained pre-exponentials, *A*, and lifetimes, τ, are presented in Supplementary Table 1. The mean value of the three lifetimes obtained by the fitting of each sample was presented in the Fig. 1e. For comparison, we also fitted the PL decay curves with bi-exponential function, shown below:

$$y = y_0 + A_1 e^{-t/\tau_1} + A_2 e^{-t/\tau_2}.$$

The fitted results and parameters are also presented in Supplementary Fig. 2 and Supplementary Table 1.

Comment #9:

In the comparison of Fig. SI 5 and 6, both of them exhibit small spherical particles on top of the large-sized tetrahedra. Where do they come from in the case of the sample kept under Ar?

Response #9:

We are thankful for the Reviewer #3's helpful comment. In order to response the reviewer's comment, we would like to mention that we have found that the small spherical particles are formed in the oxidized QD samples (exposed to the UV under the air). Analysis of lattice structures in the HAADF-STEM images have confirmed that these spherical nanoparticles are ZnO (Fig. 2n) and presumably formed by the oxidation of the shell of the QDs; we have revealed that the oxidation of the QDs leads to the roughening of the QD surfaces accompanied by the formation of ZnO, and the additional UV-facilitated oxidation treatment might induce the formation of ZnO nanoparticles.

As mentioned in the Response #6, we have found that the PLQY of the QD samples exposed to UV in the Ar atmosphere is smaller than the untreated QDs, although we stored the QD sample in a glove box with O_2 concentration fewer than 0.5 ppm. The trace oxidation of the QDs, perhaps through a contact with the air during sample treatment between analysis, is responsible for the formation of the small nanoparticles the reviewer mentioned. We have further quantified the population of ZnO nanoparticles in each sample to support that the oxidation induces the formation of the small ZnO nanoparticles. The ZnO nanoparticles were identified from the HAADF-STEM images by measuring the lattice spacings of the QDs in fast Fourier transforms and comparing the measured values with known interplanar distances of ZnO using a home-written scripts in MATLAB (MathWorks). The detailed workflow of the scripts is shown in the Supplementary Fig. 7. As shown in Fig. 2n, the fraction of the ZnO-formed QD particles are highest (30%) in the QD sample exposed to the UV in the air, and the fraction of the ZnO-formed QD particles is slightly lower (20%) in the case of UV exposed in Ar but still higher than that of the untreated sample. This result indicates that the slight oxidation of the QDs leads to the formation of small ZnO nanoparticles, even though the UV treatment was performed in an air-tight environment.

Added Figure (Fig. 2n; page 26; in the main text)

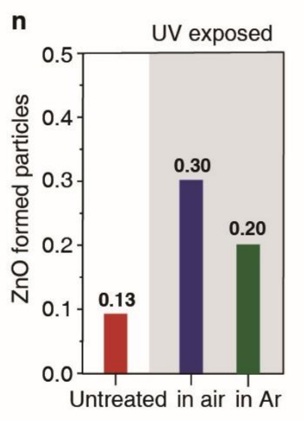

n Ratio of oxidized particles in untreated QDs (red column), QDs exposed to UV in air (blue column), and UV exposed in Ar (green column).

Added Figure (Supplementary Fig. 7; page 8; in the Supplementary Information)

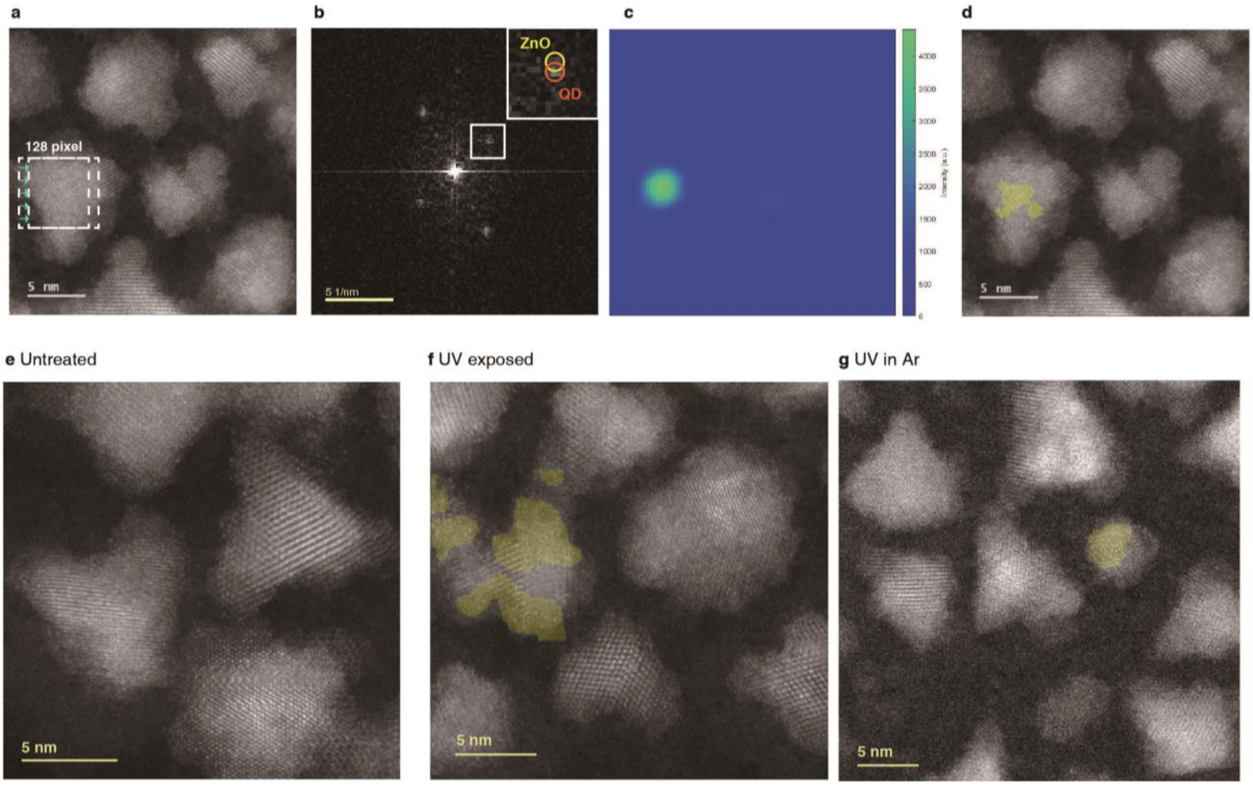

Supplementary Fig. 7 | Detection of oxide formation in QDs. a Representative HAADF-STEM image of QDs after oxidation. **b** A FFT pattern obtained from region marked with white box in (**a**). **c** The result from code operation showing rough detection of regions with ZnO. The region where ZnO domains are identified is indicated with higher pixel values. **d** An inverse FFT of selected ZnO peaks marked with the yellow circle in the inset of (**b**), overlaid on the original HAADF-STEM image (**a**). **e–g** HAADF-STEM images of untreated QDs (**e**), QDs exposed to UV in air (**f**), and QDs exposed to UV in Ar atmosphere (**g**). (yellow regions) ZnO subdomains detected with the described procedure. The details in the code operation of ZnO detection process are described in Methods.

Added text (Methods; page 19–20; in the main text)

Detection of ZnO subdomains in the QDs

The identification of ZnO subdomains within individual QDs was achieved through the HAADF-STEM images by using a custom MATLAB script. Initially, a small segment of the image (typically located at upper-left corner at first) was selected. FFT was then performed on this segment (Supplementary Fig. 7a). The size of the segment and HAADF-STEM images were typically 128 pixel × 128 pixel and 512 pixel × 512 pixel, respectively. From the FFT of a segment, the presence of ZnO was identified (Supplementary Fig. 7b) by detecting a peak corresponding to major lattice spacings of wurtzite ZnO, such as 2.51 Å, 2.65 Å, and 2.85 Å. If crystalline peaks of ZnO were detected in the FFT, a value of "1" was added to the 'matrix'. The 'matrix' is constructed to record information about the ZnO subdomains, with the same dimensions as the STEM image. Initially, all values in the 'matrix' were zero. This procedure was repeated by sweeping through the entire STEM image, moving 1 pixel from left to right. When the segment reached the edge of the image, it moves to the next row (1 pixel

below). The ZnO detection procedure ended when the segment reached the last part of the image, and the 'matrix' is visualized as a heat map. The representative results is shown in Supplementary Fig. 7c. Once the rough location of ZnO is identified, an inverse FFT is performed at the position by crystalline ZnO peaks in the FFT pattern to map the regions with ZnO lattices (Supplementary Fig. 7d).

Added text (Methods; page 9–10; in the main text)

... Furthermore, multiple images are inspected by the crystalline phase analysis to identify ZnO domains in QDs (Supplementary Fig. 7 and Methods), and the population of the QDs with ZnO on their surfaces increases after the UV exposure in air (Fig. 2n). The size distribution of the ZnO subdomains is shown in Fig. 2o. On the other hand, the QDs exposed to UV in an Ar atmosphere exhibit little morphological changes compared to the untreated QDs, as shown in Supplementary Fig. 5 and 8. Small amount of ZnO domains are identified in these QDs exposed to UV in Ar (Fig. 2n, green bar), possibly formed by a trace amount of oxygen in the solvents and is attributed to the decrease in the PLQY (Fig. 1d).

Comment #10:

The manuscript should be carefully proof-read to improve the style and eliminate errors.

Response #10:

We thank the Reviewer #3's for the careful comment on the writing. We carefully revised the manuscript to eliminate typos and to improve writing styles. We ask the reviewer's favor to look into the revised manuscript.

Thank you very much for many appropriate and valuable comments. We are sure that these insightful comments have improved the quality of the manuscript significantly.

REVIEWERS' COMMENTS

Reviewer #1 (Remarks to the Author):

The authors have addressed all of my comments satisfactorily. The revised version is improved significantly. I therefore recommend publication of this paper as it is.

Reviewer #2 (Remarks to the Author):

The authors have well addressed the reviewers'concerns, and now the quality of this manuscript have been obviously improved. I recommend the publication of this paper as is.

Reviewer #3 (Remarks to the Author):

The authors have carefully revised the manuscript and fully addressed all points I raised. I recommend publication of this paper in its present form.
